
JOURNAL INFORMATION
==============================
NLM Title Abbreviation: Fluids Barriers CNS
Journal ID: Fluids Barriers CNS
NLM Journal ID: 101553157

Fluids and Barriers of the CNS

EISSN: 2045-8118
Publisher: BMC

ARTICLE INFORMATION
==============================
PMCID: PMC5791046
DOI: 10.1186/s12987-017-0084-z
Article ID: 84
Article version: 1
Subjects: Meeting Abstracts

Hydrocephalus 2017, the Ninth Annual Meeting of the International Society for Hydrocephalus and CSF disorders (Hydrocephalus Society)

Electronic publication date: 2018 Jan 30
Publication date: 2018
Volume: 15
Issue: Suppl 1
Issue sponsor: Publication of this supplement was funded by the Hydrocephalus Society.
Electronic Location ID: 4
Copyright: © The Author(s) 2018
License: Open AccessThis article is distributed under the terms of the Creative Commons Attribution 4.0 International License (http://creativecommons.org/licenses/by/4.0/), which permits unrestricted use, distribution, and reproduction in any medium, provided you give appropriate credit to the original author(s) and the source, provide a link to the Creative Commons license, and indicate if changes were made. The Creative Commons Public Domain Dedication waiver (http://creativecommons.org/publicdomain/zero/1.0/) applies to the data made available in this article, unless otherwise stated.
License URL: https://creativecommons.org/licenses/by/4.0/

Conference name: Hydrocephalus 2017 - The Ninth Annual Meeting of the International Society for Hydrocephalus and Cerebrospinal Fluid Disorders (Hydrocephalus Society)
Conference location: Kobe, Japan
Conference date: 23-25 September 2017
issue-copyright-statement: © The Author(s) 2018

==============================

## I1
### Introduction
Etsuro Mori
Department of Behavioral Neurology and Neuropsychiatry, Osaka University United Graduate School of Child Development, D51-09, 2-2, Yamadaoka, Suita 565-0871, Japan
**Correspondence:** Etsuro Mori - etrmori@psy.med.osaka-u.ac.jp
*Fluids and Barriers of the CNS* 2018, **15(Suppl 1):**I1

As president, I was delighted to welcome over 300 delegates to the Ninth Meeting of the International Society for Hydrocephalus and Cerebrospinal Fluid Disorders (Hydrocephalus 2017) in Kobe, Japan on September 23–25, 2017. This annual meeting was dedicated to research into hydrocephalus and related disorders and attracted delegates from 27 different countries.

Hydrocephalus 2017 covered a full range of CSF disorders affecting both children and adults. In recent years, idiopathic normal pressure hydrocephalus (NPH) has been particularly heightened as a treatable cause of cognitive impairment and falls in the elderly, and was an important topic of the meeting. Delegates heard keynote lectures by the world's experts on many topics including pediatric hydrocephalus, shunt technology, hydrocephalus diagnosis and pathophysiology and brain imaging.

Two satellite symposia accompanied the main Meeting programme: an Educational Normal Pressure Hydrocephalus Symposium to raise awareness and update knowledge amongst clinicians on the growing importance of NPH, and a session of the International Hydrocephalus Imaging Working Group (IHIWG). We also had a great social program starting with a Welcome Reception, followed on successive evenings by a special Japanese evening and a Gala dinner on a cruise ship. Altogether, we enjoyed a very successful and exciting meeting.

## A1
### Lateral ventricle dilation impacts the other CSF compartments
L. Accrombessi, A. Lokossou, Ol. Baledent
BioFlow Image Processing, University hospital of Picardy Jules Verne, Amiens, France
**Correspondence:** L. Accrombessi
*Fluids and Barriers of the CNS* 2018, **15(Suppl 1)**:A1

**Introduction:** The cerebrospinal fluid (CSF) circulates through the ventricles and the cranial and spinal subarachnoid spaces that are dynamic compartments. According to MONROE-KELLIE's doctrine, the CSF is distributed constantly in compartments which interact with each other. What happen in the other compartments and the CSF flow when laterals ventricles dilate?

**Methods:** 10 hydrocephalus patients underwent a 3T MRI with morphological and phase contrast sequences to investigate the CSF volume and its flow at the aqueductal (SVaque) and spinal (SVspine) levels. The volume of each CSF compartments lateral ventricles (LV), intracranial spaces (IV) (pontine and cerebellum cisterns) and spinal spaces (SS), were estimated by measuring the CSF area in a selected plane crossing each compartment. CSF areas measurements of each patient were normalized to show the distribution of one CSF compartment in front of another. An Heterogeneity coefficient was calculated based on the standard deviation.

**Results:** In our population LV area presented the high heterogeneity 49%, IV heterogeneity was equal to 27% and heterogeneity for the SS was equal to 41%. Paradoxically increase of the LV compartment seems to induce an increase of the IV spaces and indeterminate change in SS which can increase or decrease a lot. SVaque was positively correlated with LV area ($R = 0.63$, $p = 0.04$) whereas SVspine presented no significant correlation with LV area. No significant correlations were measured between CSF flows and the all others areas of CSF.

**Conclusions:** Unlike MONROE-KELLIE's doctrine, the compartments can all increase, so it's the tissue or the venous which should decrease.

## A2
### Excess mortality associated with shunt-dependent hydrocephalus following aneurysmal subarachnoid hemorrhage: a population based study
H. Adams, V. Leinonen, A. Lindgren, J. Frösen, M. von und zu Fraunberg, J. Huttunen, T. Koivisto, J. E. Jääskeläinen, T. J. Huttunen
Neurosurgery KUH NeuroCenter, Kuopio University Hospital, and Faculty of Health Sciences, School of Medicine, Institute of Clinical Medicine, University of Eastern Finland, Kuopio, Finland
**Correspondence:** H. Adams
*Fluids and Barriers of the CNS* 2018, **15(Suppl 1)**:A2

**Introduction:** Compared with the general population, significant excess mortality has been associated with aneurysmal subarachnoid haemorrhage (aSAH), however it is unknown if developing shunt-dependent hydrocephalus (SDHC) increases the long-term mortality burden. We aimed to assess their causes of death and to explore whether SDHC impacts excess mortality in aSAH patients.

**Methods:** All aSAH patients from Eastern Finland from 1990 to 2012 were included. The long-term mortality and causes of death were assessed in aSAH patients surviving at least 1 year after index admission.
**Results:** A total of 1417 long-term survivors were followed up until death or 31 December 2014, with a mean (SD) follow-up of 8 (7) years after aSAH. In long-term aSAH survivors, 239 (17%) patients were shunt-dependent. Five, 10, and 25-year survival rates were 88, 75, and 33% for SDHC patients and 94, 84, and 58% for non-SDHC patients, respectively ($p < 0.001$). A multivariate Cox proportional hazard model confirmed SDHC as an independent risk factor for mortality (HR 1.4, CI 95% 1.05–1.77, $p = 0.02$). For SDHC-patients the leading cause of death was found to be aSAH sequalae (19%) and cardiovascular (14%) related, whereas for non-SDHC patients the leading causes were cardiovascular (27%) and cancer (21%) related. Noticeable higher rates were observed in intracerebral haemorrhage (11% vs. 3%) as a cause of death for SDHC-patients as compared to non-shunted patients.
**Conclusions:** SDHC following aSAH is associated with increased risk of long-term mortality with differential causes of death. Future studies are needed to explore the underlying mechanism predisposing SDHC-patients to adverse outcomes, in order to mitigate the mortality burden of aSAH patients developing shunt-dependency.

## A3

### Magnetic resonance imaging markers for predicting postoperative outcome in iNPH-patients

S. Agerskov, M. Wallin, P. Hellström, C. Wikkelsö, M. Tullberg
Hydrocephalus Research Unit, Department of Clinical Neuroscience and Rehabilitation, Institute of Neuroscience and Physiology, The Sahlgrenska Academy, University of Gothenburg, Gotthenburg, Sweden
**Correspondence:** S. Agerskov
*Fluids and Barriers of the CNS* 2018, **15(Suppl 1)**:A3

**Introduction:** Several studies report on the use of magnetic resonance imaging (MRI) markers, e.g. acute callosal angle and Disproportionately Enlarged Subarachnoid Hydrocephalus (DESH) for predicting outcome after shunt surgery in idiopathic normal pressure hydrocephalus (iNPH) but the use of these markers have not been settled. Our aim was to investigate the predictive value of a number of brain morphological MRI-markers in a large group of iNPH-patients.
**Methods:** One hundred and thirty six patients (aged $70 \pm 9.3$ years) with iNPH subjected to standardized quantification of clinical symptoms before and 3 months after shunt surgery were included in the study. Preoperative T1, FLAIR and flow sensitive images were scored regarding magnitude of ventriculomegaly, white matter lesions, dilatation of Sylvian fissures, compression of convexity sulci, presence of focally dilated sulci and aqueductal flow void sign. The callosal angle was also measured. Outcome was calculated using a composite score.
**Results:** All patients showed signs of ventriculomegaly (median EI 0.41 IQR: 0.37–0.44) and an aqueductal flow void sign. The median CC-angle was 68.8° (IQR: 57.7–80.8). Dilated Sylvian fissures were found in 69%, focally dilated sulci (transport sulci) in 25% and widening of the interhemispheric fissure in 55%. Obliteration of sulci at the convexity was found in 33%. Seventy-four percent of patients improved after surgery but none of the investigated MRI-signs were significant predictors of outcome.
**Conclusions:** No associations between MRI-markers and clinical outcome after shunting were found. These markers, although they may be indicative of iNPH, do not seem to represent mechanisms connected to the reversibility of the syndrome.

## A4

### The phenotype of idiopathic normal pressure hydrocephalus

S. Agerskov, K. Andrén, P. Hellström, C. Wikkelsö, M. Tullberg
Hydrocephalus Research Unit, Department of Clinical Neuroscience and Rehabilitation, Institute of Neuroscience and Physiology, The Sahlgrenska Academy, University of Gothenburg, Gotthenburg, Sweden
**Correspondence:** S. Agerskov
*Fluids and Barriers of the CNS* 2018, **15(Suppl 1)**:A4

**Introduction:** A syndrome of gait, balance, cognitive, and urinary impairment is considered typical for idiopathic normal pressure hydrocephalus (iNPH) but patients show a broad variety of symptoms. No large single center study has in detail described typical symptoms and their reversibility after shunting, which we aimed to accomplish using data from the Gothenburg iNPH database.
**Methods:** Four hundred and twenty-nine iNPH patients (aged $71 \pm 9.5$ years) subjected to standardized clinical assessments before and 3–6 months after surgery were included. Symptoms and signs were analyzed pre- and postoperatively.
**Results:** At diagnosis, 41% of the patients showed impairment in all domains (gait, balance, cognition, urgency) and 72% in at least three domains. Ninety percent had gait disturbances, 75% of these had a broad based and 65% a shuffling gait. Freezing was seen in 30%, almost exclusively together with broad based or shuffling gait. Forty-seven percent had retropulsion, 75% MMSE < 28 and 85% impaired bladder control. Paratonic rigidity was present in 73% and cerebellar signs in 12%. Postoperatively, 75% of the patients improved and impairments were seen in fewer symptom domains.
**Conclusions:** Gait impairment is the predominant symptom in iNPH but patients usually present with symptoms in at least three of four domains. Freezing of gait is associated with broad based or shuffling gait, possibly indicating more advanced disease. Retropulsion and paratonic rigidity are common and a cerebellar disturbance may be part of the syndrome in some cases. All symptoms show reversibility after shunting, but full restitution only occurs in a minority of patients.

## A5

### Assessment of cerebrospinal fluid flow in ventricular shunt system using contrast-enhanced ultrasound

S. R. Aglyamov[1], R. K. Hartman[1], A. Fowler[1], S. Park[2], S. Y. Emelianov[3,4]
[1]Department of Biomedical Engineering, University of Texas at Austin, Austin, TX, USA; [2]School of Electrical and Electronics Engineering, Chung-ang University, Seoul, Korea; [3]Department of Electrical and Computer Engineering, Georgia Institute of Technology, Atlanta, GA, USA; [4]Department of Biomedical Engineering, Georgia Institute of Technology and Emory University School of Medicine, Atlanta, GA, USA
**Correspondence:** S. R. Aglyamov
*Fluids and Barriers of the CNS* 2018, **15(Suppl 1)**:A5

**Introduction:** Current methods for diagnosing ventriculoperitoneal shunt malfunctions often require ionizing radiation, or are expensive for the patient. The overall goal of these studies is to develop a safe, cost-effective method for diagnosing shunt malfunctions and measure cerebrospinal fluid flow in ventriculoperitoneal shunts based on contrast-enhanced real-time ultrasound imaging.
**Methods:** Medtronic ventricular shunt catheters were placed in a flow-mimicking setup and a syringe infusion pump was used to regulate flow through the shunt catheters. Ultrasound images were collected using the Vevo2100 (VisualSonics Inc.) and the Verasonics V1 (Verasonics Ins.) ultrasound systems. An FDA-approved microbubble contrast agent (Optison™) was injected into the shunt system. In addition, we explored acoustic generation of bubbles using a spherically focused, single element ultrasound transducer with a center frequency of 530 kHz. The nonlinear acoustic responses from the microbubbles were investigated to improve flow rate measurement with low microbubble concentrations.
**Results:** Proposed method was able to differentiate between no flow and flow rates between 0.01 and 0.1 ml/min. Bubbles were reliably generated by the focused ultrasound transducer for the optimal acoustic parameters: 3.6 MPa peak negative pressure, 100 Hz PRF, and a 150 µs burst over a 0.5 s duration. Contrast-enhanced nonlinear ultrasound imaging of microbubbles significantly improved detection of the flow.
**Conclusions:** We have demonstrated the feasibility of using both injected and acoustically generated microbubbles and in combination with ultrasound imaging in linear and nonlinear modes to measure fluid flow in shunts.

## A6

### Accelerated cerebrospinal fluid turnover reduces amyloid Β 42 toxic conformer ratio

C. AKiba, M. Nakajima, M. Miyajima, I. Ogino, H. Arai
Juntendo University, Department of Neurosurgery, Tokyo, Japan
**Correspondence:** C. AKiba
*Fluids and Barriers of the CNS* 2018, **15(Suppl 1)**:A6

**Introduction:** Aβ aggregate is key pathological feature of AD, held in equilibrium of the toxic and the non-toxic conformer. Aβ42 toxic conformer quantitative analysis in iNPH patients is not previously reported. We analyzed Aβ42 toxic conformer in CSF obtained from iNPH patients, with postoperative changes in cognitive function.
**Methods:** For the first step, we analyzed Aβ42 toxic conformer in CSF obtained from AD patients ($n = 70$, $73.1 \pm 1.9$ year-old), iNPH patients ($n = 66$, $76.4 \pm 0.6$ year-old), and normal controls ($n = 68$, $75.3 \pm 1.7$ year-old), using 24B3 antibody in ELISA. For the second step, Aβ42 toxic conformer ratio (%) (Aβ42 toxic conformer/total Aβ42 $\times$ 100) was measured pre and postoperatively in iNPH paients.
**Results:** Aβ42 toxic conformer values (pg/ml) and Aβ42 toxic conformer ratio (%) could divide the three diseases with statistical significance ($p < 0.05$). iNPH patients were divided into two groups; patients with postoperative decline of Aβ42 toxic conformer ratio group (decline-group, $n = 40$) and postoperative not-changed or elevated group (not-declined-group, $n = 26$). In decline-group, MMSE were significantly elevated postoperatively ($p < 0.05$), while the change in not-declined-group was not significant ($p = 0.645$).
**Conclusions:** Aβ42 toxic conformer value and ratio in CSF are avail for distinguish iNPH patients, AD patients, and normal controls. Promotion of CSF turnover in iNPH patients by CSF shunting may increase Aβ42 toxic conformer clearance. Postoperative reduction of Aβ42 toxic conformer ratio in CSF was associated with improved cognitive function.

## A7

### Preoperative prognostic value of MRI in 90 patients with suspected idiopathic normal pressure hydrocephalus

L. Albini Riccioli[1], A. F. Marliani[1], G. Napoli[1], F. Villani[1], S. Cevoli[2], G. Giannini[2], G. Palandri[3], P. Mantovani[3], A. Valluzzi[3], F. Oppi[2], D. Milletti[4], R. Agati[1], C. Sturiale[3], P. Cortelli[2]
[1]Operative Unit of Neuroradiology, Institute of Neurological Sciences of Bologna, Bologna, Italy; [2]Operative Unit of Neurology, Institute of Neurological Sciences of Bologna, Bologna, Italy; [3]Operative Unit of Neurosurgery, Institute of Neurological Sciences of Bologna, Bologna, Italy; [4]Operative Unit of Rehabilitation Medicine, Institute of Neurological Sciences of Bologna, Bologna Italy
**Correspondence:** L. Albini Riccioli
*Fluids and Barriers of the CNS* 2018, **15(Suppl 1)**:A7

**Introduction:** The Institute of Neurological Sciences of Bologna developed a protocol study to evaluate morphological and functional markers proposed in the literature for neuroradiological diagnosis of idiopathic normal pressure hydrocephalus (iNPH) to select adequately the candidates to surgery and predict their response to shunt. The aim of our study is to evaluate which morphological parameters are most frequent in patients selected to be treated.
**Methods:** From February 2015 to April 2017, 90 patients were prospectively enrolled in the protocol study: median age was 74.2 years (range 56–86); 55 men (61%) and 35 women (39%). Almost every patient underwent a 3 Tesla MRI. Brain's preoperative MRI were retrospectively evaluated in 19 patients undergoing shunt surgery. The following main parameters were evaluated: ventricular system dilation, trans-ependimal reabsorption, narrowing sulci at the high convexity, increase in Sylvian fissure, callosal angle measurement, third ventricle floor lowering, flow void through the aqueduct and Monro foramina, bi-frontal ratio, bi-caudate and Evan's indexes.
**Results:** Case reviews showed the ventricular system dilation and narrowing sulci at the high convexity in all treated patients. Increase in Sylvian fissure, callosal angle reduction, bi-caudate and Evan's indexes were positive in 18 patients and lowering of third ventricle, flow void and bi-frontal ratio index in 17.
**Conclusions:** From our preliminary experience the discrepancy between the size of the subarachnoid spaces at the convexities near the vertex and the enlarged silvian fissures, with enlarged ventricles, seems to be the most sensitive and specific sign to predict good shunt results.

## A8

### Withdrawn

*Fluids and Barriers of the CNS* 2018, **15(Suppl 1)**:A8

## A9

### Withdrawn

*Fluids and Barriers of the CNS* 2018, **15(Suppl 1)**:A9

## A10

### Withdrawn

*Fluids and Barriers of the CNS* 2018, **15(Suppl 1)**:A10

## A11

### Withdrawn

*Fluids and Barriers of the CNS* 2018, **15(Suppl 1)**:A11

## A12

### Withdrawn

*Fluids and Barriers of the CNS* 2018, **15(Suppl 1)**:A12

## A13

### A comparison of the diagnostic guidelines in normal pressure hydrocephalus

J. Andersson, M. Rosell, K. Kockum, L. Söderström, K. Laurell
Unit of Pharmacology and Clinical Neuroscience, Östersund Hospital, Region Jämtland Härjedalen, Sweden
**Correspondence:** J. Andersson
*Fluids and Barriers of the CNS* 2018, **15(Suppl 1)**:A13

**Introduction:** Idiopathic normal pressure hydrocephalus (iNPH) still lacks common, standardized diagnostic criteria. In this study we aim to evaluate the two diagnostic guidelines in a sample from the general population.
**Methods:** Out of thousand inhabitants aged 65 or older, 168 individuals (93 females, 75 males), mean age 75 years (range 66–92) with and without symptoms of iNPH underwent a neurological and radiological examination. The participants were diagnosed as "unlikely", "possible" and "probable" iNPH according to the American–European and the Japanese guidelines, respectively. Lumbar opening pressure measurements were not performed.
**Results:** Obtaining a diagnosis of "probable iNPH" was three times more likely according to the American–European guidelines ($n = 35$) compared to the Japanese guidelines ($n = 11$). The concordance was fair between the two diagnostic guidelines (Kappa 0.51).
**Conclusions:** Discrepancies were found when diagnosing iNPH according to the two diagnostic guidelines. This could probably be explained by differences in clinical and radiological criteria. As a step towards widely accepted, standardized diagnostic criteria, we suggest a revision of the current guidelines, preferably into one common diagnostic system.

## A14

### Cerebral hydrodynamics and morphology in neurodegenerative diseases, the diagnostic role of PC-MRI

J. Attier-Zmudka[1,4], B. Berteaux[1,] J. M. Sérot[1], S. Dao[2], I. Gyselinck[1], A. S. Macaret[3], O. Balédent[4]
[1]Geriatrics, General Hospital, Saint Quentin, France; [2]Radiology, General Hospital, Saint Quentin, France; [3]Neurology, General Hospital, Saint Quentin, France; [4]BioFlowImage, University Hospital of Picardy Jules Verne, Amiens, France
**Correspondence:** J. Attier-Zmudka
*Fluids and Barriers of the CNS* 2018, **15(Suppl 1)**:A14

**Introduction:** The pathophysiology of cognitive impairments such as Alzheimer disease (AD) and Normal pressure hydrocephalus (NPH) is not fully understood. Structural magnetic resonance imaging (MRI) is considered as the first-line tool next to neuropsychological assessment, to assist with early diagnosis of dementia, showing global cerebral and hippocampal atrophy. Both conditions may occur in physiological aging, malnutrition, depression and chronic inflammation. It has been suggested that both AD and NPH are physiologically related to cerebrospinal fluid (CSF) circulatory failure. Phase contrast MRI (PC-MRI) helps differentiate NPH from other neurodegenerative diseases.
**Methods:** Basing on geriatric and neuropsychological assessment, structural and PC-MRI in 95 elderly, we analyzed the relation between the cerebral hydrodynamics, structural cerebral lesions and cognitive status parameters.
**Results:** MRI revealed hippocampal atrophy in almost 85% of our population; neuropsychological and clinical assessments confirmed AD in less than 30% of the patients. More than 80% of women presented with global cerebral atrophy; however, they do not show a higher prevalence of AD than men. MRI results revealed ventricular dilation in 11% of the study population. Comprehensive clinical and neuropsychological assessment allowed a diagnosis of AD in 50% of these patients. The PC-MRI suspected NPH in 3 patients. Only 1 accepted valve, with clinical success.
**Conclusions:** PC-MRI is a rapid, noninvasive, and still underused method permitting evaluation of vascular and CSF flows and studying their dynamic coupling throughout the cardiac cycle. It can be used to assist with differential diagnoses in neurodegenerative disease.

## A15

### Cardiovascular and sleep apnea syndrome impact in neurodegenerative diseases

J. Attier-Zmudka[1,4], B. Berteaux[1], J. M. Sérot[1], Y. Douadi[2], C. Sorel[3], S. Dao[4], I. Gyselinck[1], O. Balédent[5]
[1]Geriatrics, General Hospital, Saint Quentin, France; [2]Pulmonology, General Hospital, Saint Quentin, France; [3]Cardiology, General Hospital, Saint Quentin, France; [4]Radiology, General Hospital, Saint Quentin, France; [5]BioFlowImage, University Hospital of Picardy Jules Verne, Amiens, France
**Correspondence:** J. Attier-Zmudka
*Fluids and Barriers of the CNS* 2018, **15(Suppl 1)**:A15

**Introduction:** The elderly population is rarely included in research. Sleep apnea syndrome is common in elderly patients, especially in those with dementia. The brain and its vascular system cannot be considered separately from the heart and the lungs, which provide energy and oxygen supply. Our objective was to identify the real prevalence of sleep apnea syndrome, its cardiovascular consequences and correlation with the cognitive status in truly elderly.
**Methods:** Basing on a clinical examination, geriatric and neuropsychological assessment, blood tests, structural magnetic resonance imaging, 24-h ambulatory blood pressure measurement, and electrocardiogram in 95 patients, aged 75 years and older, suffering from neurodegenerative diseases, we analyzed the relation between the cognitive, cardiac, and respiratory parameters.
**Results:** A significant difference between men and women was observed for age, tobacco smoking, overweight, anemia, global cerebral atrophy, dementia stage, myocardial infarction, carotid stenosis, and α1-blocker treatment. Polygraphy revealed sleep apnea syndrome in 68% participants. All correlations between polygraphy parameters and cognitive status variables were significant.
**Conclusions:** This study highlights the discrepancy between the available epidemiological data concerning elderly and our results obtained by a comprehensive, holistic study of patients aged 75 years and older. In a representative group of "truly" elderly individuals, we showed that the prevalence of sleep apnea syndrome is much higher than that reported in the literature. Moreover, the association between the presence of sleep apnea syndrome and cognitive decline, sheds new light on the debate about the etiology of neurodegeneratives diseases, its diagnosis, prevention, and treatment.

## A16

### Cerebral hydrodynamics impact in neurodegenerative diseases

J. Attier-Zmudka[1,4], B. Berteaux[1], J. M. Sérot[1], S. Dao[2], I. Gyselinck[1], A. S. Macaret[3], O. Balédent[4]
[1]Geriatrics, General Hospital, Saint Quentin, France; [2]Radiology, General Hospital, Saint Quentin, France; [3]Neurology, General Hospital, Saint Quentin, France; [4]BioFlowImage, University Hospital of Picardy Jules Verne, Amiens, France
**Correspondence:** J. Attier-Zmudka
*Fluids and Barriers of the CNS* 2018, **15(Suppl 1)**:A16

**Introduction:** There are 2 types of cerebrospinal fluid (CSF) dynamics: the bulk flow with its role to clean the brain from toxic molecules such as Aβ protein, pathognomonic for Alzheimer disease (AD), and the pulsatile flow responsible for maintaining stable intracranial pressure (ICP) during the cardiac cycle (CC). The dynamic index (DI), as a multiplication of cerebrospinal fluid stroke volume at aqueductal and C2–C3 levels, reflects the CSF pulsatile activity between the main CSF compartments measured in microliters per cardiac cycle (µl/CC). It serves as "CSF shaker" by distributing proteins, including those pathognomonic for AD. This new index can be easily assessed with phase contrast magnetic imaging (PC-MRI).
**Methods:** Basing on geriatric and neuropsychological assessment, structural and PC-MRI in 95 elderly, suffering from neurodegenerative diseases, we analyzed the relation between the cerebral hydrodynamics and cognitive status parameters.
**Results:** Mean DI was 28,660 µl/CC ± 34,411, max 157 500, min 0. DI was heterogeneous. Among all the tests corresponding to neuropsychological assessment, we found the significate correlation between DI and episodic memory ($p = 0.04$), praxis ($p = 0.03$) and executive functions ($p = 0.01$).
**Conclusions:** The results of neuropsychological tests, evaluating episodic memory, praxis and executive functions were directly and significantly correlated with DI.

## A17

### Psychiatric disorders are common in patients with idiopathic intracranial hypertension and associate with low cerebrospinal fluid protein

C. Avellan[1], V. Leinonen[2], T. Puustinen[2], J. E. Jääskeläinen[2], S. M. Lehto[3,4], T. Huttunen[2], A. P. Elomaa[2]
[1]Department of Neurosurgery, University of Helsinki and Helsinki University Hospital, Helsinki, Finland; [2]Department of Neurosurgery, NeuroCenter, Kuopio University Hospital, Kuopio Finland; [3]Institute of Clinical Medicine/Psychiatry, University of Eastern Finland, Kuopio, Finland; [4]Department of Psychiatry, Kuopio University Hospital, Kuopio, Finland
**Correspondence:** C. Avellan
*Fluids and Barriers of the CNS* 2018, **15(Suppl 1)**:A17

**Introduction:** Idiopathic intracranial hypertension (IIH) is a rare condition affecting obese woman of childbearing age with unknown pathophysiology. IIH is characterised by raised intracranial pressure, papilledema and visual loss. Studies on IIH patients have illustrated neuroendocrine-axis dysfunctions such as central hypothyroidism, which may contribute to e.g. mood symptoms. Psychiatric symptoms and demographic contributing factors causing the mental health disorders in IIH patients are still unclear. We characterized the presence of psychiatric symptoms of IIH patients in a population-based setting.
**Methods:** All psychiatric diagnoses and drug usage were extracted from patient records of 35 IIH-patients, admitted to Kuopio University Hospital between 2000 and 2016.
**Results:** 80% of patients were females. At the time of diagnosis the mean for age was 30.1 years, for body-mass-index 36.2 kg/m$^2$, for cerebrospinal fluid (CSF) opening pressure 31.2 mmHg, and for follow-up time 4.4 years, respectively. All patients were treated with acetatzolamide and weight loss, 34% were shunted. 46% of the patients use psychopharmaca (antipsychotics, antidepressants or sedatives). The most common psychiatric diagnosis was depression. The presence of any psychiatric disorders was associated with lower CSF protein count at diagnosis (247 vs. 432 mg/l) ($p = 0.045$) compared with patients without psychiatric diagnosis, whereas other findings showed no associations.
**Conclusions:** Half of IIH patients suffer from psychiatric disorders. CSF hypoproteinemia is associated with higher prevalence of the disorders. Our data suggests that psychiatric distress is common in IIH and should be screened for. The underlying pathophysiology could be related to hypothalamus–pituitary–adrenal cortex-axis dysfunction caused by elevated intracranial pressure, but needs to be verified.

## A18

### Association between high biomarker probability of Alzheimer's disease and change of quantitative regional cerebral blood flow after shunt surgery in patients with idiopathic normal pressure hydrocephalus

S. Azuma[1], H. Kazui[1], T. Matsumoto[1], T. Suehiro[1], S. Sato[1], Y. Suzuki[1], H. Kanemoto[1], K. Yoshiyama[1], H. Kishima[2], M. Ikeda[1]
[1]Department of Psychiatry, Osaka University Graduate School of Medicine, Osaka, Japan; [2]Department of Neurosurgery, Osaka University Graduate School of Medicine, Osaka, Japan
**Correspondence:** S. Azuma
*Fluids and Barriers of the CNS* 2018, **15(Suppl 1)**:A18

**Introduction:** In patients with idiopathic normal pressure hydrocephalus (iNPH), change of regional cerebral blood flow (rCBF) after shunt surgery differs from study to study. Recent studies revealed Alzheimer's disease (AD)-related pathology is high prevalent in iNPH patients. There is a possibility that presence of AD-related pathology may involve rCBF change after shunt surgery.
**Methods:** In this study, 39 iNPH patients were classified into 15 patients with (iNPH/AD+) and 24 patients without (iNPH/AD−) combination with low amyloid β42 and high total tau in cerebrospinal fluid (CSF). Quantitative rCBF was quantified in 17 Regions-of-interest (ROIs) by 123I-IMP single photon emission computed tomography (SPECT) using the autoradiography (ARG) method. We compared changes of rCBF 3 months after shunt surgery between iNPH/AD− and iNPH/AD+ by using two-way analysis of variance (ANOVA) for repeated measures. And we compared rCBF of each patient before and 3 months after shunt surgery by using paired *t* test. In both tests, we adjusted p values with Benjamini–Hochberg method for multiple comparisons.
**Results:** We found significant group × shunt effects in rCBF change in the high convexity area, posterior cingulate cortex, precuneus, putamen, amygdala, hippocampus, parahippocampal gyrus, superior parietal cortex, dorsolateral, medial, and orbital of frontal cortices. In iNPH/AD− patients, rCBF increased significantly in the putamen, amygdala, hippocampus, and parahippocampal gyrus. On the other hand, in iNPH/AD+ patients, rCBF increased significantly in no regions.
**Conclusions:** The results of this study may indicate that AD-related pathology involves rCBF change after shunt surgery in iNPH patients.

## A19

### Pulsatility evolution of the blood flow curve through the cranium

O. Baledent[1], A. Lokossou[1], B. Chaarani[1], J. Zmudka[1,2]
[1]BioFlow Image Processing, University Hospital of Picardy Jules Verne, Amiens, France; [2]Geriatric Department, General Hospital, Saint Quentin, France
**Correspondence:** O. Baledent
*Fluids and Barriers of the CNS* 2018, **15(Suppl 1)**:A19

**Introduction:** Systolic cerebral arterial blood flow (ABF) has to be dampened as soon as possible by the cerebral venous outflow (VBF) and by the small but rapid CSF oscillations. Because this pulsatility mechanism is not well understood and could lead to hydrocephalus or intracranial hypertension we propose to quantify the pulsatilitiyes' evolution of the CSF, ABF and VBF through the intracranial compartment.
**Methods:** 19 healthy elderly underwent a 3T MRI to quantify CSF flows in the aqueduct and the spinal canal. Internal carotid, vertebral and the basilar arteries, internal jugular veins, straight and sagittal sinuses were also investigated to quantify ABF and VBF at the intracranial and extracranial levels. Maximal, minimal and mean values of the vascular flows were calculated to obtain a pulsatility index (PI = (max–min)/mean) for ABF and VBF for each intracranial and extracranial level. Based on ABF–VBF the Intracranial blood volume change (IBVC) during the cardiac cycle was calculated.
**Results:** IBVC (1 ± 0.27 ml) was not correlated with aqueduct CSF oscillations (0.053 ± 0.03 ml) but was well correlated (R = 0.75; $p < 0.001$) with the spinal CSF oscillations (0.44 ± 0.24 ml). Intracranial arterial PI (1.1 ± 0.2 [0.8–1.5]) was significantly ($p = 0.01$) smaller than extracranial arterial PI (1.3 ± 0.3 [0.9–2]). Extracranial venous PI (0.8 ± 0.3 [0.3–1.3]) was significantly ($p = 0.02$) higher than intracranial venous PI (0.5 ± 0.2 [0.3–0.8]).
**Conclusions:** CSF oscillations reflect how the cerebral venous outflow flushes the arterial input flow. Poorly investigated, cerebral venous flows play an important role in understanding CSF dynamics physiology and its pathologies.

## A20

### A Transvenous pressure gradient differentiates hydrocephalus from idiopathic intracranial hypertension

G. A. Bateman[1,2], A. R. Bateman[3]
[1]Medical Imaging, John Hunter Hospital, Newcastle, Australia; [2]Faculty of Health, University of Newcastle, Newcastle, Australia; [3]Biomedical Engineering, University NSW, Sydney, Australia
**Correspondence:** G. A. Bateman
*Fluids and Barriers of the CNS* 2018, **15(Suppl 1)**:A20

**Introduction:** Chronic hydrocephalus is associated with normal intracranial pressure (ICP) but dilated ventricles. Idiopathic intracranial hypertension (IIH) is associated with elevated ICP but normal ventricles. This apparent paradox has largely remained unexplained. It is suggested a pressure gradient between the superficial and deep venous territories of the brain could account for the variation between the two diseases.
**Methods:** Using MR phase contrast flow quantification the mean blood flow rate and the cross-sectional area of the sagittal sinus and the straight sinus were measured in 21 patients with hydrocephalus, 20 patients with IIH and 20 age matched controls. The pressure drop from the midpoint of the sagittal sinus and the end of the straight sinus to the torcular was estimated using a modified Poiseuille's equation.
**Results:** The pressure gradient between the two sinuses was zero in controls. The gradient was elevated by 1 mmHg in hydrocephalus and reduced by 0.25 mmHg in IIH.
**Conclusions:** The lack of a pressure gradient between the superficial and deep venous territories in controls would tend to imply a balance between interstitial fluid production/absorption between the two. In hydrocephalus a reduction in pressure in the deep territory compared to the superficial would promote CSF absorption across the ventricular ependyma and ventricular dilatation would ensue even if the ICP were normal. In IIH the higher pressure in the deep territory compared to the superficial would tend to make absorption of CSF through the ependyma unfavorable despite an elevation in ICP and therefore the ventricles would not dilate.

## A21

### Advancing technology in hydrocephalus: aqueduct critical care smart external drain device (SED): 1st in man experience in North America

S. R. Browd[1,2,3,4]
[1]Professor of Neurological Surgery, University of Washington, Seattle, WA, USA; [2]Attending Neurosurgeon, Seattle Children's Hospital, Seattle, WA, USA; [3]Director, Hydrocephalus Program Seattle Children's, Seattle, WA, USA; [4]Co-founder & CMO, Aqueduct Critical Care, Bothell, WA, USA
**Correspondence:** S. R. Browd
*Fluids and Barriers of the CNS* 2018, **15(Suppl 1)**:A21

**Introduction:** The management of ventricular catheters has been relatively unchanged over the last 60 years. Gravity depended systems are very limited and fraught with potential human error related to under or overdrainage. New technologies are needed to advance EVD care, reduce errors and patient risk while reducing cost of care.
**Methods:** Over the last 6 years, we have developed an automated EVD management system that is able to regulate to ICP or flow rate, allow the patient to mobilize based on a fixed reference point and has highly sensitive controlling algorithms. We report on the 1st clinical trial in North America using the Aqueduct SED.

**Results:** The results of 1st in man trials of the Aqueduct SED will be discussed as the device is utilized in 10 high volume Neurosurgical centers across North America.

**Conclusions:** Technology is needed to improve the management of CSF drainage and provide safer patient care while reducing the cost of care provision. We report on the 1st in man experience using the Aqueduct Critical Care Automated External Drain device from experience in high volume North American Neurosurgical practices.

## A22

### Sarcopenia in idiopathic normal-pressure hydrocephalus

M. Bundo, M. Tanimoto, S. Nishizaki, S. Owaki
Department of Neurosurgery, National Center for Geriatrics and Gerontology, Obu, Japan
**Correspondence:** M. Bundo
*Fluids and Barriers of the CNS* 2018, **15(Suppl 1)**:A22

**Introduction:** Sarcopenia is a geriatric syndrome in which loss of muscle mass and strength impairs physical function and Activity of Daily Living (ADL). Long-term disability in idiopathic normal-pressure hydrocephalus (iNPH) may cause sarcopenia. This study investigated the prevalence and influence of sarcopenia in iNPH.

**Methods:** Participants comprised 123 patients with probable iNPH. Dual-energy X-ray absorptiometry and skeletal muscular index (SMI) were used to assess sarcopenia. Grip and knee extension power, modified Rankin scale (mRS), Mini-Mental State Examination (MMSE), instrumental ADL (IADL), Mini Nutritional Assessment (MNA), serum markers, and shunt response (ΔmRS), and SMI were evaluated using multiple regression analysis.

**Results:** Prevalence of sarcopenia was 45.7% in females and 54.2% in males. SMI correlated with grip power ($p < 0.005$), serum hemoglobin ($p < 0.05$), MNA ($p < 0.05$), and ΔmRS ($p < 0.05$). In patients under 80 years old, SMI also correlated with MMSE ($p < 0.01$) and IADL ($p < 0.05$), and mRS was associated with grip ($p < 0.0005$), knee extension ($p < 0.01$), and muscle volumes of the arms ($p < 0.05$) and legs ($p < 0.05$). In patients over 80 years old, mRS correlated with SMI ($p < 0.05$), grip ($p < 0.01$), knee extension ($p < 0.05$), and muscle volume of the legs ($p < 0.05$).

**Conclusions:** Prevalence of sarcopenia was quite high in iNPH. Sarcopenia can progress disease severity and reduce shunt response. These results strongly recommend early diagnosis and surgery.

## A23

### Validating automated parcellation of the sub-components of the ventricular system in normal pressure hydrocephalus patients

A. Carass[1], M. Shao[1], X. Li[1], J. Shin[2], S. Roy[3], J. L. Prince[1], A. M. Blitz[2], L. M. Ellingsen[1,4]
[1]Department of Electrical and Computer Engineering, Johns Hopkins University, Baltimore, MD, USA; [2]Department of Radiology and Radiological Science, Johns Hopkins Hospital, Baltimore, MD, USA; [3]CNRM, The Henry M. Jackson Foundation for the Advancement of Military Medicine, Bethesda, MD, USA; [4]Department of Electrical and Computer Engineering, University of Iceland, Reykjavik, Iceland
**Correspondence:** A. M. Blitz
*Fluids and Barriers of the CNS* 2018, **15(Suppl 1)**:A23

**Introduction:** Standard automated neuroimage processing, such as FreeSurfer, has long played an important role in discovery within various patient groups. However, such standard tools can encounter difficulties when processing normal pressure hydrocephalus (NPH) patients.

**Methods:** We present a validation of a recently developed atlas and patch based segmentation method. The method can determine the extents of the ventricular system and identify sub- components including the lateral (left and right), third, and fourth ventricles. We focus on validating the automated approach in comparison to FreeSurfer and other standard processing tools on our cohort consists of 45 NPH patients that have been manually parcellated.

**Results:** We use both volumetric and overlap comparisons to evaluate our method on the 45 NPH patients. We show statistically significant improvement in accuracy at estimating the volumes of the various sub-components, over state-of-the-art methods.

**Conclusions:** Developing methods for automated neuroimage processing is an important step for systematic evaluation of different patients groups. The high-throughput segmentation method presented and validated here enables such systematic evaluation of brains with ventriculomegaly. This method could be used to elucidate novel patterns of the ventricular system, such as disproportionate dilation of different sub-compartments in NPH patients. A secondary observation of this work is the gross inadequacies of current neuroimage processing methods for ventricle segmentation; moreover, previously reported automated evaluation of NPH patient using FreeSurfer that do not include manual review should be reviewed to ensure their validity.

## A24

### A 3-D finite element study of a bioimpedance-based sensor to record cerebrospinal fluid pulsation

C. Castelar, A. Arslanova, S. Leonhardt, B. J. Misgeld
Philips Chair for Medical Information Technology, RWTH Aachen University, Aachen, Germany
**Correspondence:** C. Castelar
*Fluids and Barriers of the CNS* 2018, **15(Suppl 1)**:A24

**Introduction:** Changes in craniospinal dynamic compliance have been hypothesized to be an important factor in the development of normal pressure hydrocephalus (NPH). A bioimpedance-based sensor integrated onto a drainage catheter could allow the direct monitoring of ventricular volume to be used as an additional control parameter in a smart shunt system. It may also provide further insight into craniospinal dynamics by recording the pulsation of the CSF-filled compartments.

**Methods:** A 3-D finite element (FE) study has been conducted using COMSOL Multiphysics software on geometrical and anatomical models of the brain. Frequency dependent dielectric properties were assigned to the modelled tissues. Multiple ventricle sizes were generated. The sensor consists of a drainage catheter with six integrated ring electrodes on its surface. CSF-pulsation profiles obtained from literature were assigned to the ventricle wall boundaries. An alternating current with amplitude of 10 µA at frequencies from 1 kHz to 1 MHz was injected through the outer electrodes. The resulting current was calculated by integration of the current density and the voltage recorded at the remaining electrodes.

**Results:** The bioimpedance signal correlates to ventricular size and the pulsatile profiles could be extracted from the measured dynamic impedance.

**Conclusions:** The generated FE model can be used to assess the dynamic impedance properties of the CSF space and may be used to study changes in the impedance signal caused by diseases such as NPH. Future work includes the hardware implementation of the measurement system and its validation on a craniospinal bioimpedance test-bench.

## A25

### Surgical outcome of 610 patients with idiopathic normal pressure hydrocephalus treated by lumboperitoneal shunt

C. C. Chang[1], Y. Jimi[2], K. Sanpei[3]
[1]Iyemasa Neurosurgical Clinic, Yokohama, Japan; [2]Noushinkeigeka Higashiyokohama Hospital, Yokohama, Japan; [3]Nishiyokohama International Hospital, Yokohama, Japan
**Correspondence:** C. C. Chang
*Fluids and Barriers of the CNS* 2018, **15(Suppl 1)**:A25

**Introduction:** The aim of this study is to investigate the surgical outcome of the patients with idiopathic normal pressure hydrocephalus (iNPH) treated by the lumboperitoneal (LP) shunt using the Codman Hakim programmable valve (CHPV) with siphonguard.

**Methods:** 610 patients were managed between January 2011 and April 2017. The conventional CHPV was utilized in 528 patients and the CODMAN CERTAS Plus valve in 82. The CODMAN CERTAS Plus valve has 8 different performance settings. Setting 8 is intended to limit flow through the valve. Valve pressure was raised to the maximum of 200 mm H2O or Setting 8, when subdural hematoma (SDH) was detected.
**Results:** A favorable response to the surgery was detected in 96.4%. Malfunction was observed in 27 (4.4%) patients. Lumbar catheter-related; displacement in 13, disconnection in 4, and obstruction in one. Peritoneal catheter-related; disconnection in 6 and displacement in one. Obstruction of the valve was found in one patient. Complications occurred in 16 patients. Surgery-required SDH due to overdrainage was found in 10 (1.6%) patients treated by the system incorporating the conventional CHPV, and lumbar pain followed by catheter removal in 6 (1.0%). Infection occurred in 3 patients (0.5%); meningitis in 2, operative wound infection in one.
**Conclusions:** It is safe and useful to treat iNPH by using the LP shunt system incorporating with CHPV. The use of the CODMAN CERTAS Plus valve seems to be very helpful to reduce the incidence of surgery-required SDH by raising valve pressure to the maximum of Setting 8.

## A26

### Reverse of fronto-parietal cortical perfusion on SPECT after shunting in idiopathic normal pressure hydrocephalus: a case report

K. T. Chen[1], J. S. Chiu[2]
[1]Department of Neurosurgery, Chang Gung Memorial Hospital at Linkou, Taoyuan, Taiwan; [2]Department of Nuclear Medicine, Saint Paul's Hospital, Taoyuan, Taiwan
**Correspondence:** K. T. Chen
*Fluids and Barriers of the CNS* 2018, **15(Suppl 1)**:A26

**Introduction:** A two-layer sign in sagittal plane has been proposed as a signature of idiopathic normal pressure hydrocephalus (iNPH) by the two-tailed view analysis using easy Z-score imaging system (eZIS) on brain perfusion SPECT. However, frontal dominant or diffuse cerebral hypoperfusion are more common. Previous studies have documented improved or normalized perfusion after a spinal tap or shunting. Here, we report a definite iNPH patient presented with a reverse of fronto-parietal cortical perfusion after a shunt surgery.
**Methods:** Case report
**Results:** A 69-year-old woman was evaluated because of 6 months of progressive gait disturbance, urinary incontinence and memory decline. Her Japenese NPH Grading Scale-Revised (JNPHGSR) was 8 (gait:2, cognition:3, urinary:3). A brain CT revealed ventriculomegaly, periventricular lucency and disproportionately enlarged subarachnoid space. A SPECT showed a two-layer structure consisting of decreased blood flow around the corpus callosum and increased perfusion outside cingulate gyrus. A follow-up SPECT was performed 1 week after shunting. A reverse of blood flow from hyperperfusion to hypoperfusion in fronto-parietal cortical area, associated with a significant normalization of blood flow in deep nuclei was observed. The postoperative JNPHGSR was 4 (gait:1, cognition:2, urinary:1) at 1 week and 2 (gait:1, cognition:1) at 1 month, respectively.
**Conclusions:** We reported an interesting case of perfusion inversion in an iNPH patient. We believe this finding may imply that two-layer sign represents an acute exaggerated stage based on a rapid and near total recovery in our patient; while cortical hypoperfusion could probably be a representative of preclinical or decompensated stage of iNPH. Informed consent to publish had been obtained from the patient.

## A27

### Relationship between 24-h intracranial pressure and venous sinus pressure

C. L. Craven, S. Thompson, L. D'Antona, J. Funnell, L. T. Thorne, A. K. Toma, L. D. Watkins
Victor Horsley Department of Neurosurgery, National Hospital for Neurology and Neurosurgery, Queen Square, London, UK
**Correspondence:** C. L. Craven
*Fluids and Barriers of the CNS* 2018, **15(Suppl 1)**:A27

**Introduction:** The correlation between intra-cranial pressure (ICP) and superior sagittal sinus pressure (SSSP) has not been clearly defined in humans. At this single centre, patients with idiopathic intracranial hypertension (IIH) are investigated with 24-h ICP monitoring (ICPM) and, if there is a known sinus stenosis, catheter venography and manometry. We aim to determine the relationship of the 24-h ICPM values with SSSP.
**Methods:** Single centre cohort of IIH patients (2010–2016) who underwent both ICPM and a single catheter manometry (under local anaesthetic) measurement of SSSP. The relationship between ICPM and SSSP, linear and nonlinear regression models were tested to compare best-fit of the data. Goodness of fit was determined by $R^2$ and fits were compared with F test.
**Results:** Twenty-two patients with IIH (21 F: 1 M), mean age 36.0 ± 9.41 years (mean ± SD). The ICPM was 10.01 ± 3.40 mmHg and SSSP was 28.4 ± 9.57 mmHg. The best-fit line of ICPM and SSSP was a sigmoidal curve ($R^2 = 0.67$, $p < 0.04$), with the least-fit being linear ($R^2 = 0.52$, $p < 0.05$). Patients with ICPM 5–10 mmHg had a stable SSSP of 21 mmHg. Patients with ICPM > 13 mmHg had a mean SSSP of 37 mmHg, but with far greater variability (range 29–51 mmHg).
**Conclusions:** Our results suggest a non-linear relationship between ICP and SSSP. There appears to be a window of ICP (10–13 mmHg) in which SSSP increases rapidly with ICP. SSSP was relatively stable when ICP was between 5 and 10 mmHg. Further research is needed to define the correlation between SSSP and ICP.

## A28

### Lumbar drains can affect CSF protein biomarker levels

C. L. Craven[1], L. D'Antona[1], S. Thompson[1], M. D. Chapman[2], J. Funnell[1], L. T. Thorne[1], M. P. Lunn[2], H. Zetterberg[3,4], A. K. Toma[1], L. D. Watkins[1]
[1]Victor Horsley Department of Neurosurgery, National Hospital for Neurology and Neurosurgery, Queen Square, London, UK; [2]Department of Neuroimmunology, National Hospital for Neurology and Neurosurgery, Queen Square, London, UK; [3]Department of Molecular Neuroscience, UCL Institute of Neurology, Queen Square, London, UK; [4]Department of Psychiatry and Neurochemistry, Institute of Neuroscience and Physiology, the Sahlgrenska Academy at the University of Gothenburg, Mölndal, Sweden
**Correspondence:** C. L. Craven
*Fluids and Barriers of the CNS* 2018, **15(Suppl 1)**:A28

**Introduction:** Normal pressure hydrocephalus (NPH) is commonly investigated with a lumbar drainage (LD) protocol. Cerebrospinal fluid (CSF) from LD may be analysed for neurodegenerative markers. We investigate the effect of both silver-lined (Silverline®, Spiegelberg, GMBH & Co.) and barium-impregnanted (EDM, Medtronic) lumbar catheters on concentrations of AB1-42 and T-tau in CSF.
**Method:** CSF was collected from an individual of unknown disease via lumbar puncture (LP). CSF was thawed at 21 °C and centrifuged for 5 min. A volume of 800uL was injected into (1) 2 ml aliquot (control), (2) barium-lined LD 80 cm (3) silver-lined LD 80 cm. Drains were clamped for 5 min before allowing the CSF to drain into 2 ml aliquots. Each test was repeated three times. ELISA assays determined concentrations of AB1-42 and T-tau, with a total protein (TP) control. Measurement of uncertainly (MU) in T-tau and AB1-42 is 3–47 and 9–17 pg/ml respectively. ANOVA (Geisser-Greenhouse correction) determined significance.
**Results:** Mean CSF concentrations (pg/ml) were corrected for TP. Percentage reduction in AB1-42 pg/ml for barium-impregnated catheters and silver-lined catheters are 9.3% (range 7.4–11.1) and 20.5% (range 18.7–22.3) respectively. T-tau levels were not significantly altered.

| | Control | Barium LD | Silver LD | p-value: (after MU correction) |
|---|---|---|---|---|
| T-tau pg/ml | 638 | 631 | 714 | 0.333 |
| AB1-42 pg/ml | 1737 | 1575 | 1381 | 0.001 |

**Conclusions:** CSF concentrations of AB1-42 can be significantly reduced if taken from a previously clamped or slow draining barium-impregnated or silver-line catheter. Further studies into the effect of silver-lined drains on commonly used biomarkers is required.

## A29

### Intraparenchymal intracranial pressure monitoring for hydrocephalus and cerebrospinal fluid disorders: what are the principal components?

A. Chari, D. Dasgupta, A. Smedley, C. Craven, E. Dyson, S. Matloob, S. Thompson, L. Thorne, A. Toma, L. Watkins
Victor Horsley Department of Neurosurgery, National Hospital for Neurology and Neurosurgery, Queen Square, London, UK
**Correspondence:** A. Chari
*Fluids and Barriers of the CNS* 2018, **15(Suppl 1)**:A29

**Introduction:** Elective intraparenchymal intracranial pressure (ICP) monitoring is useful for the diagnosis and treatment of hydrocephalus and cerebrospinal fluid (CSF) disorders. This retrospective study analyses ICP and pulse amplitude (PA) recordings in neurosurgically naïve patients undergoing elective ICP monitoring for suspected CSF disorders.
**Methods:** Retrospective review of prospectively collated database of neurosurgically naïve patients undergoing elective ICP monitoring for suspected hydrocephalus and CSF disorders. Following extraction of the median ICP and PA values (separated into all, day and night time recordings), principal component analysis (PCA) was performed to identify the principal factors determining the spread of the data. Using the results of the PCA, exploratory comparisons were made of ICP and PA values between post hoc diagnostic groups.
**Results:** A total of 198 patients were identified in 6 distinct diagnostic groups (n = 21–47 in each). The PCA suggested that there were two main factors accounting for the spread in the data (Eigenvalue > 1). Factor 1 accounted for 61.4% of the variance and was determined largely by the PA recordings. Factor 2 accounted for 33.0% of the variance and was determined largely by the ICP recordings. Exploratory comparisons of PA and ICP between the diagnostic groups showed significant differences between the groups. Specifically, significant differences were observed in PA between a group deemed to have 'grossly normal dynamics' and the chiari/syrinx, IIH and NPH/LOVA groups and in the ICP between the 'grossly normal dynamics' group and high-pressure, IIH and low-pressure groups.
**Conclusions:** This study provides insights into hydrodynamic disturbances in different diagnostic groups of patients with CSF hydrodynamic disorders. It highlights the utility of analysing the both median PA and ICP recordings, stratified into day and night time recordings.

## A30

### A classification for chronic hydrocephalus in adults

C. L. Craven, R. Ramkumar, L. D'Antona, S. Thompson, J. Funnell, L. T. Thorne, L. D. Watkins, A. K. Toma
Victor Horsley Department of Neurosurgery, National Hospital for Neurology and Neurosurgery, Queen Square, London, UK
**Correspondence:** C. L. Craven
*Fluids and Barriers of the CNS* 2018, **15(Suppl 1)**:A30

**Introduction:** Ventriculomegaly in the absence of raised intracranial pressure (ICP) is a known entity in adult hydrocephalus practice. The natural history is however, poorly defined. We aim to elucidate the typical demographics, characteristics and outcomes in chronic adult hydrocephalus.
**Methods:** Cluster analysis of adults with ventriculomegaly in the absence of clinical and radiological features of raised ICP (December 2012–May 2016). Records were reviewed to determine symptoms, interventions, Evan's index, ICP monitoring and outcomes. Hierarchical agglomerative clustering with complete linkage was applied.
**Results:** 79 patients (43 F: 36 M) with follow up 69.7 ± 42.4 months (mean ± SD). Four significantly different patient clusters with common features were identified (p < 0.005). Results are presented as: n, mean age, mean Evan's Index, predominant symptoms, median ICP (mmHg) respectively.
(A) **Incidental ventriculomegaly:** n = 17, 39.8 years, 0.45, predominately asymptomatic, brain imaging for unrelated indication, 3.1 mmHg.
(B) **Decompensated hydrocephalus:** n = 18, 48.3 years, 0.47, headache, GCS deterioration, cognitive issues, 14.0 mmHg.
(C) **Early presenting:** n = 20, 28.9 years, 0.55, headache, gait, seizures and cognitive decline, 4.0 mmHg, abnormal pulsatility commonly observed on 24-h ICP monitoring.
(D) **Late presenting:** n = 24, 51.6 years, 0.51, headache, cognition, gait, incontinence, 1.71 mmHg.
Cerebrospinal fluid (CSF) diversion resulted in symptomatic improvement in 70% and over-drainage complications in 4% in groups B–D. Endoscopic third ventriculostomy (ETV) had a high failure rate of 50% of those performed in group C (during adolescence) being later converted to shunts.
**Conclusions:** We describe four distinct sub-types of chronic ventriculomegaly, discovered using an un-biased cluster algorithm. These phenotypes have characteristic demographics, symptoms, ICP results and outcomes.

## A31

### Lower breakpoint of intracranial amplitude-pressure relationship in NPH

Z. H. Czosnyka[1], A. D. Lalou[1], E. Nabanja[1], J. D. Pickard[1], M. Garnett[1], N. C. Keong[2], E. A. Schmidt[3], D. J. Kim[4], M. Czosnyka[1]
[1]Academic Neurosurgery, Cambridge University Hospital, Cambridge, UK; [2]Department of Neurosurgery, National Neuroscience Institute and Duke-NUS Medical School, Singapore, Singapore; [3]Neurosurgery, Purpan Hospital, Toulouse, France; [4]Department of Brain and Cognitive Engineering, Korea University, Seoul, Korea
**Correspondence:** Z. H. Czosnyka
*Fluids and Barriers of the CNS* 2018, **15(Suppl 1)**:A31

**Introduction:** The relationship between intracranial pulse amplitude (AMP) and mean ICP has been previously described; generally stating that AMP increases proportionally to rise in ICP. Such an increase in AMP can be observed particularly often (but not exclusively), if the rise in ICP is provoked by controlled CSF volume increase during the infusion test. We studied lower breakpoint (LB) of amplitude-pressure relationship below which pulse amplitude stays constant when ICP varies. Theoretically, below this breakpoint, the pressure–volume relationship is linear (good compensatory reserve) and above-exponential (brain compliance decreasing with rising ICP).
**Methods:** 169 infusion tests performed in patients diagnosed for NPH (2004–2013) were available for analysis. Inclusion criteria: patients have been shunted and response to shunting was assessed in follow-up clinic; raw data of ICP digital recording (ICM+ software) were available for post hoc processing.
**Results:** Lower breakpoint was observed in 62 patients diagnosed for NPH. Improvement in patients in whom LB was recorded was 77% versus 90% in patients where LB was not recorded (p < 0.02). In patients with detected LB, the difference between baseline ICP and LB was greater in patients who improved (improved: 4.1 ± 2.1 mmHg versus not-improved: 1.2 ± 2.7 mmHg; p < 0.02). There was no correlation between improvement and slope of amplitude-pressure characteristic above LB.
**Conclusions:** The presence of lower breakpoint is associated with less frequent improvement after shunting in NPH. It may be interpreted that CSF dynamics of patients working on flat (linear) part of pressure–volume curve, are more frequently caused by brain atrophy and this is the component less remediable with shunting.

## A32

### Laboratory testing of Pressio®2 intracranial pressure monitor

Z. H. Czosnyka, P. Smielewski, A. D. Lalou, M. Czosnyka
[1]Cambridge Shunt Evaluation Laboratory and Academic Neurosurgical Unit, Addenbrooke's Hospital, Cambridge, UK
**Correspondence:** Z. H. Czosnyka
*Fluids and Barriers of the CNS* 2018, **15(Suppl 1)**:A32

**Introduction:** The aim of this evaluation is to provide extensive bench testing to evaluate the Pressio®2 ICP Monitoring System. Where it was possible absolute accuracy was assessed, otherwise comparison to Codman Express ICP monitor was studied
**Methods:** Drift of pressure measurement over 7 days, drift with temperature, frequency response and accuracy of measurement of both static and pulsatile pressures have been tested simultaneously in Pressio®2 and Codman transducers using a standard laboratory rig. Connectivity of the systems was also assessed.

**Results:** Long term (10 days) zero drift was less than 2 mmHg in both transducers. Temperature drift of Pressio®2 was low (0.2 mmHg per 20 °C). The frequency bandwidth of the Pressio®2 was 20 Hz (relative to Codman transducer). Absolute static accuracy of Pressio®2 was better than 2 mmHg over the range from − 30 to 120 mm Hg. Pulse waveform accuracy (relative to Codman) was better than 0.2 mmHg over the range from 1 to 20 mmHg. The frequency bandwidth of the Pressio®2 was 17 Hz (relative to Codman transducer). Temperature drift of Pressio®2 was low (0.2 mmHg per 20 °C). Pressio®2 ICP Monitor can transmit data directly to external computer without use of pressure transducer bridge amplifier with the resolution of 0.1 mmHg. It is 'ICM+' compatible.

**Conclusions:** The new Pressio®2 ICP Monitor has good accuracy, low zero and temperature drift and excellent frequency properties. The monitor is comparable with Codman Express Monitor and may be, from metrological point of view, used as an alternative in clinical ICP monitoring.

## A33

### Spontaneous retinal venous pulsation with OCT device: towards non-invasive assessment of intracranial pressure

L. D'Antona[1], J. McHugh[2], L. Thorne[1], L. Watkins[1], A. Toma[1], F. Bremner[2]
[1]Victor Horsley Department, The National Hospital for Neurology and Neurosurgery, London, UK; [2]Neuro-ophthalmology Department, The National Hospital for Neurology and Neurosurgery, London, UK
**Correspondence:** L. D'Antona
*Fluids and Barriers of the CNS* 2018, **15(Suppl 1)**:A33

**Introduction:** The absence of spontaneous retinal venous pulsation (SVP) is believed to be an indirect sign of raised intracranial pressure (ICP) and therefore it could represent a useful tool for non-invasive assessment of ICP in patients with disturbances of CSF dynamics. We present the results of a single centre pilot study on the correlation between ICP and SVP.

**Methods:** This is a prospective single centre study. All patients having continuous ICP monitoring who underwent neuro-ophthalmology examination were included. An SVP assessment via both fundoscopy and Spectralis OCT scanner was performed. The Spectralis OCT video recordings were assessed by two experienced neuro-ophthalmologists who were blinded to the ICP monitoring results, and the SVP was graded 0 (absent) to 3 (collapse).

**Results:** Twenty-five consecutive patients (5 M:20F, mean age 40, range 17–72) were selected. On review of the OCT video recordings, 16 patients had bilateral SVP (grade $\geq$ 1). Two patients had unilateral SVP only and 3 had no SVP bilaterally. Bilateral absence of SVP has a 100% sensitivity and a specificity for 24 h median ICP $\geq$ 8.5 mmHg.

**Conclusions:** Our preliminary data suggest that absence of SVP is a good indirect sign of raised median ICP. Larger prospective studies will be needed to confirm these results.

## A34

### Spontaneous intracranial hypotension syndrome: a systematic review of clinical presentation, radiology and treatment outcomes

L. D'Antona[1], S. Thompson[1], J. Funnell[1], C. Craven[1], J. Varghese[1], L. Thorne[1], L. Watkins[1], M. Matharu[2], A. Toma[1]
[1]Victor Horsley Department, The National Hospital for Neurology and Neurosurgery, London, UK; [2]Neurology Department, The National Hospital for Neurology and Neurosurgery, London, UK
**Correspondence:** L. D'Antona
*Fluids and Barriers of the CNS* 2018, **15(Suppl 1)**:A34

**Introduction:** Spontaneous intracranial hypotension (SIH) is a syndrome due to a spontaneous leak of CSF from the spine. Despite many patients can improve with simple medical therapies or with epidural blood patches (EBP), numerous patients fail to respond to the above treatments and represent a great challenge for neurologists and neurosurgeons involved in their care. With our systematic review we describe the clinical and radiological presentation of this syndrome and analyze the treatment outcomes.

**Methods:** This is a PRISMA statement compliant systematic review of the literature. Only original studies in English language with at least 10 patients were included in this review.

**Results:** 118 papers were selected according to the inclusion criteria. This review includes a population of 3955 patients with a mean age of 43.7 years, 37.4% male. The most common symptoms were: orthostatic headache (91%), Nausea/vomiting (38%), Neck pain/stiffness (18%) and hearing disturbances (16.1%). The most common sign on brain MRI with contrast was the typical diffuse pachymeningeal enhancement (63%). 5% of the patients had a completely normal brain MRI. Of the 722 patients with a reported lumbar puncture 63% had a low CSF opening pressure (below 60 mmH2). Only 18% of the patients improved with medical treatment, 79% underwent EBP and of these 69% improved after the first EBP. 4% had surgical treatment.

**Conclusions:** Spontaneous intracranial hypotension (SIH) is a clinical entity with ill-defined intracranial pressure definition. Intracranial pressure monitoring should be considered in patients with atypical clinical presentation and failure to respond to EBP.

## A35

### Paradoxical effect of valve setting adjustment on icp in hydrocephalus patients

L. D'Antona, S. Thompson, A. Chari, C. Craven, J. Funnell, L. Thorne, L. Watkins, A. Toma
Victor Horsley Department, The National Hospital for Neurology and Neurosurgery, London, UK
**Correspondence:** L. D'Antona
*Fluids and Barriers of the CNS* 2018, **15(Suppl 1)**:A35

**Introduction:** Adjustable valve settings are often modified in order to achieve symptomatic improvement in patients with CSF dynamic disturbances. The common knowledge is that increasing the opening pressure of a valve is likely to result in an increase of the intracranial pressure (ICP), however this has not been verified in vivo. We hereby show the results of such analysis in a cohort of patients with shunts undergoing continuous ICP monitoring in our centre.

**Methods:** Retrospective review of prospectively built ICP monitor database. Patients admitted to our hospital for continuous ICP monitoring having at least one valve adjustment during their monitoring were selected. Demographics, clinical details, valve details, CT scans and ICP results were analysed.

**Results:** Forty-three patients having a total of 78 valve setting adjustments during their continuous ICP monitoring were selected (mean age 39, 12 M). Increasing the valve setting resulted in a decrease of the median ICP in 33.3% of the cases (Mean ICP change of paradoxical response cases − 1.7 mmHg ± 1.14). Similarly reducing the valve setting resulted in an increase of the median ICP in 26.5% of the cases (Mean ICP change of paradoxical response cases 2.84 mmHg ± 2.8). Most people with paradoxical change had small ventricles on CT scan.

**Conclusions:** Paradoxical change in ICP following adjustable valve setting change happens in a significant proportion of cases and this might have significant implications on clinical management of patients with CSF dynamic disturbances.

## A36

### Reflow system: new shunt technology for non-invasive intervention for ventricular shunt occlusion for patients with hydrocephalus

A. East[1], T. Anor[2], D. Singh[1], M. Brophy[1], G. Eberl[1], P. J. Anand[1], M. Hammed[2], Z. Moses[2], J. R. Madsen[2]
[1]Alcyone Lifesciences Inc., Lowell, MA, USA; [2]Neurosurgical Department, Boston Children's Hospital, Boston, MA, USA
**Correspondence:** P. J. Anand
*Fluids and Barriers of the CNS* 2018, **15(Suppl 1)**:A36

**Introduction:** Ventricular Catheter (VC) occlusion is the predominant cause of shunt-malfunction, and a major unsolved problem. Clinical experience suggests that some occluded VCs can be unblocked by carefully "flushing" them with saline during surgery, and restoring cerebrospinal fluid flow. We sought to design a non-invasive retrograde flushing device, and a unique VC with the ability to open a new drainage hole. The goal of this device is to either flush out the proximal occlusions, or open a new hole in the VC to resume shunt flow.

**Methods:** We first quantified occluded-VC flushing volumes and pressures using a syringe attached to an in-line pressure sensor in patients undergoing shunt-revision. Using the data, the Flusher and VC was developed. During following investigations, The Flusher was primed with sterile saline, then attached to an in-line pressure sensor connected to an implanted occluded-VC. The Flusher dome was pressed and a controlled retrograde pulse of fluid was delivered to the proximal tip of the VC. Flow and pressure data were collected, the patient then underwent routine shunt revision.

**Results:** The Flusher successfully increased flow through 2 out of 4 occluded-VCs. In the VCs that did not resume flow, the pressure data indicated that the new drainage hole would have been opened had it been the new VC design.

**Conclusion:** The Flusher demonstrated its ability to flush and increase flow in a non-flowing VC. The pressure measurements indicated that the new drainage hole would have opened had it been the new VC design.

## A37

### Deterioration in gait velocity while waiting for shunt surgery for INPH

M. Ekblom[1], K. Cesarini[2], A. Gustafsson[3], J. Virhammar[1]
[1]Department of Neuroscience, Neurology, Uppsala University Hospital, Uppsala, Sweden; [2]Department of Neuroscience, Neurosurgery, Uppsala University, Uppsala, Sweden; [3]Department of Public Health and Caring Sciences, Uppsala University, Uppsala, Sweden
**Correspondence:** M. Ekblom
*Fluids and Barriers of the CNS* 2018, **15(Suppl 1)**:A37

**Introduction:** Waiting time for shunt surgery at our department for patients with idiopathic normal pressure hydrocephalus (iNPH) can be up to 6 months. Andrén et al. reported that patients with iNPH deteriorate over time and that the deterioration is only partially reversible after shunt surgery. The purpose of this study was to investigate if the previously reported deterioration in clinical symptoms over time is applicable also in our patients.

**Methods:** Gait velocity in 37 patients with iNPH was measured, using a 10-m walk test in self-chosen speed, at baseline, preoperatively (the day before surgery) and 3 months postoperatively. Median time between baseline and preoperative investigation was 6 months and between preoperative and postoperative investigation 3 months.

**Results:** There was a significant deterioration in gait velocity between baseline and preoperative investigation from 0.66 m/s (95% CI 0.59–0.74) to 0.57 m/s (95% CI 0.49–0.65), $p < 0.01$. Gait velocity increased from preoperative to postoperative investigation 0.71 m/s (95% CI 0.62–0.81), $p < 0.001$. There was no significant difference between baseline and postoperative investigation.

**Conclusions:** Gait function deteriorates over time in patients with iNPH. If waiting time for surgery for iNPH is long, progress of lost function may not reverse after shunt surgery. An assessment of NPH symptoms should be performed immediately preoperative for an accurate interpretation of postoperative improvement.

## A38

### CSF dynamic disturbance scale: summarising identified INPH pathophysiological fluid dynamic parameters into a single score

A. Eklund, S. Qvarlander, N. Sundström, L. O. Koskinen, O. J. Malm
Department of Radiation Sciences, Department of Clinical Neuroscience, Umeå University, Umeå, Sweden
**Correspondence:** A. Eklund
*Fluids and Barriers of the CNS* 2018, **15(Suppl 1)**:A38

**Introduction:** The shunt is accepted as an effective treatment for INPH and the shunt explicitly modifies the CSF dynamic. High Rout, high ICP amplitudes and elevated ICP, have shown good positive predictive values to select patients for shunt surgery, but not for exclusion of a patient from shunting. This indicates that the CSF dynamic disturbance is important, but that it is too complex to make single parameters reliable in patient selection for surgery. To make a holistic approach that includes multiple parameters we propose a CSF Dynamic Disturbance Scale (CSFDDS) that reflects the degree of disturbance of the system.

**Methods:** We analysed forty-seven patients with suspected INPH that were successfully investigated with an infusion protocol using the CELDA® infusion apparatus. The CSFDDS was defined based on Rout (0–3p), mean ICP (0–2p), ICP amplitude (0–3p) and ICP in sitting position (0–2p). Possible range 0–10p.

**Results:** In this preliminary analysis CSFDDS varied between 0 and 9 with a mean score of $3.6 \pm 2.3$. Twenty-three patients were selected for shunting mean CSFDDS $= 4.7 \pm 2.3$ compared to $2.6 \pm 1.8$ for the non-shunted group ($p = 0.001$ for the difference).

**Discussion:** Few patients were in the upper interval of the CSFDDS, indicating that the thresholds should be lowered. The scale provided a summary of the degree of CSF dynamic disturbance, and it has potential to provide the clinician with an easily interpreted number to support the diagnosis and selection for shunting. Possible CSF dynamic parameters to add to the scale are compliance, aqueductal CSF flow and potential to reduce the ICP pulse-amplitude with shunting.

## A39

### Withdrawn

*Fluids and Barriers of the CNS* 2018, **15(Suppl 1)**:A39

## A40

### Withdrawn

*Fluids and Barriers of the CNS* 2018, **15(Suppl 1)**:A40

## A41

### Endoscopic third ventriculostomy (ETV) in mechanically malfunctioning shunts in non-communicating hydrocephalic cases shunted below 1 year of age

O. Sorour, H. El Shafei, A. Alselisly, A. Elsaid, E. El Refaie
Department of Neurosurgery, Kasr Al Ainy School of Medicine, Cairo, Egypt
**Correspondence:** H. El Shafei
*Fluids and Barriers of the CNS* 2018, **15(Suppl 1)**:A41

**Introduction:** Extra cranial CSF shunts are more effective for treating non-communicating hydrocephalus in patients below the age of 1 year due to high failure rates of Endoscopic third ventriculostomy (ETV). This study determine the success rate of ETV in patients older than 1 year presenting with mechanical shunt malfunction who were shunted below 1 year of age, and evaluate different factors which determine the success rate.

**Methods:** Thirty patients, from July 2015 to October 2016, 16 M: 14 F, aged 1–25 years (mean 9.1 years) Grouped A 1–2 years (n4), B 2–8 years (n13) and C older than 8 (n13), were clinically and radiologically assessed pre-ETV and post ETV immediately and at 1, 2 and 4 months for hydrocephalus and patency of the ventriculostomy.

**Results:** Twenty one cases (70%) benefited from the ETV; 50% (n2) of Group A, 54% (n7) of Group B and 92% (nn12) of Group C. The mean age of successful ETV was 11 years and the failed ETV was 4.7 years. Shunted period ranged 9 months to 24 years and the mean period in successful ETV was 10 years and in failed ETV was 3.5 years. The shunt was removed in 16 case (13 success 53%) and legated and left in 2 (1 success 50%) and left untouched in 12 (7 success 30%). The mean follow up period for successful ETV was 10.5 months.

**Conclusions:** Endoscopic third ventriculostomy is a safe and effective procedure in treating hydrocephalus in patients older than 1 year presenting with mechanical shunt malfunction, in whom shunt was implanted below the age of 1 year due to non-communicating hydrocephalus.

## A42

### Optic pathway degeneration in patients with idiopathic normal pressure hydrochephalus (INPH)?

A. Eleftheriou[1], Y. Huang-Link[1], M. Fredrikson[2], F. Lundin[1]
[1]Division of Neurology, Department of Clinical and Experimental Medicine, Linköping University Hospital, Linköping, Sweden;
[2]Department of Occupational and Environmental Medicine, Linköping

Academic Research Center (LARC), Linköping University, Linköping, Sweden
**Correspondence:** A. Eleftheriou
*Fluids and Barriers of the CNS* 2018, **15(Suppl 1)**:A42

**Introduction:** Neuro-ophthalmological manifestation, such as transient visual cognitive symptoms but also optic atrophy, visual fields defects and papilledema in patients with iNPH has been described in very few cases. Mechanism underline visual disturbances may be related to abnormal CSF dynamics but evidence is lacking. Magnetic resonance imaging (MRI) and optical coherence tomography (OCT) enable us to study optic pathway within the brain and in the eye fundus. We aim to explore visual disturbances in patients with iNPH.
**Methods:** Fourteen consecutive patients fulfilled the criteria of iNPH according to international guidelines, including 5 females and 9 males, aged 64–84 years. All patients underwent neurologic, neuro-ophthalmological examination, CSF, cognitive screening and physiotherapeutic evaluation. MRI, CSF tap test via lumbar puncture (LP) with CSF removal of 44 ml (35–50) was performed in all patients. Before and after CSF removal, OCT, motor function was tested.
**Results:** Mean value of CSF pressure was $16 \pm 3$ respective $2 \pm 2$ cm $H_2O$ before and after LP with CSF removal. Twelve of 14 patients showed significant reduced visus $0{,}5 \pm 0{,}3$ (0.2–1). MRI measurements showed mean values of Evans ratio ($0.36 \pm 0.03$), corpus callosal angle ($73 \pm 11$) and width of third ventricle ($12 \pm 2$ mm). Patients had significant reduced retinal ganglion cell layers (GCL) thickness ($72 \pm 10$) compared to healthy control (HCs) ($80 \pm 5$ microns) ($p < 0{,}001$), but no changes were observed before and after tap test.
**Conclusion:** This is the first study raising question about neurodegeneration in the optic pathway in iNPH patients.

## A43

### Identifying and parcellating the subarachnoid space

M. Shao[1], A. Carass[1], X. Li[1], J. Shin[2], S. Roy[3], J. L. Prince[1], A. M. Blitz[2], L. M. Ellingsen[1,4]
[1]Department of Electrical and Computer Engineering, Johns Hopkins University, Baltimore, MD, USA; [2]Department of Radiology and Radiological Science, Johns Hopkins Hospital, Baltimore, MD, USA; [3]CNRM, The Henry M. Jackson Foundation for the Advancement of Military Medicine, Bethesda, MD, USA; [4]Department of Electrical and Computer Engineering, University of Iceland, Reykjavik, Iceland
**Correspondence:** L. M. Ellingsen
*Fluids and Barriers of the CNS* 2018, **15(Suppl 1)**:A43

**Introduction:** Volumetric evaluation of the subarachnoid space is important in various cerebrospinal fluid disorders. The task is complicated and tedious for manual raters and prone to rater bias. An additional complexity is the identification of an appropriate cortical label to parcellate the volumetric information into clinically relevant quantities. Here we present a novel approach to automatically identify and parcellate the subarachnoid space.
**Methods:** We present a deformable model based approach that propagates an estimate of the pial surface outwards using image based forces from T1- and T2-weighted magnetic resonance (MR) acquisitions. The deformable surface also carries with it one of the 100+ Neuromorphometrics' labels from the cortical mantel. Using this approach, we are able to construct thicknes s maps of the subarachnoid space with detailed cortical labels.
**Results:** We present subarachnoid thickness maps on a cohort comprising healthy controls and normal pressure hydrocephalus (NPH) patients. The thickness map in conjunction with the automated parcellation of the subarachnoid space demonstrates the power of the method to identify patient specific regions of change.
**Conclusions:** Developing methods for automated neuroimage processing is an important step for expediting the clinical evaluation of patients. Clinical observations, such as disproportionally enlarged subarachnoid space in NPH, are often challenging to identify by looking at stacks of two dimensional MR images. Our method enables quantification of the subarachnoid space that could help clinicians identify specific regions of enlargements and associations with possible bottlenecks in the path of CSF flow.

## A44

### Intracranial pressure in papilloedema

J. P. Funnell, C. L. Craven, L. D'Antona, A. Chari, S. Thompson, L. Thorne, L. D. Watkins, A. K. Toma
Victor Horsley Department of Neurosurgery, National Hospital for Neurology and Neurosurgery, Queen Square, London, UK
**Correspondence:** J. P. Funnell
*Fluids and Barriers of the CNS* 2018, **15(Suppl 1)**:A44

**Introduction:** There remains controversy over normal values of intracranial pressure (ICP). Papilloedema is a clinical manifestation of chronically raised ICP, often seen in idiopathic intracranial hypertension (IIH). Therefore, papilloedema may be useful for establishing normal ICP thresholds. Here we aim to compare ICP values in IIH patients who developed papilloedema and those who did not.
**Methods:** Single centre cohort of IIH patients (2006–2016) who underwent 24-h ICP monitoring (ICPM) and ophthalmology assessments, prior to intervention. Papilloedema was graded according to the Frisén scale. An un-paired t-test compared 24-h ICPM between papilloedema and no-papilloedema groups. Fisher's exact test was used to determine predictive value of ICP.
**Results:** 36 patients with IIH (35 F: 1 M), mean age $32.5 \pm 9.49$ years. Of the 25 patients with papilloedema, 6 had early disc abnormalities, 12 moderate papilloedema, 3 severe papilloedema and 4 had no grading recorded. 11 patients had no papilloedema. Patients with papilloedema had a mean median 24-h ICP of $10.4 \pm 5.32$ mmHg, significantly higher than the group without papilloedema ($6.31 \pm 3.30$ mmHg) ($p < 0.05$). Using 24-h median ICP of 10 mmHg as a minimum cut-off predictive value, specificity = 91%, sensitivity = 48%, PPV = 92% and NPV = 44%.
**Conclusions:** Our results suggest that a 24-h ICP of 10 mmHg or more is a good predictor for papilloedema and reflects a pathological threshold. The range varied widely suggesting papilloedema can occur at even lower pressures. These results are consistent with emerging evidence suggest that pathologically 'high' ICP is much lower than previously quoted.

## A45

### Tinnitus in idiopathic intracranial hypertension

J. P. Funnell, C. L. Craven, L. D'Antona, A. Chari, S. Thompson, L. Thorne, A. K. Toma, L. D. Watkins
Victor Horsley Department of Neurosurgery, National Hospital for Neurology and Neurosurgery, Queen Square, London, UK
**Correspondence:** J. P. Funnell
*Fluids and Barriers of the CNS* 2018, **15(Suppl 1)**:A45

**Introduction:** Tinnitus is a symptom commonly associated with idiopathic intracranial hypertension (IIH) and can impair quality of life. We aim to (1) determine if tinnitus correlates with intra-cranial pressure (ICP) and (2) determine symptom response after dural venous sinus stenting (DVSS) or CSF diversion with a shunt.
**Methods:** Single centre cohort of IIH patients (2006–2016) who underwent 24-h ICP monitoring (ICPM). An un-paired t-test compared ICP and pulse amplitude (PA) values between (1) patients with and without tinnitus, and (2) patients with pulsatile tinnitus (PT) vs. subjective tinnitus (ST).
**Results:** We identified 59 patients with IIH (56F: 3 M), mean age $39.5 \pm 11.1$ years, 14 of whom suffered from tinnitus. Of these 14, seven reported PT and seven reported 'ringing', 'humming' or 'clicking' sounds (ST). Patients with tinnitus had a mean 24-h ICP and PA of $9.09 \pm 5.25$ and $6.05 \pm 1.07$ mmHg respectively. There was no significant difference in ICP nor PA between patients with tinnitus and without ($p = 0.84$), nor between PT vs. ST ($p = 0.20$). All 7 patients with PT showed symptom improvement or resolution after DVSS ($n = 4$), secondary DVSS ($n = 2$) or Shunting ($n = 1$). Of the 7 with ST, only 1 improved post intervention (DVSS), despite 2 patients having shunts and 5 having DVSS.
**Conclusions:** DVSS appears to be an effective management option for IIH patients with a clear history of pulsatile tinnitus. However, subjective tinnitus was more persistent and did not respond well to either DVSS or CSF diversion.

## A46

### Can upper limb and cognitive outcome measures identify change in patients undergoing a lumbar puncture tap test with idiopathic normal pressure hydrocephalus (INPH)?

R. Gallagher[1,2], J. Marquez[2,3], P. Osmotherly[2]
[1]Physiotherapy Department, John Hunter Hospital, Newcastle, Australia; [2]School of Health Sciences, The University of Newcastle, Newcastle, Australia; [3]Hunter Medical Research Institute, Newcastle, Australia
**Correspondence:** R. Gallagher
*Fluids and Barriers of the CNS* 2018, **15(Suppl 1)**:A46

**Introduction:** The lumbar puncture tap test (TT) is regularly utilised to identify shunt responsive iNPH. Testing regimes aim to identify change in function but vary significantly. This study aimed to determine if a battery of upper limb and cognitive outcome measures can identify change in iNPH patients undergoing a TT.
**Methods:** Prospective cohort study of 74 patients undergoing a TT diagnosed with iNPH. Patients performed the Timed up and go cognition test (TUG-C), 9-hole peg test, and Montreal Cognitive Assessment (MoCA) before and after a TT. A Neurologist determined response status, patients who improved were labelled as responders. Sign-rank tests were used to analyse between groups differences.
**Results:** Forty patients were categorised as responders, 34 non-responders. For responders, the median change in the TUG-C (− 6.02 s $p < 0.01$) and MoCA (0.62 points $p = 0.02$) was significant. Only executive function and orientation sub scores of the MoCA showed significant change (1 point each, $p = 0.03$). The median 9 hole peg test change (4.33 s $p = 0.14$) was not significant. For non-responders changes of 0.22 points for the MoCA ($p = 0.51$), 0.3 s for TUG-C ($p = 0.63$) and 2.58 s for the 9 hole peg test ($p = 0.51$) were not significant.
**Conclusions:** The TUG-C can identify change following a TT and should be considered for use. Change on the MoCA, of less than 1 point, cannot be considered clinically significant. Further investigation is required regarding the ability of sub scores of the MoCA to identify change. The 9-hole peg test cannot identify change and cannot be recommended.

## A47

### Can gait and balance measures identify individuals who respond to a lumbar puncture tap test in patients with idiopathic normal pressure hydrocephalus (INPH)?

R. Gallagher[1,2], J. Marquez[2,3], P. Osmotherly[2]
[1]Physiotherapy Department, John Hunter Hospital, Newcastle, Australia; [2]School of Health Sciences, The University of Newcastle, Newcastle, Australia; [3]Hunter Medical Research Institute, Newcastle, Australia
**Correspondence:** R. Gallagher
*Fluids and Barriers of the CNS* 2018, **15(Suppl 1)**:A47

**Introduction:** The lumbar puncture tap test (TT) is a common test to identify shunt responsive iNPH. Varying testing regimes are used to identify change but specific tests are not supported by evidence. This study sought to determine if a battery of gait and balance outcome measures can identify change for patients with iNPH undergoing a TT.
**Methods:** A prospective cohort study of 74 patients undergoing a TT. Patients were assessed before and after a TT using the: 10 m walk test, Timed up and go (TUG), Tinetti assessment, and Berg Balance Scale (BBS). Patients deemed to have improved by a Neurologist were labelled as responders. Between group differences were analysed with sign-rank tests. Change scores were compared to established minimal detectable changes (MDC's) for these tests. Sample size calculations were based on established MDC's for the TUG.
**Results:** Forty patients responded, 34 were non-responders. For responders, significant change was identified for the TUG (3.98 s $p < 0.01$), Tinetti (3.88 points $p < 0.01$), Berg (5.29 points $p < 0.01$) and 10 m walk (0.08 m/s $p < 0.01$). For non-responders significant change was identified for the Tinetti, (0.91 points $p = 0.01$) and Berg, (2.06 points $p < 0.01$). For responders, the 10 m walk change fell within established MDC's, for non-responders, all change scores fell within established MDC's.
**Conclusions:** This research provides the first evidence to support specific tests which identify change following a TT in iNPH. The TUG, BBS and Tinetti can identify change. The 10 m walk test does not appear to be sensitive to identify change with change scores below established MDC's.

## A48

### Is the sagittal sinus involved in idiopathic normal pressure hydrocephalus (INPH). Analysis of MRI CSF flow studies in patients undergoing a CSF tap test

R. Gallagher[1,3], G. A. Bateman[2,3], J. Marquez[3], P. Osmotherly[3]
[1]Physiotherapy Department, John Hunter Hospital, Newcastle, Australia; [2]Medical Imaging, John Hunter Hospital, Newcastle, Australia; [3]Priority Research Area for Stroke and Brain Injury, The University of Newcastle, Newcastle, Australia
**Correspondence:** R. Gallagher
*Fluids and Barriers of the CNS* 2018, **15(Suppl 1)**:A48

**Introduction:** The lumbar puncture tap test (TT), routinely used to identify shunt responsive iNPH, is limited by low negative predictor values. MRI CSF flow studies are often utilised in conjunction with a TT to improve identification of shunt responsive iNPH. This study aimed to identify what markers on MRI CSF flow studies which can identify change in gait from a TT.
**Methods:** Prospective cohort study of 42 patients undergoing a TT and MRI CSF flow studies. Patients completed a timed up and go (TUG) test before and after a TT. The minimum detectable change (MDC) score for the TUG was used as the cut off value. Sign-rank tests were used to evaluate between groups differences.
**Results:** 17 patients improved on the TUG, 27 did not improve. Significant between group differences were found for (no improvement vs. improved): sagittal sinus circumference (26.60 mm vs. 24.89 mm $p < 0.01$), sagittal sinus area (41.6 $mm^2$ vs. 34.4 $mm^2$ $p < 0.01$), sagittal sinus stroke volume (225 µl vs. 172 µl $p = 0.04$) and superior sagittal sinus flow percentage (47.77% vs. 38.83% $p = 0.03$). No differences were present for aqueduct stroke volume (140 µl vs. 140 µl $p = 0.57$), aqueduct net flow (0.002 ml/s vs. 0.08 ml/s $p = 0.21$), arterial inflow (8.29 ml/s vs. 9.31 ml/s $p = 0.21$) or compliance ratio (7.94 vs. 12.71 $p = 0.45$)
**Conclusions:** A link between improvement in gait symptoms and sagittal sinus measurements indicates that the sagittal sinus may play a role in the manifestation of symptoms in iNPH. This may also have a role in diagnosis of iNPH. Further research is required to confirm the significance of these findings.

## A49

### Epidemiological characterisation of normal pressure hydrocephalus

C. Garcia-Baena, J. S. Davidson, M. F. Cárdenas, J. Mendoza, F. Hakim, J. F. Ramón
Neurosurgery Section, Department of Surgery, Fundación Santa Fe de Bogotá, Bogotá, Colombia
**Correspondence:** C. Garcia-Baena
*Fluids and Barriers of the CNS* 2018, **15(Suppl 1)**:A49

**Introduction:** Epidemiological data on Normal Pressure Hydrocephalus is limited, because for the most part, knowledge is derived from indirect estimates and studies not designed specifically for this purpose. From this, incidence and prevalence data representative of the Latin American population is near null, and inexistent for our Country, so our main objective was to calculate the prevalence of NPH in Colombia.
**Methods:** Prevalence was calculated for 7 of the Country's cities, chosen based on the number of Neurosurgical specialists in each. We used a nationwide database from the health regulation institution (SISPRO) to extract the number of cases, from 2009 to 2015, and population census information from the relevant institution (DANE). Diagnosis was established using the ICD-10 code for NPH. Overall prevalence and point prevalence for each year was calculated, standardising by age, sex and income based on health insurance regimen.
**Results:** Total prevalence was 0.01%. It increased from 2009 to 2011 but was relatively constant in the ensuing years. Prevalence was 0.0104% for women and 0.0127% for men, and increased with age for both men and women. 73.03% of cases were seen in the middle-to-high-income group of patients. Most were in men, even though the ratio to women varied widely

**Conclusions:** We believe NPH prevalence is underestimated and plan to design a programme to educate upon the usage of the specific diagnostic ICD-10 code, with subsequent revaluation, as we have reason to believe its use is not generalised amongst the members of the specialty.

## A50

### Cerebrospinal fluid closing pressure-guided tap test in the diagnosis of normal pressure hydrocephalus

D. Gómez, M. F. Cárdenas, J. Mendoza, C. Garcia-Baena, F. Hakim, J. F. Ramón
Neurosurgery Section, Department of Surgery, Fundación Santa Fe de Bogotá, Colombia
**Correspondence:** D. Gómez
*Fluids and Barriers of the CNS* 2018, **15(Suppl 1)**:A50

**Introduction:** The Tap test has been a fundamental pillar in the diagnosis of Normal Pressure Hydrocephalus from its first description as an independent entity, and is based on the existent relationship between a positive test result and subsequent clinical improvement in the three principal manifestations of the pathology; The latter, an aspect that has been objectivised in distinct manners with the passing of time. It has been stated that the volume of CSF fluid to be extracted during the test should be between 30 and 50 cc, even though there is no explanation as to why this should be the quantity. Therefore, in our institution, we have established a practise whereby the volume of fluid extracted is based on a CSF closing pressure of 0 cm $H_2O$. We are seeking to describe our experience and propose that this parameter be set as a standard in Tap test performance.

**Methods:** A retrospective descriptive multivariate analysis, using data obtained between 2005 and 2015 from our institution, is to be carried out.

**Results:** We have seen that an augmented Tap test sensitivity in comparison to what has been previously reported is appreciated.

**Conclusions:** Our experience suggests that the drainage of CSF based on a pressure of 0 cm $H_2O$ at the time of closure increases the likelihood of a positive result for NPH. Drainage parameters can be standardised as well as individualised, by a closing pressure-guided Tap test.

## A51

### Sleep breathing disorder prevalence in a population of patients suspected of idiopathic normal pressure hydrocephalus

S. Goutagny[1], R. Wanono[2,3], A. Robinot[2,4], S. Mrejen[1], A. Stosse[1], Y. Sogoba[1,5], M. P. d'Ortho[2,3,4], P. Decq[1,3]
[1]Neurosurgery, Hôpital Beaujon, Assistance Publique-Hôpitaux de Paris, Paris, France; [2]Service d'Explorations Fonctionnelles, DHU FIRE, Hôpital Bichat, Assistance Publique-Hôpitaux de Paris, Paris, France; [3]Université Denis Diderot, Sorbonne Paris Cité, Paris, France; [4]DHU PROTECT, INSERM U1141, Université Paris Diderot, Sorbonne Paris Cité, Paris, France; [5]Neurosurgery, CHU Gabriel Touré, BP 267, Bamako, Mali
**Correspondence:** S. Goutagny
*Fluids and Barriers of the CNS* 2018, **15(Suppl 1)**:A51

**Introduction:** Both sleep apnea syndrome (SAS) and normal pressure hydrocephalus (NPH) are responsible for falls, cognitive impairment, and urinary urgencies in elderly. Association of these conditions has been described. Our goal is to evaluate the prevalence of SAS in patients suspected of NPH.

**Methods:** Tertiary reference center for hydrocephalus. Since 08/2015 we added to NPH evaluation one night breathing recording using a portable device (Apnealink@). Presenting symptoms were gait disorders and/or urinary incontinence and/or cognitive dysfunction, and ventriculomegaly (Evans' index > 0.3). Sleeping respiratory events were quoted using the 2012 American Academy of Sleep Medicine criteria. Clinical evaluation was based on the European iNPH scale. Decision whether shunting or not was based on symptoms, MRI findings, lumbar tap test, and lumbar CSF infusion test. When appropriate, patients were offered ventriculo-atrial shunting using a programmable valve. Improvement was defined as an increase $\geq 5$ on iNPH scale.

**Results:** Fifty-six patients were included (28 women, 27 men) with a median age of 74.9 years (± 8.4). The mean MMSE was 26 (range 14–30). The median body mass index was 27 (± 4.5) kg/m$^2$. NPH was considered unlikely in 20 patients (36%); 35 patients were eligible for shunting and 24 patients (64%) have been operated on. Clinical improvement after shunting was noticed in 20 patients (83%). SAS (apnea–hypopnea index (AHI) > 5/h) was recorded in 39 patients (71%), severe (AHI > 30) in 14 (25%). Hypoxemia (> 10% of recording with SpO2 < 90%) was evidenced in 38% of patients.

**Conclusions:** The prevalence of SAS is high among patients investigated for possible NPH.

## A52

### Patient's expectations of shunt treatment and their role in the rehabilitation process, an interview study

A. Gus tafsson[1], J. Virhammar[2], M. Ekblom[2], K. Cesarini[3], B. Jakobsson Larsson[2]
[1]Department of Public Health and Caring Sciences, Uppsala University, Uppsala, Sweden; [2]Department of Neuroscience, Neurology, Uppsala University Hospital, Uppsala, Sweden; [3]Department of Neuroscience, Neurosurgery, Uppsala University Hospital, Uppsala, Sweden
**Correspondence:** A. Gus tafsson
*Fluids and Barriers of the CNS* 2018, **15(Suppl 1)**:A52

**Introduction:** It has been observed that some patients with idiopathic normal pressure hydrocephalus (iNPH) experience that the post-operative result is not what they expected and they experience deterioration at postoperative follow-up even though standardized tests show improvement. It has also been observed that rehabilitation possibilities for patients varies, ranging from structured rehabilitation periods, e.g. at geriatric rehabilitation units to basically no rehabilitation at all. The aim of this study was to describe the expectations patients with iNPH have on shunt treatment and how they view their own role in the rehabilitation process.

**Methods:** A qualitative, descriptive design with manifest and inductive approach. Seven patients with iNPH were interviewed preoperatively using a semi-structured approach.

**Results:** The informant's main expectations on shunt treatment, concerned improvements in gait, continence, cognition and a desire to become more autonomous. The informants expressed a personal responsibility for the rehabilitation process and suggested what they could contribute with in the process but also different obstacles for performing physical activities were described.

**Conclusions:** The patient's expectations of outcome after shunt treatment should be discussed with the patient and their relatives prior to shunt surgery. The rehabilitation process needs to be discussed with the patient both pre- and postoperative in order to optimize outcome.

## A53

### Ventriculo-atrial shunt insertion using transesophageal echo: initial experience

M. G. Hamilton[1], A. M. Isaacs[1], G. Urbaneja[1], D. Krahn[2], K. Rogan[2], A. Walker[2], C. Prusinkiewicz[2], H. Hurdle[2]
[1]Department of Clinical Neurosciences, University of Calgary, Calgary, Canada; [2]Department of Clinical Anaesthesia, University of Calgary, Calgary, Canada
**Correspondence:** M. G. Hamilton
*Fluids and Barriers of the CNS* 2018, **15(Suppl 1)**:A53

**Introduction:** The main treatment of hydrocephalus is surgical diversion of CSF from the cerebral ventricles, commonly to the peritoneal cavity (ventriculo-peritoneal (VP) shunt) using a shunt. One of the major challenges in managing hydrocephalus stems from the fact that 40–60% of shunts fail within the first 2 years. In adults, 80–90% of those failures are due to peritoneal catheter malfunction. In many cases, significant intra-abdominal adhesions or scarring contributes to shunt failure. An alternative approach is to divert CSF flow to the right atrium (i.e. a ventriculo-atrial (VA) shunt).

**Methods:** A prospective adult patient shunt outcome registry was established in January 2012 and concurrent patients enrolled. Distal shunt malfunction was confirmed by: (1) clinical deterioration in symptom control, and (2) abnormal structural or functional findings in CT/MRI scan, nuclear medicine shunt study or shunt X-ray. In April 2015, a laparoscopy-guided approach to place the distal catheter under direct visualization over the dome of the liver was initiated to attempt a reduction in distal catheter failure rates. All laparoscopically treated patients were prospectively graded regarding severity of intra-abdominal adhesions, and considered for conversion to a VA shunt after shunt failure in the presence of previously noted significant intra-abdominal adhesions.
**Results:** We have treated 222 adult patients for hydrocephalus of different etiologies with 177 patients having a new shunt insertion (VPSI) and 51 patients presenting with an established VP shunt and in need of a shunt revision (VPSR). A total of 135 patients underwent laparoscopic-guided distal catheter placement (88 VPSI: 45 VPSR). 11 patients underwent VP to VA shunt conversion after VP shunt failures associated with significant intraabdominal adhesions. The first seven patients had surgery with fluoroscopic guidance. Patient number 7 had incorrect placement of the atrial catheter in the superior vena cava (SVC) with shunt malfunction. Transesophageal ECHO was used in the VA shunt revision of patient number 7 and the remaining three patients, all with reliable placement of the tip of the catheter 1.5–2.0 cm into the right atrium. There were no surgical complications in the VA shunt patients.
**Conclusions:** VA shunts are a viable alternative to VP shunts for the treatment of adult patients with hydrocephalus, especially in patients with significant abdominal adhesions which affect VP shunt viability. Transesophageal ECHO is an eloquent and accurate technique that is an alternative to use of fluoroscopy to confirm position of the atrial catheter.

## A54

### Development and application of a surgical site infection prevention bundle for shunt-related insertions and revisions

M. G. Hamilton[1], A. Fung[2], D. Lam-Li[2], A. Isaacs[1], J. Conly[2]
[1]Department of Clinical Neurosciences, University of Calgary, Calgary, Canada; [2]Department of Medicine, University of Calgary, Calgary, Canada
**Correspondence:** M. G. Hamilton
*Fluids and Barriers of the CNS* 2018, **15(Suppl 1)**:A54

**Introduction:** Insertion of cerebrospinal fluid (CSF) shunts are often necessary for hydrocephalus. Shunt-related surgical site infections (SRSSI) may result in significant morbidity including shunt malfunction, cognitive impairment, and require antibiotic therapy and surgical replacement. Rates of SRSSIs of 4.85 and 3.24% in children and adults, respectively, have been reported in the Canadian Nosocomial Infection Surveillance Program (CNISP) in 2013. We developed a CSF shunt SSI Prevention Bundle (SSIPB) with a 9-point checklist as a quality improvement initiative and evaluated it post-implementation. No antibiotic-impregnated catheters were used.
**Methods:** A prospective surveillance system for SRSSI was designed based upon the CNISP protocol. Inclusion criteria included insertion or revision of any ventriculoperitoneal (VP), ventriculoatrial (VA), or lumboperitoneal (LP) shunts and exclusion criteria included patients with transcutaneous or external shunting devices or non-shunting devices, patients whose CSF was culture-positive at the time of shunt placement and if the surgery occurred > 12 months before the infection was identified. A shunt SSI was defined as having a microbe isolated from the CSF with a shunt in situ and associated with at least one of: fever ($\geq$ 38 °C); neurological signs or symptoms; abdominal signs or symptoms or; signs or symptoms of shunt malfunction/obstruction. Denominator data was provided via an Operating Room database. Data quality measures included discussing difficult cases with an ID physician, the surveillance team and a neurosurgeon. A SSIPB with a 9-point checklist addressing pre-, peri- and post-operative care was developed by Neurosurgery and Infection Prevention and Control (IPC) and then evaluated using an uncontrolled before–after design. Differences were assessed using a $x^2$ or Fisher exact test as appropriate.
**Results:** The implementation of the bundle occurred over a 24 month period to ensure a full culture change. Comparing the before-after periods of 2012—Q32015 vs. Q42015—total rates of SRSSI of 17/431 (3.94%) vs. 1/160 (0.63%) were noted ($p = 0.055$, two tailed). Insertion rate SRSSIs for 2012—Q32015 vs. Q42015—2016 were 12/205 (5.9%) vs. 0/66 ($p = 0.04$, two-tailed). Checklist compliance was completed for all procedures which allows for secondary analysis and remediation.
**Conclusions:** We observed a 6.3-fold reduction overall and a statistically significant decrease of SRSSIs for primary shunt insertions post-implementation of a shunt SSIPB without the use of antibiotic-impregnated catheters. Achieving compliance with the OR checklist was challenging; however, it has achieved significant improvements in an operative setting where SSIs are associated with high morbidity.

## A55

### Long term outcome of surgically treated idiopathic normal pressure hydrocephalus and the factors related to the poor outcomes

T. Hashiba, N. Yamamura, M. Miyata, H. Isozaki, I Lee, T. Kamei, R. Iwata, J. Takeda, K. Yoshimura, M. Nonaka, A. Asai
Department of Neurosurgery, Kansai Medical University, Hirakata, Japan
**Correspondence:** T. Hashiba
*Fluids and Barriers of the CNS* 2018, **15(Suppl 1)**:A55

**Introduction:** DESH is a powerful image marker in preoperative diagnosis of iNPH, and in the cases with DESH, postoperative improvement can be highly expected. On the other hand, their long term outcomes are not always good. Long-term outcomes were examined for definite i-NPH cases experienced by the authors.
**Methods:** Of the cases that showed postoperative improvement, 40 cases who had been pursued for more than 1 year were investigated.
**Results:** 2 cases were deliberately shunt removed, 4 cases died during the course, and 34 cases had continued to be shunted. The average follow-up periods of 34 patients with shunt continuation was 33.4 months. Of 34 patients, 19 cases maintained improvement, and 15 cases went backward to preoperative level or worsened. Investigation for clinical factors related to long-term outcome showed that the existence of comorbidities and termination of direct follow up were statistically significant. Main reasons for deterioration were occurrences of new problems due to exacerbations of comorbidities. Terminations of direct follow-up were mainly caused by deterioration of symptoms and entrance to the facility, but difficulty in going to the hospital due to the transfer of the doctor in charge was also one of the reasons.
**Conclusions:** Although the presence of DESH is related to improvement immediately after surgery, the existence of comorbidities is greatly related to deterioration of long-term performance. Also, termination of direct follow-up possibly may promote deterioration in performance. Therefore, if follow-up is interrupted according to the circumstances of the doctor, it is necessary to introduce to the doctor who understands i-NPH well.

## A56

### Withdrawn

*Fluids and Barriers of the CNS* 2018, **15(Suppl 1)**:A56

## A57

### Nonsymmetrical pulsatile pressure differences across an open cerebral aqueduct. A cause of ventriculomegaly?

P. Holmlund[1], S. Qvarlander[1], J. Malm[2], A. Eklund[1]
[1]Department of Radiation Sciences, Biomedical Engineering, Umeå University, Umeå, Sweden; [2]Department of Pharmacology and Clinical Neuroscience, Umeå University Hospital, Umeå, Sweden
**Correspondence:** P. Holmlund
*Fluids and Barriers of the CNS* 2018, **15(Suppl 1)**:A57

**Introduction:** INPH is characterized by unexplained ventriculomegaly and often with increased pulsatility. We hypothesize that the cardiac-related pulsatile flow over the cerebral aqueduct (CA), with fast systolic outflow and slow diastolic inflow, can generate net pressure effects that could source ventriculomegaly in INPH. Our hypothesis predicts a cardiac cycle averaged net pressure difference ($P_{DIFF}$) over the CA, with higher average pressure in the lateral and third ventricles. The aim of this study was to investigate these potential pressure differences arising from the nonsymmetrical CA flow in INPH.
**Methods:** The hypothesis is tested using (1) A CA bench model and (2) Pressure estimations based on MRI-imaging of 25 INPH patients.

For (1) typical pulsatile aqueductal flows found in healthy and INPH patients were applied to the bench aqueduct model and $P_{DIFF}$ was measured. For (2) we will make use of computational fluid dynamics (CFD) to calculate the pressure distributions (over the CA), based on structural MRI (area) and 2D-PCMRI (velocity) data.

**Results:** The bench-test/aqueductal model verified a net $P_{DIFF}$ over the cardiac cycle, in the caudal direction. The effect was magnified for flow in INPH compared to flow in healthy ($P_{DIFF}$: 0.18 vs. 0.04 mmHg, $p < 0.01$). The CFD-simulations, using the MRI-measurements, are currently under analysis and will be presented at the conference.

**Conclusions:** The experimental bench-test data supported that ventriculomegaly in INPH could be caused by pressure differences introduced during each heartbeat over the CA. Identifying the cause of the ventricular enlargement will have implications for diagnosis and selection of treatment.

## A58

### Intracranial pressure in upright body positions and the importance of venous collapse

P. Holmlund[1], A. Eklund[1], L. O. D. Koskinen[2], E. Johansson[2], N. Sundström[1], J. Malm[2], S. Qvarlander[1]
[1]Department of Radiation Sciences, Biomedical Engineering, Umeå University, Umeå, Sweden; [2]Department of Pharmacology and Clinical Neuroscience, Umeå University, Umeå, Sweden
**Correspondence:** P. Holmlund
*Fluids and Barriers of the CNS* 2018, **15(Suppl 1)**:A58

**Introduction:** In hydrocephalus an important pathophysiological factor is ICP. Its regulation is primarily understood and measured in the supine position, despite patients mostly being upright and shunt over-drainage being a common complication. To fill this gap we have suggested a model, based on Davson's equation for CSF absorption, where postural ICP changes depend on hydrostatic effects in the venous system and collapse of the internal jugular veins (IJVs). The aim of this study was to investigate this relationship by simultaneous invasive measurements of ICP, venous pressure and IJV collapse in healthy volunteers.

**Methods:** ICP (monitored via the lumbar route), central venous pressure (PICC-line) and IJV collapse (ultrasound) were assessed in eleven healthy volunteers (47 $\pm$ 10 years) in seven positions of upper-body tilt, going from supine to sitting. Hydrostatic distances were measured to adjust the pressures to the ICP reference point (auditory canal). Venous pressure and IJV collapse were inputs to the ICP prediction. Predicted and measured ICP were compared for each tilt-angle.

**Results:** The model accurately predicted the general behavior of ICP (mean difference for all tilt- angles: 0.8 $\pm$ 3.0 mmHg, $p > 0.21$ for each tilt-angle), although individual variations were observed.

**Conclusions:** The results support that postural ICP changes are governed by IJV collapse and venous hydrostatics. This new understanding of the ICP regulatory system in upright demands the exploration if its importance in diseases like INPH, and should be considered in the design of shunts. The results are also important for understanding the mechanisms behind head-elevation therapy of ICP in critically ill patients.

## A59

### Changes in endovascular venous sinus pressure gradient measurement before and after general anesthesia in idiopathic intracranial hypertension

A. El Mekabaty[1], E. Obusez[2], A. Gottschalk[3], C. Chung[4], M. G. Luciano[5], F. Hui[1]
[1]Division of Interventional Neuroradiology, The Johns Hopkins Hospital, Baltimore, MD, USA; [2]Department of Radiology, Cleveland Clinic, Cleveland, OH, USA; [3]Department of Anesthesiology and Critical Care Medicine, The Johns Hopkins Hospital, Baltimore, MD, USA; [4]Department of Surgery, Massachusetts General Hospital, Boston, MA, USA; [5]Department of Neurosurgery, The Johns Hopkins Hospital, Baltimore, MD, USA
**Correspondence:** F. Hui
*Fluids and Barriers of the CNS* 2018, **15(Suppl 1)**:A59

**Introduction:** Idiopathic intracranial hypertension (IIH) is a yet not fully understood disease, which is more commonly encountered in obese, young age women. The presence of venous hypertension is often implicated as the pathophysiological origin of IIH, which is postulated to arise as a squeal to intracranial venous stenosis or intrathoracic origin (such as obesity and elevated central venous hypertension). Intracranial venous stenosis has been described in up to 90% of IIH patients. Recently, a case series examined the effect of conscious sedation (CS) and general anesthesia (GA) on endovascular venous pressure gradient measurements. Their results suggest that there is a sizable difference between measurements taken under CS and those under GA, which has an effect on the selection process of IIH patients by either overestimating or underestimating the pressure gradient. In this report, we aim to examine effect of GA of endovascular pressure gradient measurements in IIH patients as well as the decision to go on with stenting.

**Methods:** We performed a retrospective chart review of all patients, who received endovascular transverse sinus stenting due to medical treatment refractory IIH between August 2013 and March 2017 in our institution was performed. Patients who received an endovascular venous pressure measurement during conscious sedation and under general anesthesia in the same setting were then identified. Chart analysis included: patients' demographics; anesthetic agents used in CS and GA; endovenous pressure measurements during CS, GA and after stent placement; stent type and size; and whether the decision to place the stent was revised based on the change in pressure measurements. The threshold for stenting was a pressure gradient across the transverse sinus stenosis of > 6 mm Hg.

**Results:** We identified 12 patients who received endovascular pressure gradient measurements under CS and GA. All patients (100%) were female. The mean age was 30.7 years (SD 8.8 and rage 15–42) and the mean BMI was 40.7 (SD 10.1, range 28–63.7). Anesthetic agents used during CS were; midazolam, fentanyl and propofol and during GA; propofol and vapor anesthetic (Sevoflurane in 6 patients, Desflurane in 4 patients, and Isoflurane in 2 patients). Mean pressure gradient under CS was 21.7 mmHg (SD 8.5) and 15.8 mmHg (SD 7.9) under GA. Eight (66%) patients showed a pressure gradient reduction after initiation of GA (average 10 mmHg, range 1–20), 2 (18%) patients showed an increase of pressure gradient under GA (2 and 9 mmHg) and 2 (18%) patients had a stable gradient. Post-stenting the pressure gradient was successfully reduced to an average of 2.1 mmHg (SD 2.6).

**Conclusions:** When performed in the same setting, endovascular pressure gradient across transverse stenosis in IIH patients performed under GA shows a sizable reduction (6 mmHg) compared with measurements taken under CS. Further studies are needed to examine the role anesthetic agents and different accompanying factors in regard to the optimal pressure gradient threshold for transverse sinus stenting in IIH patients.

## A60

### The copy number loss in the intron two of the sfmbt1 among finnish INPH-patients and families

J. Huovinen[1], V. Korhonen[1], A. M. Remes[2], S. Helisalmi[3], V. Leinonen[1]
[1]Institute of Clinical Medicine-Neurosurgery, University of Eastern Finland and Department of Neurosurgery, Kuopio University Hospital, Kuopio, Finland; [2]MRC Oulu, University of Oulu, Oulu, Finland; [3]Institute of Clinical

Medicine-Neurology, University of Eastern Finland and Department of Neurology, Kuopio University Hospital, Kuopio, Finland
**Correspondence:** V. Korhonen
*Fluids and Barriers of the CNS* 2018, **15(Suppl 1)**:A60

**Introduction:** The pathophysiology of iNPH is still unknown. However, there is evidence of comorbid vascular diseases. The familial occurrence of iNPH may be as high as 5–16% among Finnish elderly. The most common AD-related risk-loci do not seem to be overexpressed in iNPH. The copy number loss of *SFMBT1* gene is the most potential identified genetic risk factor in iNPH among Japanese elderly. There is also a slight evidence that allelic variation of *SFMBT1* gene may increase the risk of vascular diseases and diabetes potentially plausibly explaining vascular comorbidity in iNPH. We set out to investigate the copy number loss of *SFMBT1* among Finnish patients with iNPH and their relatives.
**Methods:** The study cohort consisted of 67 patients with shunt-operated iNPH and 96 relatives. The copy number loss was detected using quantitative-PCR.
**Results:** The copy number loss in the intron 2 of the SMBT1 was identified in 10.8% of shunted iNPH patients and in 9.5% of relatives. No statistical difference was detected between the groups ($p = 1$).
**Conclusions:** The copy number loss within intron 2 of *SFMBT1* was less prevalent compared to Japan. This indicates there may be populational differences in genetics of familial iNPH. Heterogenic hypotheses and further genetic studies are needed to clarify the elusive familial aggregation and pathophysiology of iNPH.

## A61

### Long-term outcome of shunt surgery in patients with aneurysmal subarachnoid hemorrhage

H. Adams, V. Leinonen, A. Lindgren, J. Frösen, M. I. von und zu Fraunberg, J. Huttunen, T. Koivisto, J. E. Jääskeläinen, T. J. Huttunen
Neurosurgery KUH NeuroCenter, Kuopio University Hospital, and Faculty of Health Sciences, School of Medicine, Institute of Clinical Medicine, University of Eastern Finland, Kuopio, Finland
**Correspondence:** T. J. Huttunen
*Fluids and Barriers of the CNS* 2018, **15(Suppl 1)**:A61

**Introduction:** Shunt-dependent hydrocephalus (SDHC) after aneurysmal subarachnoid hemorrhage (aSAH) is a common sequalae leading to poor neurological outcome, and predisposing to various interventions, admissions and complications. We evaluated the long-term outcome of shunted aSAH patients and the associated complications in a population-based setting.
**Methods:** The Kuopio sIA Database includes all aSAH cases from the defined catchment population in Eastern Finland. Patients who underwent shunt surgery due to post-hemorrhagic hydrocephalus following aSAH between 1990 and 2012 were included. Medical charts, operative reports, imaging studies, and clinical follow-up evaluations were analyzed and patients were followed up till 31, December 2015.
**Results:** From the total of 1850 aSAH patients, 275 aSAH patients with SDHC were included with a mean (SD) follow up time of 8 (7) years. The overall shunt complication rate was 33% over the follow-up period. Younger age ($p = 0.007$) and requirement of external ventricular drainage EVD ($p = 0.042$) during the index admission were associated with increased risk of shunt revision. The most used valve was fixed medium/high pressure (> 5 mmHg) one, fixed low pressure valves ($\leq$ 5 mmHg) was used in 10% of cases, and valveless shunts were used in 5% of patients. Only 7% of valves were adjustable, but showing lower revision rates as compared to fixed setting valves ($p = 0.042$). The most common shunt complications were valve failure (13%), infection (8%), ventricular catheter obstruction (7%), with mean time to revision of 90 months (SD 33). Timing of shunting was not correlated with revision rates ($p = 0.806$) or with infection rates ($p = 0.993$). Instead, patients that underwent a shunt revision had higher risk of multiple revisions if they received their first shunt very early following the aSAH ($p = 0.039$).
**Conclusion:** Long-term outcomes of shunt surgery and its complications in aSAH patient can be under-reported in a non-population based setting. Our study identified the incidence of shunt complications, also studied potential independent risk factors for complications. Age and initial requirement of EVD were associated with higher revision risk, whereas shunted aSAH patients with adjustable valve had lower risk of shunt revision in our cohort.

## A62

### ICP dynamics and brain water content in idiopathic intracranial hypertension

S. Qvarlander[1], V. Leinonen[2], E. Martoma[2], A. P. Elomaa[2], T. Puustinen[2], J. E. Jääskeläinen[2], J. Malm[3], T. Huttunen[2]
[1]Department of Radiation Sciences, Umeå University, Umeå, Sweden; [2]Department of Neurosurgery, Kuopio University Hospital, Kuopio, Finland; [3]Department of Pharmacology and Clinical Neuroscience, Umeå University, Umeå, Sweden
**Correspondence:** T. Huttunen
*Fluids and Barriers of the CNS* 2018, **15(Suppl 1)**:A62

**Introduction:** The pathophysiology of IIH is still unclear and no single theory fully explains the development of the raised intracranial pressure (ICP) causing the condition and resulting in headache, papilledema and vision loss. Disturbed cerebrospinal fluid (CSF) hydrodynamics affecting ICP have been suggested to affect the pathogenesis. We aim to characterize the ICP dynamics of IIH to investigate how these are affected by the disease and treatment.
**Methods:** To define a post-treatment group (attempted weight loss and medication for at least 3 months), 35 patients diagnosed with IIH and admitted from a defined catchment area between January 1, 2000 and December 31, 2016 were reviewed. Enrollment of pre-treatment subjects, i.e. patients that receive a new diagnosis of IIH, is on-going. All demographic, clinical, medical charts and imaging findings are recorded. ICP and indicators of CSF dynamics, including ICP pulse amplitude and RAP, are calculated based on pressure measurements with a CELDA® infusion apparatus. Volumetric analysis of white and gray matter and CSF has been performed for a subset of subject.
**Results:** 11 subjects that had been investigated with CELDA post-treatment were included. In the pre- treatment group, 4 CELDA investigations have been completed. Preliminary results will be presented.
**Conclusions:** We hope that the results will support the hypothesis that IIH patients have increased water content of grey matter and reduced intracranial compliance and that after treatment this improves.

## A63

### Laparoscopic-guided distal ventriculoperitoneal shunt insertion improves shunt outcome in adult patients: results of a cohort study with 222 patients experiencing 268 shunt operations

A. M. Isaacs[1], C. Ball[2], G. Urbaneja[1], R. Holubkov[3], M. G. Hamilton[1,2]
[1]Department of Clinical Neurosciences, University of Calgary, Calgary, Canada; [2]Department of Surgery, University of Calgary, Calgary, Canada; [3]Department of Medicine, University of Calgary, Calgary, Canada
**Correspondence:** A. M. Isaacs
*Fluids and Barriers of the CNS* 2018, **15(Suppl 1)**:A63

**Introduction:** Hydrocephalus is a chronic disease with multiple etiologies presenting at varying ages from birth to old age. The main treatment of hydrocephalus is surgical diversion of CSF from the cerebral ventricles, commonly to the peritoneal cavity using a shunt. One of the major challenges in managing hydrocephalus stems from the fact that 40–60% of shunts fail within the first 2 years. In adults, 80–90% of those failures are due to peritoneal catheter malfunction.
**Methods:** A prospective adult patient shunt outcome registry was established in January 2012 and concurrent patients enrolled. Distal shunt malfunction was confirmed by: (1) clinical deterioration in symptom control, and (2) abnormal structural or functional findings in CT/MRI scan, nuclear medicine shunt study or shunt X-ray. In April 2015, a laparoscopy-guided approach to place the distal catheter under direct visualization over the dome of the liver was initiated to attempt a reduction in distal catheter failure rates. We performed a retrospective analysis of all adult patients who underwent ventriculoperitoneal shunt insertion (VPSI) or revision (VPSR) at the FMC from January 2012 to May 2017.

**Results:** We have treated 222 adult patients for hydrocephalus of different etiologies with 177 patients having a new shunt insertions (VPSI) and 51 patients presenting with an established VP shunt and in need of a shunt revision (VPSR). A total of 177 VPSI and 91 VPSR occurred. There were 268 surgeries: 135 with laparoscopic-guided distal catheter placement (88 VPSI; 45 VPSR), and 114 with the standard surgical approach. Patients undergoing the standard surgical technique had 46.5% shunt failure at 752 days (55.4% failure at 1685 days), while patients undergoing the laparoscopic surgical technique had 27% shunt failure at 747 days. The laparoscopic surgical technique resulted in significantly lower VPSI failure rates at 2 year followup ($p = 0.046$ logrank test) and was protective for shunt revision (after the initial VPSI) with a hazard ratio of 0.53 compared with the standard surgical technique ($p = 0.049$). The laparoscopic technique was also superior to standard technique for lower failure rates after a "first VPSR".
**Conclusions:** The initiation of a laparoscopy-guided approach to place the distal catheter under direct visualization over the dome of the liver significantly reduced shunt obstruction after a new VP shunt insertion.

## A64

### Global epidemiology of hydrocephalus: systematic review of prevalence and birth surveillance report of incidence

A. M. Isaacs, D. Yavin, A. Hockley, J. Riva-Cambrin, M. Lowerison, J. Dronyk, G. Urbaneja, N. Jette, T. Pringsheim, M. G. Hamilton
University of Calgary, Calgary, Canada
**Correspondence:** A. M. Isaacs
*Fluids and Barriers of the CNS* 2018, **15(Suppl 1)**:A64

**Introduction:** Hydrocephalus is a chronic disease with multiple etiologies presenting at varying ages from birth to old age. However, the incidence of hydrocephalus is typically only described as ranging between 200 and 400 per 100,000 live births, usually with no reference to prevalence or other age ranges.
**Methods:** A systematic review of the medical literature was performed using a search strategy for population-based studies reporting prevalence of hydrocephalus in all ages. Two reviewers independently reviewed all the abstracts, full text articles and abstracted data using standardized forms. Reported incidence of hydrocephalus and spina bifida 2008–2014 from 36 countries were obtained from The International Clearinghouse for Birth defects' database. Meta-regression was completed by data source and diagnostic method.
**Results:** The search identified 2460 abstracts of which 148 were reviewed as full text articles and 57 met all eligibility criteria. Incidence data from 42 birth surveillance programs which included 166 million births were analyzed. Prevalence data were predominantly treatment-based (shunt insertion) and poorly reported for adults. The mean incidence of hydrocephalus in infants was 56 per 100,000 (95% CI 43–69). The registries exclude spina-bifida-associated hydrocephalus and postnatally acquired hydrocephalus from this category. The mean incidence of spina bifida was 45 per 100,000 (95% CI 37–52). Assuming a conservative estimate that 80% of patients with spina-bifida have associated hydrocephalus, the estimated total incidence of hydrocephalus in infants approaches 109 per 100,000 (95% CI 85–133). The mean prevalence of hydrocephalus in the pediatric population ($n = 25$ studies) was 100 per 100,000 (95% CI 64–158). The mean prevalence of hydrocephalus in all adults less than 65 years of age ($n = 7$ studies) was 86 per 100,000 (95% CI 65–114). The mean prevalence of hydrocephalus in adults over 65 years of age ($n = 6$ studies) was 255 per 100,000 (95% CI 19–492). Prevalence increased with each decade after 65 years of age.
**Conclusions:** The incidence of congenital hydrocephalus is reported at 109 per 100,000 from birth defects registries. The prevalence of hydrocephalus is well described in the pediatric population. The prevalence of hydrocephalus in the elderly (age over 65) is variable from one study to another with a wide range from 18 to 492 per 100,000. Inadequate information is available regarding the incidence of hydrocephalus in adults or the elderly. Further information is needed regarding the incidence and prevalence of hydrocephalus, especially in the adult and elderly patient population.

## A65

### A longitudinal observation of the non-desh inhabitants: from a cohort study of idiopathic normal pressure hydrocephalus (INPH) in the community-dwelling elderly in Takahata, Japan

C. Iseki, Y. Takahashi, L. Kimihira, R. Igari, H. Sato, S. Koyama, K. Suzuki, T. Kato
Division of Neurology and Clinical Neuroscience, Department of Internal Medicine III, Yamagata University School of Medicine, Yamagata, Japan
**Correspondence:** C. Iseki
*Fluids and Barriers of the CNS* 2018, **15(Suppl 1)**:A65

**Introduction:** The prospective study had started in 2000 and recruited 272 out of 350 (77.7%) inhabitants, the total number of 70-year-old in Takahata, a rural area of Japan. From the observation until 2010, the incidence of idiopathic normal pressure hydrocephalus (iNPH) was estimated as 1.2/100 persons per year. We used the DESH (disproportionately enlarged subarachnoid space hydrocephalus) for patients selection in addition to the Evans index > 0.3, though, there was few epidemiological community-based data for the non-DESH subjects. We aimed at knowing whether the inhabitants with non-DESH ventriculomegaly progress some disease.
**Methods:** The assessment was performed in 2016-17 with physical, neurological, laboratory, neurophsychological, and brain MRI exams.
**Results:** By the end of 2016 at the age of 86, 104 subjects had died and among the living 104 participants (60%), 7 (6.7%) were diagnosed as possible or provable iNPH for the Japanese guidelines of iNPH (Iseki et al. poster, WCN 2017). Among 19 subjects of the non-DESH ventriculomegaly in 2000, 17 subjects were followed in 2016, of whom 6 had died with cerebrovascular disease, cancer, or vascular dementia, and living 11 subjects included severe Alzheimer dementia (AD) (3), sever vascular dementia (1), depression with dementia (1), Mild Cognitive Impairment (2), possible iNPH (3), and asymptomatic ventriculomegaly with features of iNPH on MRI; AVIM(1). Of 3 AD patients, 2 were difficult to walk and one had experienced transient improvement of cognitive function by the tap test.
**Conclusions:** The non-DESH elderly developed symptoms of dementia and/or gait disturbance, which were difficult to judge if iNPH coexists.

## A66

### Predictability of cognition tests for shunt effectiveness in INPH

M. Ishikawa[1,2], S. Yamada[2,3], K. Yamamoto[3]
[1]Rakuwa Vila Ilios, Kyoto, Japan; [2]Normal Pressure Hydrocephalus Center, Otowa Hospital, Kyoto, Japan; [3]Department of Neurosurgery, Otowa Hospital, Kyoto, Japan
**Correspondence:** M. Ishikawa
*Fluids and Barriers of the CNS* 2018, **15(Suppl 1)**:A66

**Introduction:** The idiopathic normal pressure hydrocephalus (iNPH) is a disease of the elderly, and cerebrospinal fluid (CSF) shunt surgery is known to be effective. CSF tap test is useful for prediction of shunt effectiveness. We have shown that area under the curve (AUC) computed from receiver-operating characteristic curve was a high value of 0.81 in timed up-and-go test (TUG) on day 1. However, predictability of cognition tests is not well investigated. We studied the predictability of cognition tests in Otowa hospital.
**Methods:** The shunt surgery was done in 61 patients with probable iNPH and postoperative assessment was done at 3 months after surgery. Lumbar CSF in 30 ml was removed at tap test and tests for gait and cognition were done before the tap, on day 1 and day 4. Tests for gait included TUG and 10-m walk test. Cognition tests included mini-mental state examination, frontal assessment battery (FAB) and trail-making test. Positive tap test was defined as improvement of 10% or more. AUC values were computed using software of the R Foundation.
**Results:** Positivity of cognition tests ranged from about 25% to 45% in three measures on day 1 and there was no statistical difference between day 1 and day 4. AUC values for cognition were around 0.50. Among them, FAB on day 4 was highest, but it was only 0.62.
**Conclusions:** The predictability of cognition tests was not high enough, compared to that of gait tests. We need more sensitive test for cognition.

## A67

### Hyperlipidemia is associated with lack of improvement after shunt surgery in idiopathic normal pressure hydrocephalus

H. Israelsson[1], A. Eklund[2], J. Malm[1]
[1]Department of Pharmacology and Clinical Neuroscience, Umeå University, Umeå, Sweden; [2]Department of Radiation Sciences, Umeå University, Umeå, Sweden
**Correspondence:** H. Israelsson
*Fluids and Barriers of the CNS* 2018, **15(Suppl 1)**:A67

**Introduction:** The amount of comorbidity and vascular disease are considered important predictors of prognosis and outcome of shunt surgery in idiopathic normal pressure hydrocephalus (INPH). However, few studies have investigated the impact of cardiovascular risk factors (CVRF). The objective was to investigate how multiple CVRF influence outcome of shunting in INPH.
**Methods:** All non-demented INPH patients (age 60–85 years) who underwent shunting in Sweden 2008–2010 were included (n = 176). Prospectively assessed CVRF were: hypertension, hyperlipidemia, diabetes, obesity, depression, smoking, diet, alcohol, and physical activities. Cardiovascular, cerebrovascular and peripheral vascular diseases were assessed. Outcome of surgery was measured 3 and 12 months after shunting by gait, modified Rankin Scale and minimental state examination.
**Results:** The total amount of CVRF correlated with severity of gait disturbance ($p = 0.046$) but not with outcome of surgery. Limiting the analysis to the CVRF previously proven to be the most important in INPH (depression, hyperlipidemia, diabetes, obesity), a higher number of those correlated with lack of improvement (OR 1.67, 95% CI 1.09–2.55, $p = 0.019$). When analyzing the CVRF separately, only hyperlipidemia was more common among unimproved patients (48% vs. 27%) and remained an independent predictor of outcome of surgery (OR 2.54, 95% CI 1.19–5.41, $p = 0.016$). Vascular disease did not influence outcome of surgery.
**Conclusions:** In the preoperative evaluation of INPH, screening for CVRF in general and hyperlipidemia in particular should be performed. The importance of hyperlipidemia and possible effect of antilipid treatment in INPH needs to be further researched. INPH patients should not be excluded from surgery due to vascular comorbidity.

## A68

### Patients' information in hydrocephalus: why not comics?

T. Jacquesson[1,2], M. Berhouma[1], V. Delabar, R. Mannet[1], E. Jouanneau[1]
[1]Department of Neurosurgery B, Neurological Hospital Pierre Wertheimer, Hospices Civils de Lyon, Lyon, France; [2]Department of Anatomy, University of Lyon, Lyon, France
**Correspondence:** T. Jacquesson
*Fluids and Barriers of the CNS* 2018, **15(Suppl 1)**:A68

**Introduction:** Information to patients has become a daily challenge in the recent medical practice. Nowadays, patients and their family want to understand strategies for diagnosis and treatment as well as disease origins. That is especially the case for hydrocephalus which leads to many questions including anatomical and technical details. Therefore, all medical practitioners need communication tools providing simple, clear and appropriate information. As an answer to this new challenge, we present an unusual information tool for hydrocephalus: comics.
**Method:** In our neurosurgical center's experience in hydrocephalus management, we collected all patients' questions about the disease: anatomical basics, diagnosis proof, treatment strategy and follow-up. A scenario was written to answer to all questions. Dedicated comics were then edited thanks to the collaboration of a professional drawer. Both text and pictures were approved by our neurosurgical team. This new information tool was subsequently addressed to hydrocephalus patients in parallel with classical oral information.
**Results:** The document is a A5-format book of 14 pages. It details all the steps through which a hydrocephalus patient goes from first symptoms to follow-up. It insists on the anatomical rationale and the technical subtleties of surgical procedures as the third ventriculostomy and the ventriculo-peritoneal shunt. Also, it explains how surgery efficiency is controlled and drainage flow is adapted.
**Conclusions:** Comics offer new, accurate, comprehensive and reassuring information for hydrocephalus patients. Obviously, such tool does not replace information provided in any medical practitioner's consultation. The first patients' feedbacks are promising, but further studies are required to improve and validate it.

## A69

### Determining NFL in plasma for patients with idiopathic normal pressure hydrocephalus

A. Jeppsson[1], Å. Sandelius[2], K. Blennow[2], H. Zetterberg[2], C. Wikkelsø[1], P. Hellström[1], M. Tullberg[1]
[1]Hydrocephalus Research Unit, Department of Clinical neuroscience, Institute of Neuroscience and Physiology, The Sahlgrenska Academy, University of Gothenburg, Gothenburg, Sweden; [2]Clinical Neurochemistry Laboratory, Department of Psychiatry and Neurochemistry, Institute of Neuroscience and Physiology, the Sahlgrenska Academy, University of Gothenburg, Gothenburg, Sweden
**Correspondence:** A. Jeppsson
*Fluids and Barriers of the CNS* 2018, **15(Suppl 1)**:A69

**Introduction:** CSF sampling can be invasive and technically challenging. Neurofilament light (NFL), mirroring neuronal damage, is in wide clinical use and has shown to be elevated in patients with iNPH. The Simoa platform, able to detect low concentrations, has opened the possibility of detecting brain-derived proteins in blood. The aim of this study was to examine (1) if $NFL_{plasma}$ is elevated in patients with iNPH; and (2) the relationship between NFL CSF and plasma.
**Methods:** $NFL_{CSF}$ and $NFL_{plasma}$ were determined pre- and postoperatively in 50 consecutively included iNPH patients. Patients were evaluated preoperatively and at 6 months follow up by the iNPH scale. $NFL_{plasma}$ was also determined in 49 healthy, age- and gender matched controls. $NFL_{CSF}$ was determined using standard ELISA-based methods and $NFL_{plasma}$ by the Simoa platform.
**Results:** Thirty-six (72%) patients were improved at follow-up. $NFL_{plasma}$ was elevated in iNPH patients (44.7; 30.4–64.4, median; Q1–Q3) compared to controls (33.0; 26.3–49.6, $p < 0.05$). $NFL_{CSF}$ increased from 1415; 985–2063 at baseline to 1955; 1500–2083 postoperatively ($p < 0.001$) whereas $NFL_{plasma}$ remained unchanged (43.3; 28–55.1) $NFL_{plasma}$ correlated with $NFL_{CSF}$ pre-, and postoperatively ($r_s = 0.629$ $p < 0.001$, $r_s = 0.722$, $p < 0.0001$). No correlation was seen with clinical parameters.
**Conclusions:** In this study we show an elevation of NFL in plasma in iNPH patients reflecting neuronal dysfunction. Together with a correlation between levels of NFL in CSF and plasma, our results indicate a promising role for CNS-derived biomarkers in blood for patients with iNPH.

## A70

### Predicting dynamics of CSF biomarkers by tap test in INPH

N. Jingami[1,2], K. Uemura[1,3], M. Asada[1,2], A. Kuzuya[1,2], S. Yamada[4], M. Ishikawa[4], T. Kawahara[5], T. Iwasaki[5], M. Atsuchi[5], R. Takahashi[1], A. Kinoshita[2]
[1]Department of Neurology, Kyoto University Graduate School of Medicine, Kyoto, Japan; [2]Department of Human Health Sciences, Kyoto University Graduate School of Medicine, Kyoto, Japan; [3]Department of Neurology, Ishiki Hospital, Kagoshima, Japan; [4]Normal Pressure Hydrocephalus Center, Rakuwakai Otowa Hospital, Kyoto, Japan; [5]Normal Pressure Hydrocephalus Center, Atsuchi Neurosurgical Hospital, Kagoshima, Japan
**Correspondence:** N. Jingami
*Fluids and Barriers of the CNS* 2018, **15(Suppl 1)**:A70

**Introduction:** Idiopathic normal pressure hydrocephalus (iNPH) is one of common age-related neurological diseases. It is reported that, approximately half of iNPH patients have coexisting pathology of Alzheimer's disease. However, we have previously reported that lower cerebrospinal fluid (CSF) tau levels were observed in iNPH. In previous reports, tau levels in ventricular CSF (VCSF) were significantly elevated compared to ones in lumbar CSF (LCSF) in iNPH. We hypothesized that some biomarkers in LCSF, including tau, could not accurately reflect the brain pathological conditions in iNPH.
**Methods:** To conveniently predict dynamics of CSF biomarkers in iNPH, we analyzed CSF levels of tau, Amyloid beta (Aβ) and leucine rich

α-2 glycoprotein (LRG) in iNPH patients who underwent tap test (30 ml CSF drainage). At tap test, we separately collected CSF at the first drip (FD: 0–1 ml) and at the last drip (LD: 29–30 ml). 1 ml of VCSF was additionally collected from patients who underwent on ventriculoperitoneal shunt surgery. The levels of tau, Aβ and LRG were measured by using ELISA specific for each biomarker. Gait and cognitive function were evaluated by Timed UP & Go Test and MMSE, respectively.

**Results:** The CSF levels of Aβ42 as well as LRG were significantly reduced in LD compared to ones in FD. Conversely, the CSF tau levels were significantly elevated in LD compared to ones in FD. Response to tap test in gait and cognitive function was closely associated with the levels of tau and LRG in LD, respectively.

**Conclusions:** We suggested that the dynamic changes are different among measured biomarkers. The above data suggest that CSF in LD sampled in tap test likely reflects an aspect of VCSF, contributing to predicting shunt effectiveness in iNPH patients.

## A71

### Long-term outcomes of shunting for idiopathic intracranial hypertension

A. J. Joannides[1], C. McMahon[2], H. K. Richards[1], J. D. Pickard[1]
[1]Department of Clinical Neurosciences, University of Cambridge, Cambridge, UK; [2]The Walton Centre NHS Foundation Trust, Liverpool, UK
**Correspondence:** A. J. Joannides
*Fluids and Barriers of the CNS* 2018, **15(Suppl 1)**:A71

**Introduction:** CSF shunting has an important role in the management of refractory idiopathic intracranial hypertension (IIH). Shunt-related complications can be a source of significant morbidity arising from multiple revision procedures. On this background, we investigated the incidence and outcomes of shunting for patients with IIH within the UK population.

**Methods:** Patients undergoing a CSF shunting procedure with a diagnosis of IIH recorded on the UK Shunt Registry between January 1995 and 31st December 2014 were included in the analysis. Patients with more than one underlying CSF disorder diagnosis were excluded. Analysis of time to revision was performed using the Kaplan–Meier method and statistical significance determined with the log-rank test.

**Results:** A total of 2014 patients meeting the inclusion criteria were identified, undergoing 4042 procedures across 45 centres. The majority of patients were female (1692; 84%), and median age at surgery was 31 years [IQR 23–39 years]. The commonest reasons for shunt revision included underdrainage (54%), catheter migration (12%) and disconnection (9.2%). New shunt insertions accounted for 1776 of recorded procedures, including 788 ventricular shunts and 832 lumbar shunts. Median survival of new shunts was higher for ventricular as compared to lumbar shunts (736 weeks vs. 679 weeks; $p = 0.005$).

**Conclusions:** Our data suggests that new shunts inserted for IIH are associated with a long time to first revision, with the commonest reasons for this relating to mechanical failure. Furthermore, ventricular shunts have a marginally longer period to first revision compared to lumbar shunts. Evaluation of other associated operative factors will be required to determine potential underlying reasons for the observed differences.

## A72

### The occasional discrepancy between patient reported and clinical outcomes in idiopathic normal pressure hydrocephalus

A. Junk kari[1], R. P. Roine[2,3], A. Luikku[4], T. Rauramaa[5], H. Sintonen[6], O. Nerg[4], A. M. Koivisto[4], A. Häyrinen[1], H. Viinamäki[7], H. Soininen[8], J. E. Jääskeläinen[1], V. Leinonen[1]
[1]Neurosurgery of NeuroCenter, Kuopio University Hospital and University of Eastern Finland, Kuopio, Finland; [2]Research Centre for Comparative Effectiveness and Patient Safety, University of Eastern Finland, Kuopio, Finland; [3]Group Administration of Helsinki and Uusimaa Hospital District, Helsinki, Finland; [4]Neurology of NeuroCenter, Kuopio University Hospital and University of Eastern Finland, Kuopio, Finland; [5]Department of Pathology, Kuopio University Hospital and University of Eastern Finland, Kuopio, Finland; [6]Department of Public Health, University of Helsinki, Finland; [7]Department of Psychiatry, Kuopio University Hospital and University of Eastern Finland, Kuopio, Finland; [8]Department of Neurology, University of Eastern Finland, Kuopio, Finland
**Correspondence:** A. Junk kari
*Fluids and Barriers of the CNS* 2018, **15(Suppl 1)**:A72

**Introduction:** Occasionally a favorable clinical disease-specific outcome does not reflect into improved generic health-related quality of life (HRQoL) in patients with idiopathic normal pressure hydrocephalus (iNPH) 1 year after the installation of the cerebrospinal fluid (CSF) shunt. Our aim was to identify factors causing this discrepancy.

**Methods:** The 1-year HRQoL outcome of 141 iNPH patients was evaluated using the generic 15D instrument, in which the minimum clinically important change/difference on the 0–1 scale has been estimated to be $\pm$ 0.015. A 12-point iNPH grading scale (iNPHGS) was used as a clinical disease-specific outcome measure, in which one point decrease is considered to be clinically important. We identified 29 (21%) iNPH patients from our prospective study, whose HRQoL deteriorated or remained the same despite of a favorable iNPHGS outcome. We analyzed this discrepancy using patients' clinical variables and characteristics.

**Results:** Multivariate binary logistic regression analysis indicated that a higher (worse) iNPHGS score at baseline (adjusted OR, 1.7; 95% CI, 1.3–2.3; $p < 0.001$), comorbid chronic pulmonary disease (40% vs. 20%; adjusted OR, 17.89; 95% CI, 3.6–89.9; $p < 0.001$) and any comorbid non-metastatic tumor (62% vs. 17%; adjusted OR, 11.5; 95% CI, 1.5–85.3; $p = 0.017$) predicted discrepancy between iNPHGS and 15D outcomes.

**Conclusions:** Frail patients suffering from certain pre-existing comorbidities may not experience improvement in generic HRQoL despite of a favorable clinical disease-specific response. Acknowledging the comorbidity burden of the patient may help clinicians and the patients to understand the conflict between patient reported and clinical outcomes.

## A73

### Otological symptoms are common in patients with idiopathic intracranial hypertension and associate with high prevalence of pulmonological symptoms

H. K. Jyrkkänen, M. Iso-Mustajärvi, T. Willberg, A. Dietz, J. Randel, V. Leinonen, A. P. Elomaa, T. Puustinen, J. E. Jääskeläinen, T. Huttunen
Kuopio University Hospital, Kuopio, Finland
**Correspondence:** H. K. Jyrkkänen
*Fluids and Barriers of the CNS* 2018, **15(Suppl 1)**:A73

**Introduction:** Idiopathic intracranial hypertension (IIH) is a rare condition affecting obese woman of childbearing age. Although IIHs pathophysiology is unknown, its profile is invariably characterised by raised intracranial pressure (ICP), papilledema and visual loss. Pulmonological disorders such as sleep apnea, and otological symptoms such as tinnitus and hearing loss have been previously reported in IIH patients. Obesity stresses the respiratory system, but otological symptoms could be directly linked to increased ICP and consequent increases in perilymphatic and venous sinus pressures. Thus, we characterized the ICPs and the presence of otological and respiratory symptoms of IIH patients in a population-based setting.

**Methods:** A total of 35 patients with IIH diagnosis admitted from a defined catchment area between 2000 and 2016 were reviewed. All otological and respiratory symptoms were reviewed from patient records.

**Results:** Total of 80% were females. At the time of diagnosis the mean for age was 30.1 years, for BMI 36.2 $kg/m^2$, for CSF opening pressure (OP) 31.2 mmHg, and for follow-up time 4.4 years, respectively. Tinnitus occurred in 43 and 6% participants had documented hearing loss. Presentation of otological symptoms were associated with increased presence of papilledema ($p = 0.045$), higher BMI (37.9 vs. 34.4 $kg/m^2$) and to a poorer outcome after treatment (36% vs. 23%), but not to sex, age or OP. The patients with otological symptoms were more often shunted (44% vs. 28%) and had higher presentation of pulmonological symptoms(56% vs. 5%) ($p = 0.002$) as compared with patients without otological symptoms.

**Conclusions:** Our results suggest that otological symptoms are common in IIH patients and that they should be screened due to increased morbidity and possible pulmonological disorders.

## A74

### Beginning of preclinical stage of idiopathic normal pressure hydrocephalus on MRI

Y. Kajimoto, A. Tucker, T. Kuroiwa
Department of Neurosurgery, Osaka Medical College, Takatsuki, Japan
**Correspondence:** Y. Kajimoto
*Fluids and Barriers of the CNS* 2018, **15(Suppl 1)**:A74

**Introduction:** The preclinical stage of idiopathic normal pressure hydrocephalus (iNPH) is known as Asymptomatic Ventriculomegaly with Features of iNPH on MRI (AVIM). However, the age at which AVIM begins to appear is not clear. In this study, MRI of healthy people who underwent brain docks was quantitatively evaluated prospectively, and the prevalence of AVIM per age was investigated.
**Methods:** We prospectively surveyed 632 people who underwent brain MRI at brain checkup of the Osaka Medical College from October 2011 until March 2013. We measured ventricular size with Evans index in all cases. We selected a case with ventricular enlargement (0.3 or more in the Evans index), and quantified the ratio of the subarachnoid space of medial parietal lobe from the T2 axial image using image analysis software. We defined AVIM as a case where the Evans index was 0.3 or more and the proportion of the subarachnoid space was 13% or less.
**Results:** The proportion of AVIM was 0% in the 50 s, but it was 1.3% in the 60 s and 2.8% in the age over 70 years.
**Conclusions:** AVIM began to appear in 60 s, and its proportion increased with age. As a result, it admitted to 2.8% of healthy elderly people over 70 years old.

## A75

### Intermittent gait disturbance in idiopathic normal pressure hydrocephalus

Y. Kajimoto[1], Y. Nikaido[2], A. Tucker[1], K. Kuroda[2], H. Ohno[2], T. Akisue[3], R. Saura[2], T. Kuroiwa[1]
[1]Department of Neurosurgery, Osaka Medical College, Takatsuki, Japan; [2]Department of Rehabilitation Medicine, Osaka Medical Collage, Takatsuki, Japan; [3]Department of Rehabilitation Science, Graduate School of Health Sciences, Kobe University, Kobe, Japan
**Correspondence:** Y. Kajimoto
*Fluids and Barriers of the CNS* 2018, **15(Suppl 1)**:A75

**Introduction:** We identified a pattern of intermittent gait disturbance (IGD) observed in the early stages of idiopathic normal pressure hydrocephalus (iNPH). The purpose of this study is to clarify the temporal gait profile of IGD and explore the clinical implications of IGD.
**Methods:** Fourteen consecutive iNPH patients with subtle short distance gait instability were treated by cerebrospinal fluid (CSF) shunting. Among these patients, seven presenting with progressive gait worsening after several minutes of gait loading were prospectively enrolled in the study. Gait and cognitive functions were evaluated by the 6-min Walk Test (6MWT) and the Mini-Mental State Examination (MMSE), respectively. Long-term follow-up (mean 49 months) of gait and cognitive function was performed in five patients.
**Results:** All patients demonstrated features of IGD during the 6MWT, characterized by a progressive pattern of decreased gait speed and step length with increased cadence and an absence of leg pain. Postoperatively, IGD improved in all patients. Improved gait and cognitive function (MMSE range of 27–30) were well preserved during the follow-up period.
**Conclusions:** IGD was observed in early stage of iNPH and may serve as an important clinical diagnostic marker for identifying iNPH patients with mild gait symptoms.

## A76

### Lumbar catheter insertion by the fluoroscopic-guided paramedian approach for cerebrospinal fluid shunting: assessment of safety and accuracy

Y. Kajimoto, A. Tucker, T. Kuroiwa
Department of Neurosurgery, Osaka Medical College, Takatsuki City, Japan
**Correspondence:** Y. Kajimoto
*Fluids and Barriers of the CNS* 2018, **15(Suppl 1)**:A76

**Introduction:** Spinal catheter insertion of lumboperitoneal (LP) shunt surgery for idiopathic normal pressure hydrocephalus (iNPH) is frequently associated with technical difficulties related to obesity and vertebral deformities of aging. The aim of this study is to elucidate the accuracy and safety of image-guided spinal catheter insertion by the paramedian approach (PMA)
**Methods:** We retrospectively analyzed 39 consecutive patients with iNPH treated by LP shunting with spinal catheter insertion via the PMA. We evaluated the success rate of catheter insertion and the number of changes in puncture location. To measure accuracy of catheter insertion, both vertical and horizontal deviation of the catheter dural penetration point from the center of the inter-laminar space was evaluated.
**Results:** The success rate for catheter insertion was 100% (39/39). The difficulty rate for catheter insertion measured by the number of changes in puncture location was 2.6% (1/39). No bloody punctures and surgical infections were observed. The accuracy of catheter insertion measured as the degree of deviation horizontally was $0.5 \pm 1.9$ mm and vertically $0.0 \pm 2.4$ mm. The rates of minor complications, including caudal catheter insertion, transient low pressure headache, and root pain, were 5.1% (2/39), 10.4% (4/39) and 0% (0/43), respectively. Subdural hematoma requiring surgery occurred in one case (2.6%). During the mean follow-up period of 36 months, spinal catheter rupture at the level of the spinous processes was not observed.
**Conclusions:** The fluoroscopic guided PMA for spinal catheter insertion is potentially a safe, accurate, and reliable method even for use in geriatric and obese patients.

## A77

### Increased water quantity in grey matter in idiopathic intracranial hypertension patients

E. Kallioniemi[1], R. Vanninen[2], J. Harju[2] V. Leinonen[3], A. P. Elomaa[3], T. Puustinen[3], J. E. Jääskeläinen[3], T. Huttunen[3]
[1]Departments of aClinical Neurophysiology bClinical Radiology, Kuopio University Hospital cDepartment of Applied Physics, University of Eastern Finland, Kuopio, Finland; [2]Department of Radiology, Kuopio University Hospital and University of Eastern Finland, Kuopio, Finland; [3]Neurosurgery of NeuroCenter, Kuopio University Hospital and University of Eastern Finland, Kuopio, Finland
**Correspondence:** E. Kallioniemi
*Fluids and Barriers of the CNS* 2018, **15(Suppl 1)**:A77

**Background:** Idiopathic intracranial hypertension (IIH) is a rare disease with unclear pathophysiology. Dysregulated cerebrospinal fluid (CSF) hydrodynamics has been suggested to increase cerebral brain water content or cerebral blood volume resulting in transependymal flow causing interstitial brain edema. Magnetic resonance (MR) imaging-based volumetric measurements of grey, white and CSF at diagnosis and post-treatment follow-up.
**Methods:** A total of 35 patients with IIH diagnosis admitted to the Kuopio University Hospital catchment area between 2000 and 2016 were reviewed. All demographic, clinical, medical charts and imaging findings were recorded. Brain volumes were calculated from T1 weighted MRI images by SPM 12 software. After applying all imaging requirements only three subjects were accepted for the final analysis.
**Results:** The mean age at the time of diagnosis was 25.3 (SD 9.1) years and the mean follow-up time was 2.3 (SD 0.58) years and all were females. The mean BMI at diagnosis was 34.5 $kg/m^2$, The mean CSF opening pressure at presentation was 32.4 mmHg (SD 4.2), and it reduced after treatment to 25.2 mmHg (SD 5.9). All patients were treated with acetatzolamide and weight loss, and one patient was treated with lumboperitoneal shunt. In all patients, the volume of grey matter reduced after treatment, whereas the volume of white matter increased. The volume of CSF space reduced after treatment in two subjects, but in one subject whose outcome was poor after treatment it increased.

**Conclusions:** The results suggest that IIH patients have increased grey matter volume which seems to reduce after treatment indicating potentially increased water content.

## A78

### Cost-effectiveness analysis of shunt surgery for idiopathic normal pressure hydrocephalus

M. Kameda[1], S. Yamada[2], M. Atsuchi[3], T. Kimura[4], H. Kazui[5], M. Miyajima[6], E. Mori[7], M. Ishikawa[2], I. Date[1]
[1]Department of Neurological Surgery, Okayama University Graduate School of Medicine, Dentistry and Pharmaceutical Sciences, Okayama, Japan; [2]Normal Pressure Hydrocephalus Center, Department of Neurosurgery and Stroke Center, Rakuwakai Otowa Hospital, Kyoto, Japan; [3]Department of Neurosurgery, Atsuchi Neurosurgical Hospital, Kagoshima, Japan; [4]Department of Neurosurgery, Dohtoh Neurosurgical Hospital, Hokkaido, Japan; [5]Department of Psychiatry, Osaka University Graduate School of Medicine, Osaka, Japan; [6]Department of Neurosurgery, Juntendo University Graduate School of Medicine, Tokyo, Japan; [7]Department of Behavioral Neurology and Cognitive Neuroscience, Tohoku University Graduate School of Medicine, Miyagi, Japan
**Correspondence:** M. Kameda
*Fluids and Barriers of the CNS* 2018, **15(Suppl 1)**:A78

**Introduction:** We showed therapeutic efficacy of shunt surgery for iNPH in SINPHONI and SINPHONI-2 study. Although therapeutic efficacy is important, cost-effectiveness analysis is equally valuable.

**Methods:** Using both a set of assumptions and using the data from SINPHONI and SINPHONI-2, we estimated the total cost of treatment for iNPH, which consists of medical expenses (e.g. operation fees) and costs to the long-term care insurance system in Japan. Regarding the natural course of iNPH patients, 10 or 20% of patients on each modified Rankin Scale (mRS) show aggravation every 3 months if the patients do not undergo shunt surgery. We performed cost-effectiveness analyses for the various scenarios, calculating the quality-adjusted life year (QALY) and the incremental cost-effective ratio (ICER).

**Results:** In the first year after shunt surgery, the ICER of VP shunt varies from 29,934 to 40,742 USD (aggravation rate 10 and 20%, respectively) and the ICER of LP shunt varies from 58,346 to 80,392 USD (aggravation rate 10 and 20%, respectively), which indicates that the shunt surgery for iNPH is a cost-effective treatment. Moreover, the total cost for iNPH patients will show a positive return on investment in as soon as 18 months (VP) and 21 months (LP).

**Conclusions:** Because the total cost for iNPH patients will show a positive return on investment within 2 years, shunt surgery for iNPH is a cost-effective treatment and therefore recommended. Because complications naturally affect the medical expenses, more appropriate surgical technique to reduce shunt revision should be invented.

## A79

### Longitudinal cognitive change in INHP after shunt opeartion

M. Kanai[1], N. Sasaguchi[2]
[1]Division of Neurology, NHO Takasaki-hospital, Takasaki, Japan; [2]Division of Neurosurgery, NHO Takasaki-hospital, Takasaki, Japan
**Correspondence:** M. Kanai
*Fluids and Barriers of the CNS* 2018, **15(Suppl 1)**:A79

**Introduction:** For cognitive impairment seen in idiopathic normal pressure hydrocephalus (iNPH), I paid my attention about improvements by treatment.

**Methods:** From June, 2010 to January, 2013, 65 patients had shunt operation. We evaluated the examination of cognitive function in mini-mental state examination (MMSE), frontal assessment battery (FAB), Tower of Hanoi problem (TOH) and Kohs block design test (Kohs).

**Results:** As for 65 subjexs, the average age is 78 years old with 30 men and 35 women. Lumbar vertebrae abdominal cavity shunting (LP shunt) 61 cases, ventriculoperitoneal shunting (VP shunt) was four cases. Just 15 cases were continued and evaluated them. In comparison with aggravation of MMSE, the drop of FAB and the Kohs score was not outstanding, and there was it by improvement to maintenance in eight of 15 cases. As for the TOH to evaluate executive function, an improvement effect was poor.

**Conclusions:** The memory decline was seen with aging, but was able to maintain the frontal lobe function. I think that it is necessary to cure at the stage when a symptom is earlier, and to intervene to maintain long-term improvement.

## A80

### Preoperative evaluation of thalamic volume might predict shunt responsiveness in patients with idiopathic normal pressure hydrocephalus

S. Kanno[1,2], E. Mori[2,3,4]
[1]Department of Neurology, Southmiyagi Medical Center, Shibata, Japan; [2]Department of Behavioral Neurology and Cognitive Neuroscience, Tohoku University, Sendai, Japan; [3]Cognitive Neurology and Memory Disorder Center, Nissei Hospital, Osaka, Japan; [4]Osaka University, Suita, Japan
**Correspondence:** S. Kanno
*Fluids and Barriers of the CNS* 2018, **15(Suppl 1)**:A80

**Introduction:** The aim of this study was to detect the difference in regional gray matter volume between shunt responders (SR) and shunt non-responders (SNR) in idiopathic normal pressure hydrocephalus (INPH) using voxel-based morphometry (VBM) and anatomical region of interest (ROI) analysis.

**Methods:** The twenty-eight consecutive INPH patients were enrolled. MRI of the brain and clinical measures were performed prior to both cerebrospinal fluid removal and shunt placement. Clinical measures were re-assessed approximately 1 year after shunt surgery to assess shunt responsiveness. Twenty of the initial INPH patients were SR and the other 8 patients were SNR. Whole and regional brain gray matter volumes were detected by three-dimensional spoiled gradient echo image. Preoperative gray matter volume maps were compared between the SR and SNR groups by using VBM. In addition, anatomical ROI analyses of the regions in which gray matter volumes were significantly different among the two groups in VBM were performed to validate the results of the voxel-based analysis.

**Results:** On a voxel-based statistical map and in anatomical ROI analysis, the volume of each thalamus in the SNR group was significantly smaller than that in the SR group. In addition, we found that the volume of bilateral thalami highly distinguished SR from SNR.

**Conclusions:** The poor volume of the bilateral thalami might reflect the co-morbidity with other CNS diseases in SNR because this finding is common in dementing neurodegenerative diseases. The preoperative volume of bilateral thalami could predict shunt responsiveness in INPH.

## A81

### Use of local anesthesia for lumboperitoneal shunt procedure in patents of idiopathic normal pressure hydrocephalus

T. Kawahara[1], M. Atsuchi[1], K. Takasaki[1], T. Iwasaki[1], K. Arita[2]
[1]Division of Neurosurgery, Atsuchi Neurosurgical Hospital, Kagoshima, Japan; [2]Department of Neurosurgery, Graduate School of Medical and Dental Sciences, Kagoshima University, Japan
**Correspondence:** T. Kawahara
*Fluids and Barriers of the CNS* 2018, **15(Suppl 1)**:A81

**Introduction:** Idiopathic normal pressure hydrocephalus (iNPH) is a common disease in elderly patients treated with cerebrospinal fluid shunting. Lumbo-peritoneal shunt (LPS) is effective and safe treatment like ventriculo-peritoneal shunt (VPS). General anesthesia is commonly used for LPS in many hospitals. While, in our hospital, LPS is performed under local anesthesia (LA) with modified neuroleptic anesthesia (NLA). We report our single-center experience of this method, and its usefulness and outcome for aged iNPH patients.

**Methods:** The patient is placed in a lateral position under modified NLA. And LA is administered before skin incision. We reviewed patients with LPS insertion under LA with modified NLA for last 6 years, and described their complications and outcomes.

**Results:** Between April 2011 and March 2017, all of 372 LPS were performed with this procedure without major complication. The patients' age ranged from 55 to 95 years old with mean of 78.36 ± 6.88 (SD). In nine patients, LPS placement was failed, because of difficulty in the spinal catheter insertion. Migration of abdominal catheter was the most common complication of LPS (3.5%). But it seems to have nothing to do with the way of anesthesia.

**Conclusions:** Our way of anesthesia provided sufficient sedation and analgesia during LP shunt procedure. And no intra- and postoperative complications due to this anesthesia resulted. As the placement of LPS is minimally invasive surgery for patients of hydrocephalus, administrations of powerful sedation or muscle relaxant agents are not necessarily required. This method may not be suitable for prolonged surgery, which can be anticipated preoperatively.

## A82

### Prognosis and comorbidity of idiopathic normal pressure hydrocephalus: outcome analysis in Japanese and Austrian cohort studies

T. Kawasaki[1], I. Akiguchi[1], Y. Watanabe[1], Y. Shirakashi[2], A. Miyake[2], H. Yagi[1], K. Nakajima[1], T. Watanabe[3], T. Nishikawa[3], H. Budka[4], W. Krampla[5], P. Fischer[6]
[1]Center of Neurological and cerebrovascular diseases, Koseikai Takeda Hospital, Kyoto, Japan; [2]Center of Higher Nervous Function, Uji Takeda Hospital, Uji, Japan; [3]Department of Health Science, Kyoto Koka Women's University, Kyoto, Japan; [4]Medical University of Vienna, Institute of Neurology, Vienna, Austria; [5]Department of Radiology, Danube Hospital, Vienna, Austria; [6]Department of Psychiatry, Danube Hospital, Vienna, Austria
**Correspondence:** T. Kawasaki
*Fluids and Barriers of the CNS* 2018, **15(Suppl 1)**:A82

**Introduction:** Brain autopsy and biopsy in patients with idiopathic normal pressure hydrocephalus (iNPH) often show Alzheimer's disease (AD) as well as vascular dementia/Binswanger's disease (VD/BD) pathology.

**Methods:** We conducted, therefore, the following two studies on prognosis and comorbidity in iNPH.

**Results:** 1. Vienna Trans-Danube Aging Study; MRI features of iNPH/disproportionately enlarged subarachnoid space hydrocephalus (iNPH-DESH) among 503 participants of community-based cohort at age 75. (1) 8 out of 503 participants with iNPH-DESH (1.6%) were detected at baseline. At follow-up after 90 months, mortality rates in iNPH-DESH and normal control were 25.0 and 10.9%, respectively. (2) MRI features of BD (BD-MRI) and meningiomas were comorbid in 1case at baseline (12. 5%) and AD in 2 cases at 30 month follow-up (25%). 2. Takeda Hospital iNPH Studies; (a) Consecutive in-patient cohort in 122 probable iNPH and (b) postoperative follow-ups with 54 definite iNPH (a) At the baseline; 64 patients (52.5%) comorbid with dementia including AD (40.2% of those; MMSE score lower than 22), 49.2% comorbid with cerebrovascular diseases including BD-MRI and 16.4% parkinsonism including Parkinson's disease. Average 36-month follow-ups; almost all patient suffered from severe cognitive impairment. (b) AD comorbidity; 28%, BD; 17%, and PD; 11%. Biopsy specimen from 2 patients showed BBB breakdown and small vessel disease.

**Conclusions:** Our prospective cohort studies revealed high comorbidity of AD and VD/BD among iNPH patients. Further studies are needed to elucidate the clinical provision against AD, VD/BD and iNPH comorbidity as well as common alterations in CSF drainage and perivascular space among those.

## A83

### Hydrocephalus-shunt-malfunction: tap-test via "pumping" a shunt: its diagnostic relevance. A prospective evaluation

U. Kehler
Asklepios Klinik Altona, Hamburg, Germany
**Correspondence:** U. Kehler
*Fluids and Barriers of the CNS* 2018, **15(Suppl 1)**:A83

**Introduction:** Shunt pumping is controversial; however, no clinical evaluations of high volume tap test via shunt pumping are published. Advantage of "shunt pumping" is the non-invasiveness of the procedure. This prospective study was performed to estimate the value of high volume shunt pumping for further treatment options in patients with suspected shunt malfunction.

**Methods:** We followed 17 patients with NPH who presented months or years after the shunt implantation with slow clinical deterioration. All patients were shunted with a gravitational and an additional "Sprung-reservoir", which is a borehole reservoir with an anti reflux valve. The shunt was pumped during an outpatient visit at least 100 times through pressing down the membrane of the reservoir resulting in release of at least 20 ml CSF. Patients and/or relative got a evaluation sheet where they had to mark the subjective changes (very good, good, marginal/none) of gait, incontinence and the overall feeling as well as the time course of these changes.

**Results:** Pumping the shunt was possible in all cases. In 7 patients a very good improvement after pumping was seen, in 5 a good improvement and in 5 only marginal or no improvement. The improvement was observed in 8 cases within 1 h after pumping, in 4 within 1 and 3 h, and in 2 within 3–6 h. A later improvement didn´t appear. The improvements lasted in all cases more than 3 h. In 3 cases the improvement disappeared before 12 h, in 2 cases it lasted more than 12 h and in 9 more than 48 h.

**Conclusions:** Shunt pumping can detect shunt malfunction fast and non-invasively. When the effect of pumping can be observed and how long it will last differs substantially from patient to patient. Therefore multiple evaluations are necessary after pumping. In patients with temporary improvement shunt revision should be discussed if no lower of the opening pressure is possible. In patient without clinical improvement further (invasive) investigations are recommend before excluding shunt malfunction.

## A84

### Characteristics of diffusion tensor imaging profiles in late stage NPH patients

N. C. Keong[1], J. Kwok[2], S. Tan[3], H. Rumpel[3], S. Kumar[4], E. K. Tan[5], L. L. Chan[3]
[1]Department of Neurosurgery, National Neuroscience Institute and Duke-NUS medical school, Singapore, Singapore; [2]Department of Neurosurgery, National Neuroscience Institute, Singapore, Singapore; [3]Department of Radiology, Singapore General Hospital, Singapore, Singapore; [4]Department of Neuroradiology, National Neuroscience Institute, Singapore, Singapore; [5]Department of Neurology, National Neuroscience Institute and Duke-NUS medical school, Singapore, Singapore
**Correspondence:** N. C. Keong
*Fluids and Barriers of the CNS* 2018, **15(Suppl 1)**:A84

**Introduction:** Studies on iNPH focus on early assessment of probable NPH. Yet, responsiveness to shunting may be imperfect. A challenging Asian clinical subtype, characterized by a history of gait, balance and/or cognitive impairment, with newly-diagnosed ventriculomegaly, presents late for intervention. Such late stage patients have lower functioning abilities and established dementia; responsiveness to CSF drainage is uncertain. We used diffusion tensor imaging (DTI) to characterize white matter injury patterns.

**Method:** 10 patients with late probable NPH underwent a clinical protocol and multi-modal imaging. A full panel of DTI measures, including FA, MD, axial (L1) and radial diffusivities (L2 and L3), was examined. The DTI dataset was interrogated using both region-of-interest and whole brain tractography.

**Results:** 10 participants (6 males, 4 females) were analysed; mean age was 74.60 years. Mean MMSE scores were 22/30 (SD = 8; Md = 22, IQ: 18–28). 40% of participants completed the 10-m walk test. Mean gait score was 63.38 s (SD = 68.40; Md = 37.50, IQ: 17.13–135.50), displaying a walking velocity of .158 m/s. Mean DTI values were: FA = 0.51; MD = 9.50; L1 = 15.35; L2 = 7.67; L3 = 5.50; L2 and 3 = 6.58. MD had significant positive correlation with both axial and radial diffusivities; L1 ($r = 0.843$, $p = 0.002$); L2 ($r = 0.944$, $p < 0.001$); L3 ($r = 0.954$, $p < 0.001$); L2 and 3 ($r = 0.952$, $p < 0.001$).There was a significant positive correlation between age and L1 ($r = 0.664$, $p = 0.036$). DTI profiles demonstrated trends of axonal distortion (high MD) and stretch/compression (high axial diffusivity and tract characteristics), consistent with patterns found in classic iNPH.
**Conclusions:** Assessment of late stage NPH is problematic. DTI may be a useful supplementary tool in identifying white matter injury patterns amenable to intervention.

## A85

### Correlations of diffusion tensor imaging profiles in NPH patients with comorbidities

N. C. Keong[1], J. Kwok[2], A. SL Ng[3], N. Kandiah[4], H. Rumpel[5], L. L. Chan[5], S. Kumar[6]
[1]Department of Neurosurgery, National Neuroscience Institute and Duke-NUS medical school, Singapore, Singapore; [2]Department of Neurosurgery, National Neuroscience Institute, Singapore, Singapore; [3]Department of Neurology, National Neuroscience Institute, Singapore, Singapore; [4]Department of Neurology, National Neuroscience Institute and Duke-NUS medical school, Singapore, Singapore; [5]Department of Radiology, Singapore General Hospital, Singapore, Singapore; [6]Department of Neuroradiology, National Neuroscience Institute, Singapore, Singapore
**Correspondence:** N. C. Keong
*Fluids and Barriers of the CNS* 2018, **15(Suppl 1)**:A85

**Introduction:** NPH patients with comorbidities such as vascular risk and neurodegenerative conditions are poor candidates for surgical intervention. Yet, this subtype represents a challenging clinical unmet need. Representative data on complex NPH patients are currently lacking. We used DTI to examine characteristics of reversible vs. irreversible injury.
**Method:** 8 patients underwent external lumbar drainage, clinical assessment, DTI and fluid biomarkers. Inclusion criteria: participants with possible NPH and significant comorbidities, such as cardiac/vascular burden, higher-level gait disorder with cognitive impairment or neurodegenerative disease.
**Results:** Imaging datasets from 7 participants (5 males, 2 females) were available for analysis; mean age was 72.57 years old. Mean MMSE was 19/30 (SD = 7.49; Md = 15, IQ: 13–27). 85.7% of participants completed the 10-m walk test. Mean gait score was 32.01 s (SD = 25.35; Md = 20.36, IQ: 12.10–63.07), displaying a walking velocity of .312 m/s. Mean DTI values FA = 0.46; MD = 9.76; L1 = 14.95; L2 = 8.44; L3 = 5.86; L2 and 3 = 7.15. FA had a significant negative correlation with radial diffusivities; L2 (r = 0.842, p = 0.18); L3 (r = 0.768, p = 0.044); L2 and 3 (r = 0.812, p = 0.026). MD had a significant positive correlation with both axial and radial diffusivities; L1 (r = 0.940, p = 0.002); L2 (r = 0.947, p = 0.001); L3 (r = 0.982, p < 0.001); L2 and 3 (r = 0.971, p < 0.001). Despite overlay from multiple pathologies, complex NPH demonstrated similar DTI profiles to classic iNPH patients (increase in mean and axial diffusivities).
**Conclusions:** Patients with complex NPH demonstrate less frequent improvement following CSF diversion. However, DTI profiles for improvement were consistent with patterns amenable to intervention seen in classic iNPH. Further cross-correlations with other imaging and fluid biomarkers for vascular, neurodegeneration and traumatic brain injury pathologies would be useful.

## A86

### Change of brain density distribution in CT image after shunting for hydrocephalus: a case study of two pediatric patients with achondroplasia

E. J. Kim[1], H. Kim[1], H. H. Yoo[2], X. Yang[1], J. H. Phi[3], D. J. Kim[1]
[1]Department of Brain and Cognitive Engineering, Korea University, Seoul, South Korea; [2]Department of Biomedical Engineering, Korea University, Seoul, South Korea; [3]Department of Neurosurgery, Seoul National University Hospital, College of Medicine, 101, Daehak-ro Jongno-gu, Seoul, South Korea
**Correspondence:** E. J. Kim
*Fluids and Barriers of the CNS* 2018, **15(Suppl 1)**:A86

**Introduction:** Incidence of shunt revision for hydrocephalus is higher in achondroplasia patients than in general population. A successful shunt insertion and reduction of ventriculomegaly are often assessed via postoperative neuroimaging. This study speculated that a change in brain density could be associated with the efficacy of shunt insertion, and set out to investigate whether such change could be evaluated in non-contrast computed tomography (CT) images of achondroplasia patients.
**Methods:** CT images of two pediatric achondroplasia patients who had undergone shunt surgeries were subjected to analyses. Subject A (male, age = 12) had received the surgery; no further revision was required thus far. Subject B (male, age = 11), after the shunt insertion (age = 19), required repeated revisions. The post-operative CT images were acquired and subjected to the quantitative, densitometric analyses to derive the density distributions across the whole cerebrum. An averaged density distribution was acquired from normal pediatric population to act as a reference, and was compared to those of each achondroplasia patients.
**Results:** The post-operative density distribution of subject A was similar to that of normal pediatric population. On the other hand, the post-operative density distribution of subject B did not change despite the repeated revisions, and had significantly different morphology compared to the reference distribution.
**Conclusions:** The morphology of brain density distribution, acquired via densitometric analyses, could be associated with shunt responsiveness. Quantitative, densitometric analysis upon CT images may hold prognostic value regarding the efficacy of shunt surgery.

## A87

### Worsened intracranial compliance may correlate to worse symptoms in normal pressure hydrocephalus

H. J. Kim[1], H. Kim[1], H. H. Yoo[2], S. B. Lee[1], Z. Czosnyka[3], M. Czosnyka[3], D. J. Kim[1]
[1]Department of Brain and Cognitive Engineering, Korea University, Seoul, South Korea; [2]Department of Biomedical Engineering, Korea University, Seoul, South Korea; [3]Department of Neurosurgery, Addenbrooke's Hospital, University of Cambridge, Cambridge, UK
**Correspondence:** H. J. Kim
*Fluids and Barriers of the CNS* 2018, **15(Suppl 1)**:A87

**Introduction:** A reduced compliance of intracranial entities is a major physiological change in normal pressure hydrocephalus (NPH). Reduced compliances in subarachnoid space, brain parenchyma, arterial and venous systems are suspected to contribute to the development of NPH. However, the relationship between such pathological changes and the worsening of symptoms in NPH is not well understood. This study speculated that a worsened intracranial compliance could be associated with worsened symptoms of NPH, and set out to investigate the relationship via cerebrospinal fluid (CSF) infusion tests.
**Methods:** The clinical information and data derived from infusion tests in a total of 42 patients who presented symptoms of NPH were retrospectively investigated. The intracranial compliance was evaluated by high-frequency centroid (HFC; range = 2.5–4 Hz) obtained by spectral analysis upon the baseline CSF pressure signal during infusion test. Symptom worsening is defined as a full Hakim triad, i.e. gait disturbance, cognitive deterioration, and urinary incontinence. The CSF pressure, pulse amplitude of CSF pressure (AMP) and resistance to CSF outflow (Rcsf) was also obtained, and subjected to the statistical analyses.
**Results:** Four patients presented the full triad, hence considered as having worse symptoms. The HFC was significantly higher in patients with worse symptoms (median = 9.57 Hz) compared to the non-full triad patients (median = 8.80 Hz), indicating decreased intracranial compliance in these patients. The diagnostic capacity for the worse symptoms was best estimated by HFC (AUC = 0.829), followed by AMP (AUC = 0.796), Rcsf (AUC = 0.770).
**Conclusions:** A full Hakim triad is associated with worse intracranial compliance in NPH. The results of this study warrant further investigation on the relationship between symptoms of NPH and intracranial compliance.

## A88

### A hospital-based, prospective study on the natural history of AVIM (asymptomatic ventriculomegaly with features of iNPH on MRI)

L. Kimihira[1], Y. Takahashi[1], C. Iseki[1], H. Sato[1], K. Suzuki[1], H. Kazui[2], N. Kuriyama[3], M. Nakajima[4], M. Miyajima[4], H. Arai[4], T. Kato[1]
[1]Division of Neurology and Clinical Neuroscience, Department of Internal Medicine III, Yamagata University School of Medicine, Yamagata, Japan; [2]Department of Psychiatry, Osaka University Graduate School of Medicine, Osaka, Japan; [3]Department of Epidemiology for Community Health and Medicine, Kyoto Prefectural University of Medicine, Kyoto, Japan; [4]Department of Neurosurgery, Juntendo University School of Medicine, Tokyo, Japan
**Correspondence:** L. Kimihira
*Fluids and Barriers of the CNS* 2018, **15(Suppl 1)**:A88

**Introduction:** Our previous study has demonstrated that a part of subjects with AVIM (asymptomatic ventriculomegaly with features of iNPH on MRI) convert to iNPH. Here we investigated conversion rate and predictors of conversion.

**Methods:** We conducted a prospective study of subjects with AVIM who were collected from several medical institutions/hospitals in Japan. AVIM is defined as the "ventriculomegaly with features of iNPH on MRI without objective symptoms", including subjects with subjective complaints of the iNPH triad (gait disturbance, cognitive impairment and/or urinary incontinence). We measured possible predicting factors for conversion including age, sex, education, exercise, height, weight, blood pressure, diabetes, dyslipidemia, history of mental diseases, head injury, sinusitis, smoking, alcohol, Evans index, and the presence of DESH (Disproportionately Enlarged Subarachnoid-space Hydrocephalus), and analyzed the predictive value by Chi squared test or Mann–Whitney U test. In addition, to assess the association between the number of subjective complaints and AVIM-iNPH conversion, the Cochran-Armitage test for trend was used.

**Results:** In 2012, 93 subjects with AVIM were registered and enrolled to the study. Among them, 45 subjects were followed up for 3 years. 27 subjects were diagnosed as iNPH (11 definite, 6 probable, and 10 possible cases), while the remaining 18 subjects were still asymptomatic in 2015. None of the factors except subjective complaints of iNPH triad at baseline was associated with AVIM-iNPH conversion.

**Conclusions:** Conversion rate from AVIM to iNPH was ~ 20% per year (27/45 per 3 years). Except subjective complaints, demographic, social and medical factors could not predict AVIM-iNPH conversion.

## A89

### A long-term outcome and influence of comorbidities after shunt surgery for idiopathic normal pressure hydrocephalus (INPH): a retrospective cohort study

T. Kimura, A. Tucker, N. Suzuki, S. Yoshida
Department of Neurosurgery, Kitami Red Cross Hospital, Okhotsk Cerebrovascular Center, Kitami, Japan
**Correspondence:** T. Kimura
*Fluids and Barriers of the CNS* 2018, **15(Suppl 1)**:A89

**Introduction:** Based on the observation of our own cases, we examined the long-term prognosis of iNPH and the problems of its comorbidities.

**Methods:** For a period of 10 years between 2005 and 2014, a tap test was conducted on those suspected to have probable iNPH according to the 2004 Guideline for iNPH. Subsequently, 166 cases who showed 10% or more clinical improvement were selected and V–P shunt (on 14 cases) or L–P shunt (on 152 cases) was conducted. The subjects of the current study consisted of 81 patients who were available for a follow-up. Modified Rankin Scale (mRS) was determined before and after, as well as 1, 2, 4, 6, and 8 years after the treatment.

**Results:** The improvement in mRS exceeding 1 was noted in 97.6% after 1 month of treatment and in 85.0% after 1 year: the subsequent improvement was observed in 75, 74.2, 70.6, and 28.6%, after 2, 4, 6, and 8 years, respectively. Currently, an overall improvement in mRS exceeding 1 is noted in 68.8% (among 61 patients excluding those 21 who had expired). Co-morbidities included the following: Alzheimer diseases (27 cases), Parkinson disease (5 cases), benign prostatic hyperplasia (10 cases), fractures (12 cases), knee osteoarthritis (3 cases), chronic articular rheumatism (2 cases), cerebral infarction (10 cases), cerebral hemorrhage (one case), subarachnoid hemorrhage (one case) and cancer (8 cases).

**Conclusions:** It is necessary to consider the possibility that the treatment of iNPH may not produce sufficient results due to unpredictable physiopathological conditions.

## A90

### Intra-operative endoscopic findings of idiopathic normal pressure hydrocephalus

D. Kita[1], Y. Hayashi[2], I. Fukui[1], C. Park[3], Y. Kitamura[1], M. Nakada[2]
[1]Department of Neurosurgery, Yokohama Sakae Kyosai Hospital, Yokohama, Japan; [2]Department of Neurosurgery, Kanazawa University Hospital, Kanazawa, Japan; [3]Department of Neurosurgery, Noto General Hospital, Nanao, Japan
**Correspondence:** D. Kita
*Fluids and Barriers of the CNS* 2018, **15(Suppl 1)**:A90

**Introduction:** Endoscopic examination of the intra-ventricular walls is rarely performed for cases of idiopathic normal pressure hydrocephalus (iNPH) since shunting is the first treatment option for iNPH. We conducted endoscopic inspection during shunt surgery for patients with iNPH and compared the obtained findings with their pre-operative MRI data, and clinical manifestations.

**Methods:** 16 patients (mean 76.6 years) with probable iNPH consistent with the Japanese iNPH guideline were included in this study. High-resolution MRI (T1-3D-SPGR or FIESTA) was taken pre-operatively. Intra-ventricular inspection was performed with an endoscope via a frontal burr hole during ventriculoperitoneal shunt surgery.

**Results:** In the lateral ventricles, laceration of the septum pellucidum was found in 7 patients (44%), which was difficult to be detected by pre-operative MRI. The interspace between the bilateral mammillary bodies varied from being wide to narrow. A significant correlation was found between laceration of the septum pellucidum and the callosal angle measured by MRI. The laceration showed significant correlation with preexisting cerebral infarction and white mater change. The patterns of interspace between the bilateral mammillary bodies were correlated significantly with the width of the third ventricle, while neither significant correlation was seen with degenerative changes in MRI nor pre-operative clinical examinations.

**Conclusions:** In iNPH, the pre-operative MRI findings of dull callosal angle and wide third ventricle were closely related to the intra-operative endoscopic findings of laceration of the septum pellucidum and wide opening between the bilateral mammillary bodies, respectively. Brain degenerative changes may be associated with the laceration of the septum pellucidum.

## A91

### Imaging in INPH, are CT and MRI comparable?

K. Kockum[1], J. Virhammar[2], E. M. Larsson[3], K. Laurell[1]
[1]Department of Pharmacology and Clinical Neuroscience, Unit of Neurology, Östersund, Umeå University, Umeå, Sweden; [2]Department of Neuroscience, Neurology, Uppsala University Hospital, Uppsala, Sweden; [3]Department of Radiology, Uppsala University, Uppsala, Sweden
**Correspondence:** K. Kockum
*Fluids and Barriers of the CNS* 2018, **15(Suppl 1)**:A91

**Introduction:** Imaging is fundamental in diagnosing idiopathic normal pressure hydrocephalus (iNPH), where radiological features serve diagnostical support. The aim was to assess the agreement between computed tomography (CT) and magnetic resonance imaging (MRI) for seven radiological iNPH-associated features.

**Methods:** 354 patients received ventriculoperitoneal shunt for iNPH in Uppsala, Sweden, 2011–2015. Of those, 140 had both preoperative CT and MRI. Inclusion criteria were 1 mm isotropic voxel size or coronal reformats/scans, and scans maximum 3 months apart ($n = 38$). In a pilot study of 27 patients, CT and MRI were assessed for Evans' index, high convexity sulci, focally enlarged sulci, Sylvian fissures, temporal horns, callosal angle and periventricular edema. All scans were blindly assessed twice, with 6 weeks' interval. To calculate the agreement

Intraclass correlation (ICC) was used for continuous variables, Kappa and squared weighted Kappa for dichotomous and three stepped ordinal variables respectively.

**Results:** The agreement between CT and MRI was good to excellent, for Evans' index, temporal horns, callosal angle and Sylvian fissures (Kappa and ICC = 0.7–0.9, p = < 0.001). The concordance was poor for periventricular edema and focally enlarged sulci (ICC = 0.2–0.4, nonsignificant). The intraobserver variability was good to excellent between consecutive CT assessments (Weighed Kappa and ICC = 0.7–0.9, p = < 0.001).

**Conclusions:** Radiological signs associated with iNPH can be equally assessed on CT and MRI, except for periventricular edema, due to the superior soft tissue contrast of MRI. The intraobserver reliability for CT is high.

## A92

### Copy number loss in the intron 2 of the *SFMBT1* gene is associated with idiopathic normal pressure hydrocephalus: a cross-sectional multinational study of 942 INPH patients

V. Korhonen[1], S. Helisalmi[2,3], A. Jokinen[1], I. Jokinen[1], J. M. Lehtola[1], M. Kojoukhova[1], A. M. Koivisto[2,3], T. Rauramaa[4], A. Sutela[5], R. Vanninen[5], A. K. Haapasalo[6], M. Hiltunen[2,3], T. Rauramaa[4], H. Saito[7], T. Kato[7], H. Soininen[2,3], J. E. Jääskeläinen[1], A. M. Remes[2,3], P. K. Eide[8], V. Leinonen[1]
[1]Department of Neurosurgery, University of Eastern Finland and Kuopio University Hospital, Kuopio, Finland; [2]Institute of Clinical Medicine-Neurology, University of Eastern Finland, Kuopio, Finland and [3]Department of Neurology, Kuopio University Hospital, Finland; [4]Department of Pathology, Kuopio University Hospital, Finland; [5]Department of Radiology, Kuopio University Hospital and University of Eastern Finland, Finland; [6]A.I. Virtanen Institute for Molecular Sciences, University of Eastern Finland, Kuopio, Finland; [7]Department of Neurology, Hematology, Metabolism, Endocrinology, and Diabetology, Yamagata University, Japan; [8]Department of Neurosurgery, Oslo University Hospital-Rikshospitalet and University of Oslo, Norway
**Correspondence:** V. Korhonen
*Fluids and Barriers of the CNS* 2018, **15(Suppl 1)**:A92

**Introduction:** The aetiology of iNPH remains unclear. However, increasing evidence indicate a potential genetic component in iNPH. It was recently reported in Japan that the copy number loss in intron 2 of the *SFMBT1* gene is present in 50% of patients, who present concomitantly with clinical features of iNPH together with enlarged ventricles and in 26% of shunted iNPH patients compared to 5% in healthy elderly controls. However, these results were obtained using small cohorts and have not yet been replicated outside of Japan. Interestingly, the SFMBT1 protein has been identified to be present in many structures important for the secretion, circulation, and absorption of CSF.

**Methods:** The copy number loss in intron 2 of the *SFMBT1* gene was detected using quantitative-PCR. The study cohort consisted of 936 (555 Finnish and 387 Norwegian) iNPH patients and 530 neurologically healthy elderly Finnish controls.

**Results:** The prevalence of the copy number loss in the *SFMBT1* was determined to be 11% Finnish iNPH-patients and 21% in Norwegian iNPH-patients compared to 3.7% in Finnish controls.

**Conclusions:** The increased prevalence of copy number loss in intron 2 of the *SFMBT1* in iNPH is now replicated in Finnish and Norwegian cohorts further emphasizing its role in the development of iNPH.

## A93

### Posttraumatic hydrocephalus in patients with severe disorders of consciousness: diagnostic advances and long-term outcomes of surgical treatment

A. Kravchuk, Y. Latyshev, N. Zakharova, O. Zaitsev, L. Likhterman, E. Sharova, S. Abuzaid
N.N. Burdenko National Scientific and Practical Center for Neurosurgery, Moscow, Russian Federation
**Correspondence:** A. Kravchuk
*Fluids and Barriers of the CNS* 2018, **15(Suppl 1)**:A93

**Introduction:** Posttraumatic hydrocephalus (PTH) is a known sequela of severe traumatic brain injury. Mental recovery and rehabilitation of patients are affected and complicated by hydrocephalus. Patients in vegetative (VS) and minimal consciousness state (MCS) pose a challenge for a surgeon to decide whom to treat. The goal of our study is to develop a reliable diagnostic criteria to differentiate PTH and atrophy.

**Methods:** 216 patients with PTH were treated in the Burdenko Research Center. Among them 31 patients were in VS and 46 in MCS. Mean time of surgery after trauma in these groups was 5,6 ± 4,01 months. All patients were assessed according to a standard clinical assessment protocol. A new algorithm based on a complex MRI (cisternography, phase-contrast, DTI and ASL) and clinical study was proposed to distinguish PTH and atrophy. In 210 patients standard shunting procedures were made and 6 endoscopic ventriculostomies were performed in cases of proved obstruction.

**Results:** Positive results were observed in 20 (64.5%) patients in the VS group and 30 (65.8%) patients in the MCS group. Unfavorable outcomes were observed in 11 VS patients and 16 MCS patients with a mortality rate of 9.6 and 4.3% respectively. None of the patients in these groups was treated endoscopically. In one case ventricular catheter tip was placed through the 3rd ventricle and aqueduct into the 4th ventricle with endoscopic assistance. Most of the patients had higher risk of shunt infection due to bedsores (n = 6), tracheostomy (n = 37), gastrostomy (n = 2) or both (n = 10). Clinical course was complicated by shunt infection in 8 cases—in three cases (9.6%) in VS patients and in 5 cases (10.8%) in the second group respectively.

**Conclusions:** CSF diversion can be the necessary part of rehabilitation in patients with brain trauma sequela. However a significant number of unfavorable outcomes and absence of reliable non-invasive diagnostic methods make the clinical problem of posttraumatic hydrocephalus unsolved. A complex clinical and MRI assessment helps to state indications for surgery and sometimes to plan the procedure properly according to the individual anatomy of a patient.

## A94

### Epidemiology and clinical characteristics of iNPH in Japan

N. Kuriyama[1,8], M. Miyajima[2], M. Nakajima[2], M. Kurosawa[3], W. Fukushima[4], Y. Watanabe[1], E. Ozaki[1], Y. Hirota[9], A. Tamakoshi[5], E. Mori[6], T. Kato[7], T. Tokuda[8], A. Urae[10], H. Arai[2]
[1]Department of Epidemiology for Community Health and Medicine, Kyoto Prefectural University of Medicine, Kyoto, Japan; [2]Department of Neurosurgery, Juntendo University Graduate School of Medicine, Tokyo, Japan; [3]Department of Epidemiology and Environmental Health, Juntendo University Graduate School of Medicine, Tokyo, Japan; [4]Department of Public Health, Osaka City University Faculty of Medicine, Osaka, Japan; [5]Department of Public Health, Hokkaido University Graduate School of Medicine, Sapporo, Japan; [6]Department of Behavioral Neurology and Cognitive Neuroscience, Tohoku University Graduate School of Medicine, Sendai, Japan; [7]Department of Neurology, Hematology, Metabolism, Endocrinology and Diabetology, Yamagata University Faculty of Medicine, Yamagata, Japan; [8]Department of Neurology, Kyoto Prefectural University of Medicine, Kyoto, Japan; [9]College of Healthcare Management, Miyama, Japan; [10]Mediscience Planning Inc.,Tokyo, Japan
**Correspondence:** N. Kuriyama
*Fluids and Barriers of the CNS* 2018, **15(Suppl 1)**:A94

**Introduction:** There have been no nationwide epidemiological studies concerning Idiopathic normal pressure hydrocephalus (iNPH) in Japan. We conducted a nationwide hospital-based epidemiologic survey of iNPH to determine the number of cases, clinical characteristics by sex and diagnostic level.

**Methods:** The first survey examined the numbers of cases that met the diagnostic criteria of iNPH and those who underwent shunt operations in 2012. The second survey gathered patients' details to clarify their clinical background characteristics.

**Results:** The estimated number of cases meeting the diagnostic criteria in 2012 was 12,900, with 6700 undergoing shunt operations. The estimated crude prevalence was 10.2/100,000 persons. iNPH occurs most frequently in the 70 s in both men and women. Significantly higher frequencies of gait impairment in men and cognitive decline in women were observed as initial symptoms. At the time of definitive diagnosis, gait impairment was observed most frequently in patients with definite iNPH (77.7%). Hypertension was the most frequent comorbidity (40.0%), followed by diabetes mellitus (17.8%) and Alzheimer's disease (14.8%). Hypertension was observed more frequently in men, but diabetes was observed more frequently in women (p 0.05). An LP shunt was the first-choice (55.1%) treatment of iNPH, followed by a VP shunt (43.2%).
**Conclusions:** The present study showed that iNPH occurs most frequently in the 70 s, gait impairment and cognitive decline are the most frequent initial symptoms in men and women, respectively. Hypertension and diabetes are the most frequent comorbidities in men and women, respectively.

## A95

### Outcome implications of using infusion tests for shunt testing in vivo

A. D. Lalou[1], M. Czosnyka[1], J. Donnelly[1], J. D. Pickard[1], E. Nabbanja[1], P. J. Hutchinson[1], M. Garnett[1], Z. Czosnyka[1]
[1]Department of Clinical Neurosciences, Division of Neurosurgery, University of Cambridge University Hospital, Cambridge, UK;
**Correspondence:** A. D. Lalou
*Fluids and Barriers of the CNS* 2018, **15(Suppl 1)**:A95

**Introduction:** Infusion tests, described and implemented in clinical practice since over 40 years ago, have been a reported as potentially useful diagnostic tool for testing shunts' function in vivo. They allow differentiation of a correctly functioning shunt device from a shunt with possible problems. This is the first study summarising the health and financial benefits derived from their implementation in routine clinical practice.
**Methods:** From January 2013 until December 2015, we identified the results of infusion tests from shunted individuals with hydrocephalus or Idiopathic Intracranial hypertension (IIH). We followed patients up after 6 and 12 months from the test and marked them as improving and non-improving (persisting symptoms or urgent revision).
**Results:** 366 infusion tests had been performed on 281 shunted hydrocephalic patients and 85 on IIH patients. For hydrocephalus patients, more than half of the tests (~ 56%, 157 out of 281) showed no shunt malfunction versus 124 with possible malfunction (ages 4 months to 90 years old, 127 males and 154 females). For IIH patients aged 10–77 years old, 47 had possible problems and 38 no indication for shunt malfunction. Overall, over 280 (289 in 6 months, 281 in 12 months) unnecessary revisions were avoided over 3 years' time. 240 (85%) of those non-surgically managed, remained well long-term and did not require surgery.
**Conclusions:** Our results provide evidence of the importance of testing shunts in vivo to confirm their malfunction. Avoiding unnecessary surgeries bears a strong health benefit for patients that can be translated to a financial benefit for the National Health Service and subsequently other Healthcare systems worldwide.

## A96

### The relationship between cerebral autoregulation, resistance to CSF outflow and outcome in normal pressure hydrocephalus

A. D. Lalou[1], M. Czosnyka[1], J. Donnelly[1], J. D. Pickard[1], E. Nabbanja[1], N. C. Keong[1,2], M. Garnett[1], Z. Czosnyka[1]
[1]Department of Clinical Neurosciences, Division of Neurosurgery, University of Cambridge University Hospital, Cambridge, UK;
[2]Department of Neurosurgery, National Neuroscience Institute and Duke-NUS Medical School, Singapore, Singapore
**Correspondence:** A. D. Lalou
*Fluids and Barriers of the CNS* 2018, **15(Suppl 1)**:A96

**Introduction:** Studies in autoregulation, based on Cerebral Blood Flow (CBF) in Normal Pressure Hydrocephalus (NPH), have so far not proven useful in predicting post-shunting outcome. We examined the relationship between the global autoregulation pressure reactivity index (PRx), the Resistance to CSF outflow (Rout) and their possible effects on outcome after surgery.
**Methods:** We studied a cohort of 131 patients, investigated for possible NPH with CSF infusion tests. PRx was calculated from CSF pressure and arterial pressure recordings. After CSF diversion surgery, patients' outcome after 6 months was marked using a simple scale (improvement, temporary improvement, and no improvement).
**Results:** PRx was negatively correlated with Rout ($R = -0.18$; $p = 0.044$); patients with normal CSF circulation tended to have worse autoregulation. The relationship was stronger for patients qualified by clinical decision for surgery ($N = 83$; $R = -0.28$; $p = 0.03$), and strongest in those who improved after surgery ($N = 64$; $R = -0.36$; $p = 0.03$). In those who did not improve, the correlation was absent ($N = 19$; $R = 0.17$; $p = 0.15$). There was a trend towards higher values for PRx in non-responders than in responders ($PRx = 0.16 \pm 0.04$ vs. $0.09 \pm 0.02$ respectively; $p = 0.061$) and in patients demonstrating higher MAP values ($107.2 \pm 8.2$ in non-responders vs. $89.5 \pm 3.5$ in responders; $p = 0.195$). The product of MAP* (1 + PRx), proposed as a combined measure of arterial hypertension and deranged autoregulation, showed a significant association with outcome (greater value in non-responders; $p = 0.013$).
**Conclusions:** Autoregulation proves to associate with cerebrospinal fluid circulation, and appears strongest in CSF diversion responders. Outcome following surgery is possibly most favourable when Rout is increased and global cerebral autoregulation is intact, in combination with arterial normotension.

## A97

### Falls and fear of falling are common in idiopathic normal pressure hydrocephalus

J. Larsson[1], H. Israelsson[1], A. Eklund[2], J. Malm[1]
[1]Department of Pharmacology and Clinical Neuroscience, Umeå University, Umeå, Sweden; [2]Department of Radiation Sciences, Umeå University, Umeå, Sweden
**Correspondence:** J. Larsson
*Fluids and Barriers of the CNS* 2018, **15(Suppl 1)**:A97

**Introduction:** Gait and balance impairments are predisposing for falls, however, falls in INPH have never been studied. The objective was to investigate falls in INPH before and after shunt surgery, compared to the general population.
**Methods:** Shunted INPH ($n = 176$) were compared to age- and sex-matched population-based controls ($n = 368$). Falls and fear of falling (FoF) were assessed through a questionnaire regarding pre- and postoperative events. Individuals with $\geq 2$ experienced falls were defined as "fallers" and those with $\leq 1$ fall as "non-fallers". FoF was evaluated with a 5-item scale (graded never to always) and the Swedish falls efficacy scale (FES(S)), assessing confidence at avoiding falls in activities of daily living.
**Results:** Comparing INPH and controls, the frequency of fallers was higher in INPH (post- and preoperatively: $p \leq 0.001$). After shunting INPH fell less often ($p < 0.001$) and the frequency of fallers was lower ($p < 0.001$). INPH had more FoF and lower FES(S) than controls pre- and postoperatively and before surgery compared to after (all comparisons: $p < 0.001$). There was no difference in severity of fall-related injuries between INPH and controls.
**Conclusions:** Falls and FoF are overrepresented in INPH. After shunt surgery, INPH feel more confident in avoiding falls and the frequency of fallers is lower. Additional interventions to avoid falls such as home safety improvements and exercise programmes should probably be considered in INPH.

## A98

### Epilepsy, headache and abdominal pain are common in long-term follow-up after csf shunt surgery in INPH

J. Larsson[1], H. Israelsson[1], A. Eklund[2], J. Malm[1]
[1]Department of Pharmacology and Clinical Neuroscience, Umeå University, Umeå, Sweden; [2]Department of Radiation Sciences, Umeå University, Umeå, Sweden
**Correspondence:** J. Larsson
*Fluids and Barriers of the CNS* 2018, **15(Suppl 1)**:A98

**Introduction:** Mild and moderate adverse events (AE) related to shunt surgery in idiopathic normal pressure hydrocephalus (INPH) are common but seldom investigated. The objective was to determine the frequencies of epilepsy, headache and abdominal pain after shunting in INPH.
**Methods:** Consecutively shunted INPH patients (n = 176) were compared to age- and sex-matched population-based controls (n = 368). Epilepsy diagnoses, prescription of antiepileptic drugs (AED) and symptoms of headache and abdominal pain were assessed through national health-care registries and questionnaires [distributed 21 months (range 6–45) after shunting]. An epilepsy diagnosis appearing for the first time after shunting was defined as "new onset epilepsy". To enable comparison between INPH and controls, fictive pre- and post-surgical time periods were constructed for the controls (referred to as "virtual surgery").
**Results:** Epilepsy and AED treatment were more common in shunted INPH than controls (epilepsy: 4.5% vs. 1.1%; p = 0.023, AED: 14.8% vs. 7.3%; p = 0.010). The same patterns were seen for new onset epilepsy and new AED prescriptions (epilepsy: 2.3% vs. 0.0%; p = 0.011, AED: 8.5% vs. 3.3%; p = 0.015). There was no difference between the groups before surgery/virtual surgery. Headache (≥ several times per month) was more common in shunted INPH than controls (36.1% vs. 11.6%; p < 0.001), as was right-sided unilateral headache (p = 0.038). Postural headache was seen in 16% of INPH, and 20% had persistent abdominal pain.
**Conclusions:** Epilepsy, headache and abdominal pain are common in INPH and CSF shunt surgery may be the cause. Clinicians should consider these AEs at postoperative follow-ups and during development of new methods for shunt placement.

## A99

### Arterial spin labeling perfusion mr imaging demonstrates regional CBF decrease in INPH

J. Virhammar[1], K. Laurell[2], A. Ahlgren[3], K. Cesarini[4], E.-M. Larsson[5]
[1]Department of Neuroscience, Neurology, Uppsala University, Uppsala, Sweden; [2]Department of Pharmacology and Clinical Neuroscience, Unit of Neurology, Östersund, Umeå, University, Umeå, Sweden; [3]Department of Medical Radiation Physics, Lund University, Lund, Sweden; [4]Department of Neuroscience, Neurosurgery, Uppsala University, Uppsala, Sweden; [5]Department of Surgical Sciences, Radiology, Uppsala University, Uppsala, Sweden
**Correspondence:** K. Laurell
*Fluids and Barriers of the CNS* 2018, **15(Suppl 1)**:A99

**Introduction:** Regional cerebral blood flow (CBF) has previously been studied in patients with idiopathic normal pressure hydrocephalus (iNPH) with imaging methods that require an intravenous contrast agent or expose the patient to ionizing radiation. The purpose of this study was to assess regional CBF in patients with iNPH compared to healthy controls using the non-invasive quantitative arterial spin labeling MRI technique. A secondary aim was to compare the correlation between symptom severity and CBF.
**Methods:** Differences in regional cerebral perfusion between patients with iNPH and healthy controls were investigated using pseudo-continuous arterial spin labeling perfusion MRI. Twenty-one consecutive patients with iNPH and 21 age- and sex-matched randomly selected healthy controls from the population registry were prospectively included. The controls did not differ from patients with respect to selected vascular risk factors. Twelve different anatomical ROIs were manually drawn on coregistered FLAIR images. Holm-Bonferroni correction was applied to statistical analyses.
**Results:** In iNPH patients, the perfusion was reduced in the periventricular white matter ($p < 0.001$), lentiform nucleus ($p < 0.001$) and thalamus ($p < 0.01$) compared with controls. Reduced cognitive function in patients correlated with lower CBF in the pons (r = 0.71, $p < 0.001$), cerebellum (r = 0.63, p < 0.01) and periventricular white matter (r = 0.60, p < 0.01).
**Conclusions:** Using pseudo-continuous arterial spin labeling, we could confirm findings of a reduced perfusion in the periventricular white matter, basal ganglia and thalamus in patients with iNPH previously observed with other imaging techniques.

## A100

### What can the MMSE tell about cognitive performance in suspected INPH and healthy controls?

O. Lilja-Lund, M. Rosell, J. Andersson, K. Laurell
Unit of Pharmacology and Clinical Neuroscience, Östersund Hospital, Region Jämtland Härjedalen, Sweden
**Correspondence:** O. Lilja-Lund
*Fluids and Barriers of the CNS* 2018, **15(Suppl 1)**:A100

**Introduction:** The total score on the Mini-Mental State Examination (MMSE) is often used as a benchmark for cognitive performance. We compared performance on the MMSE between individuals with unlikely (u-iNPH) and possible/probable iNPH (p-iNPH).
**Methods:** The MMSE, Ray-Auditory-Verbal-Learning-Test (RAVLT), Stroop color-naming (Stroop-C) and interference (Stroop-I) was individually administered.
**Results:** The p-iNPH group (n = 47) had more men (p = 0.004), were older (p = 0.001) and performed poorer on the MMSE-total (m = 25.9 and m = 27.7; p = 0.008), MMSE-Orientation/Time (m = 4.85 and m = 4.98; p = 0.038), MMSE-Orientation/Place (m = 4.43 and m = 4.83; p = 0.023), MMSE-Recall (m = 1.51 and m = 2.18; p = 0.001), RAVLT (m = 24.2 and 33.1; p = 0.001), Stroop-C (m = 88.8 and m = 75; p = 0.019) and Stroop-I (m = 186.4 and 150.4; p = 0.003) than the u-iNPH group (n = 119).
**Conclusions:** The MMSE-total score was significantly lower in the p-iNPH group compared to u-iNPH. Inferior cognitive performance was confirmed with RAVLT and Stroop for the p-iNPH. However, the mean score for the p-iNPH was above the typical cut-off score of 24 points. Also, when comparing performance on each item on the MMSE, only orientation and recall showed any significant difference. Individuals with a total score within the non-demented range might include individuals with specific memory impairments or beginning dementia and caution should be made using the MMSE to ascertain cognitive performance. Implications: Further testing could be needed if individuals only fail the orientation and/or recall task on the MMSE even if the total score is within the non-demented range.

## A101

### Frequency of ventricular dilation and CSF dynamic alterations in mucopolysaccharidososes

A. Lokossou[1], A. Dalla-Corte[2], C. de Souza[3], M. Anés[3], R. Giugliani[2], O. Balédent[1]
[1]University Hospital of Picardie Jules Verne, Medical Image Processing departments, BioFlowImage Laboratory, Amiens, France; [2]Universiadade do Rio Grande do Sul, Porto Alegre, Brazil; [3] Hospital de Clínicas de Porto Alegre, Porto Alegre, Brazil
**Correspondence:** A. Lokossou
*Fluids and Barriers of the CNS* 2018, **15(Suppl 1)**:A101

**Introduction:** Hydrocephalus is characterized by ventricular dilation and cerebrospinal fluid (CSF) dynamic alterations. Mucopolysaccharidoses (MPS) constitute the family of diseases related to an inherited enzymatic storage disorder. Some MPS' subtypes share the same features as hydrocephalus. We studied the frequency of ventricular dilation and CSF dynamic alterations in MPS cohort.
**Methods:** Forty five patients with MPS underwent morphological magnetic resonance imaging (MRI) sequences to evaluate ventricular dilation based on Evan's index > 0.3 and phase contrast MRI to quantify aqueductal CSF stroke volume (**ASV**), cervical CSF stroke volume (**CSV**) using homemade software. Stroke volume (SV) represents the volume of CSF moving through the region of interest in craniocaudally and caudocranially direction during one single heartbeat. Clinical practice on hydrocephalus patients allowed the classification of patients according to their SV. ASV [20; 110] and CSV [200; 800] µl/cc were considered as normal values. MPS patients, who had CSF SVs below or above these intervals, were considered having CSF dynamic alterations.

**Results:** 16 patients presented ventriculomegaly and 14 of them had CSF dynamic alterations. Among the 29 patients without ventriculomegaly, 20 had CSF dynamic alterations. 11 patients presented normal CSF dynamic. At aqueductal level, 3 patients were hyperdynamic and 14 were hypodynamic. At cervical level, 1 patient was hyperdynamic while 27 patients were hypodynamic. The decrease of cervical CSF oscillations in the most patients corroborates with the spinal stenosis reported in the literature.

**Conclusions:** In MPS condition, ventriculomegaly is associated to CSF dynamic alterations. However, CSF dynamic disturbance is frequent in patients without ventriculomegaly.

## A102

### Cerebral venous pulsatility index: a new look at CSF dynamic alterations

A. Lokossou[1], S. Garnotel[1], G. Page[1], L. Balardy[2], Z. Czosnyka[3], P. Payoux[4], O. Balédent[1], E. A. Schmidt [5,6]

[1]University Hospital of Picardie Jules Verne, Medical Image Processing departments-BioFlowImage Laboratory, Amiens, France; [2]Departments of Geriatry, University Hospital Toulouse, Toulouse, France; [3]Neurosciences department, University of Cambridge, Cambridge, UK; [4]Departments of Nuclear Medicine, University Hospital Toulouse, Toulouse, France; [5]UMR 1214-INSERM/UPS-TONIC Toulouse Neuro-Imaging Center, Toulouse, France; [6]Departments of Neurosurgery, University Hospital Toulouse, Toulouse, France

**Correspondence:** A. Lokossou

*Fluids and Barriers of the CNS* 2018, **15(Suppl 1)**:A102

**Introduction:** During the cardiac cycle, kinetic energy is transferred to cranial compartment by **a**rterial **b**lood **f**low (ABF) leading to **v**enous **b**lood **f**low (VBF) drainage and cerebrospinal fluid (CSF) oscillations. We proposed to use phase contrast magnetic resonance imaging (PC-MRI) to determine how arterial and venous pulsatilities were transferred between extracranial and intracranial compartments.

**Methods:** Hydrocephalus patients (n = **54**) underwent PC-MRI to quantify **intracranial and extracranial ABF and VBF** the day before ICP monitoring with infusion tests to assess resistance to CSF outflow (Ro) using ICM+. Based on Ro value, we classified patients in potential-non- responders (**PNR**, n = 22) and potential-responders (**PR**, n = 32) groups to shunt surgery. PC-MRI data were analysed with home-made software to extract **max**imal, **min**imal and **mean** values of: (i) **intracranial** ABF (internal carotid arteries [ICAs] + basilar artery) and VBF (straight + sagittal sinuses); (ii) **extracranial** ABF (ICAs + both vertebral arteries) and VBF (both jugular veins). A pulsatility index **(PI = (max–min)/mean)** was calculated for ABF and VBF for each intracranial and extracranial level.

**Results:** Intracranial and extracranial arterial pulsatilities were not different between the groups. In both groups, intracranial arterial PI was smaller than extracranial arterial PI. Intracranial venous PI was not different between the two groups. The venous PI was increased at extracranial level for both groups and was significantly higher in PR group than in PNR group.

**Conclusions:** Extracranial venous PI was impaired in PR group and seems to be an interesting MRI biomarker highlighting hydrocephalus patients with CSF dynamic alterations and could be helpful to predict shunting candidates.

## A103

### Lumbar puncture and intracranial pressure monitoring in the evaluation of pseudotumor, CSF leak and mixed CSF disorders

M. Luciano[1], L. Manthripragada[1], R. R. Iyer[1], A. Rao[2], A. Moghekar[2], T. Libertini[1], D. Solomon[2]

[1]Department of Neurosurgery, Johns Hopkins Hospital, Baltimore, MD, USA; [2]Department of Neurology, Johns Hopkins Hospital, Baltimore, MD, USA

**Correspondence:** M. Luciano

*Fluids and Barriers of the CNS* 2018, **15(Suppl 1)**:A103

**Introduction**: Lumbar puncture (LP) is the most utilized test in the assessment of neurological disease and disorders of cerebrospinal fluid, but the variability of this single time point measure and the influence of patient position may limit its accuracy and utility. Cranial ICP monitoring (ICPM) has the advantage of direct, continuous intracranial pressure measurement and under various conditions may be considered to offer added utility, albeit through a more invasive approach.

**Methods:** We reviewed the charts of patients undergoing ICPM at our institution between 2015 and 2017. We compared initial LP pressure measurements with subsequent cranial ICPM in patients sub classified as having suspected pseudotumor, intracranial CSF leak, or possible mixed disorders.

**Results:** 73 patients underwent continuous ICPM procedures. Overall mean pressure was 15.5 mm Hg with LP and 11.9 mm Hg with lying ICPM (p = 0.006). Subgroup analysis demonstrated a significant difference between LP and lying ICPM pressures within the pseudotumor cerebri subgroup (p = 0.0004), but no statistical difference between the CSF leak or combined pseudotumor and CSF leak subgroups. The LP and ICPM pressures were not correlated overall (r = 0.36) or within subgroups. A positional difference between lying ICPM and sitting ICPM pressures was statistically significant in both pseudotumor and CSF leak patients (p < 0.0001).

**Conclusions**: LP pressure measurements are limited and vary significantly from continuous ICPM. The positioning during the LP must be considered and position changes with ICPM may add value in accurately conveying pressures in patients with CSF leak, pseudotumor and mixed coexistent processes.

## A104

### Predicting development of Alzheimer's disease in patients with shunted idiopathic normal pressure hydrocephalus

A. J. Luikku[1,2], A. Hall[1], O. Nerg[1,3], A. M. Koivisto[1,3], M. Hiltunen[1,3,4], S. Helisalmi[1], A. Junkkari[2], S. K. Herukka[1,3], A. Sutela[5], M. Kojoukhova[2,5], J. Mattila[6,7], J. Lötjönen[6,7], J. Rummukainen[8], I. Alafuzoff[1,9,10], J. E. Jääskeläinen[2], A. M. Remes[1,3], H. Soininen[1,3], V. Leinonen[2]

[1]Institute of Clinical Medicine-Neurology, University of Eastern Finland, Kuopio, Finland; [2]Neurosurgery of NeuroCenter, Kuopio University Hospital, Kuopio, Finland; [3]Neurology of NeuroCenter, Kuopio University Hospital, Kuopio, Finland; [4]Institute of Biomedicine, University of Eastern Finland, Kuopio, Finland; [5]Department of Radiology, Kuopio University Hospital, Kuopio, Finland; [6]VTT Technical Research Centre of Finland, Tampere, Finland; [7]Combinostics Ltd, Tampere, Finland; [8]Department of Pathology, Kuopio University Hospital, Kuopio, Finland; [9]Department of Immunology, Genetics and Pathology, Rudbeck Laboratory, Uppsala University, Uppsala, Sweden; [10]Department of Pathology and Cytology, Uppsala University Hospital, Uppsala, Sweden

**Correspondence:** A. J. Luikku

*Fluids and Barriers of the CNS* 2018, **15(Suppl 1)**:A104

**Introduction:** Patients with idiopathic normal pressure hydrocephalus (iNPH) have often concomitant Alzheimer's disease (AD) pathology in cortical biopsy samples and later development of clinical AD. Disease State Index (DSI) is a statistical method capable of combining data from various sources to aid clinician in differential diagnosis of dementing diseases and their progression. We evaluated performance of a DSI model aimed to predict development of clinical AD in shunted iNPH patients.

**Methods:** 335 patients initially shunted for iNPH were followed until death or end of June 2015. In total 70 (24.6%) patients developed clinical AD during mean follow-up of 6.2 years (range 0.2–21). DSI model including symptom profile, onset age of NPH symptoms, atrophy of medial temporal lobe in CT/MRI, cortical brain biopsy, and APOE-genotype was applied. Performance was evaluated with Receiver Operating Characteristic (ROC) Curve analysis.

**Results:** DSI model predicted development of clinical AD with moderate power (AUC = 0.75). Most effective measurement groups were cortical biopsy (AUC = 0.67), symptom profile (AUC = 0.66), and atrophy of medial temporal lobe (AUC = 0.66). Applied model had good negative predictive value, as later development of AD could have been ruled out for 54% patients with a NPV of 89.8%.

**Conclusions:** Decision making software can assist in prediction of clinical AD in a subgroup consisting of shunted iNPH patients. Acquiring cortical biopsy during shunt insertion could be justifiable to enhance prediction power of later AD.

## A105

### The eShunt System: development of an endovascular delivery system and device for the treatment of communicating hydrocephalus

A. Malek[1], C. Heilman[2]
[1]Department of Neurosurgery, Tufts Medical Center, Boston, MA, USA; [2]Department of Neurosurgery, Division of Neurovascular Surgery, Tufts Medical Center, Boston, MA, USA
**Correspondence:** C. Heilman
*Fluids and Barriers of the CNS* 2018, **15(Suppl 1)**:A105

**Introduction:** The design and development of a unique endovascular approach; the eShunt System, for the treatment of patients with communicating hydrocephalus is described. Establishing an endovascular treatment using femoral venous access provides an opportunity to develop an implant which mimics the function of the arachnoid granulation, allows treatment of CH patients without open surgery, may eliminate several of the common sources of failure of standard ventriculo-peritoneal shunting systems and greatly simplifies valve design required in standard shunt systems.
**Methods:** 3D printed models, based upon MRI images of the inferior Petrosal sinus, have been used in the development of the eShunt System. Iterative design has allowed testing in bench top models, cadaver specimens and live ovine models.
**Results:** The eShunt System has been successfully deployed in a series bench top, cadaver specimens and live ovine experiments. A First in Human study is contemplated
**Conclusions:** The development of the eShunt System has progressed to a prototype stage that is preparing for First in Human trials. The use of 3D printed, cerebrovascular models based upon patient MRI images has resulted in an endovascular treatment option currently being evaluated in additional live animals in preparation for human trials.

## A106

### 50 ML CSF tap test in INPH: magnitude of response in a placebo arm

J. Malm[1], J. Jacobsson[1], A. Eklund[2], K. Laurell[1]
[1]Department of Pharmacology and Clinical Neuroscience, Umeå University, Umeå, Sweden; [2]Department of Radiation Sciences, Umeå University, Umeå, Sweden
**Correspondence:** J. Malm
*Fluids and Barriers of the CNS* 2018, **15(Suppl 1)**:A106

**Introduction:** The CSF tap test (TT) has been established as simple, safe and inexpensive predictive INPH test. TT is recommended in the international guidelines. We hypothesized a TT placebo response as patient/relatives know that a large volume of CSF is going to be drained and an improvement to be expected. The lp may be painful, and if the test is positive, shunt surgery might be considered. The objective was to determine the magnitude of the placebo response in TT.
**Methods:** 36 patients (15 women, 73 years) diagnosed with "possible" INPH were included. Gait velocity ($6 \times 10$ m) was assessed before and 3 h after the TT. Patients were randomly allocated to a TT (40–50 mL CSF drained) or a sham procedure (lp without puncture). The placebo response was defined as improvement in velocity in the sham group.
**Results:** 17 patients had the sham procedure, and 19 CSF drainage. Before TT, there were no differences in gait velocity between groups. There was a trend, however not significant, that the placebo response was different from zero (0.02 m/s (− 0.05–0.09 95% CI). Commonly used cut offs for a "positive" TT gave the following numbers: improvement $\geq$ 5%, 7 patients; $\geq$ 20%, 2; $\geq$ 0.1 m/s, 5 and $\geq$ 0.15 m/s 3 patients.
**Conclusions:** The placebo response in TT is probably small. The variability of gait velocity before and after the TT may indicate that even sham patients could fulfill criteria for a "positive" TT. A more robust variable, with high test–retest reliability, could probably improve the diagnostic accuracy of the TT.

## A107

### Interaction between intracranial pressure and the sympathetic nervous system; a new link between the brain and the cardiovascular system

S. C. Malpas[1,2], S. J. Guild[1,2], R. Ramchandra[1,2]
[1]University of Auckland, Auckland, New Zealand; [2]Millar Inc, Housten, TX, USA
**Correspondence:** S. C. Malpas
*Fluids and Barriers of the CNS* 2018, **15(Suppl 1)**:A107

**Introduction:** We hypothesize that cerebral perfusion pressure [measured as intracranial pressure (ICP)] is a key regulator of sympathetic nerve activity (SNA). Specifically that small changes in ICP exert a reflex control over SNA.
**Methods:** We instrumented sheep to record renal SNA, ICP and arterial pressure. In the conscious condition a minimum of 4 days after surgery we increased ICP via infusion of artificial CSF into the lateral ventricle in a step wise fashion. The ICP infusions were repeated again after 2 h of ganglionic blocker hexamethonium.
**Results:** The increase in ICP led to a reflex linear increase in arterial pressure even when ICP remained within normal physiological levels (0–20 mmHg) ($n = 6$, $p < 0.01$). For example: a 10 mmHg increase in ICP lead to a $6.5 \pm 1.4$ mmHg ($n = 6$) increase in arterial pressure. Ganglionic blockade significantly reduced or abolished this increase in arterial pressure, suggesting mediation by increased sympathetic nerve activity. This is supported by direct renal sympathetic nerve recordings.
**Conclusions:** This data is supportive of the "selfish brain" hypothesis which proposes that poor brain perfusion is compensated for by chronically increased SNA and arterial pressure. Our data specifically indicate that around resting levels of ICP i.e. not markedly elevated as in the Cushing's reflex, there is a reflex control over SNA which in turn affects arterial pressure.

## A108

### A comparative study of cerebrospinal fluid drainage by tap test versus external lumbar drainage to predict CSF diversion procedure

L. Manthriprgada, R. Sharma, A. R. Moghekar, M. G. Luciano, A. S. Rao
The Johns Hopkins Hospital, Baltimore, MD, USA
**Correspondence:** L. Manthriprgada
*Fluids and Barriers of the CNS* 2018, **15(Suppl 1)**:A108

**Introduction:** Idiopathic normal pressure hydrocephalus (iNPH) is a condition characterized by increased cerebrospinal fluid (CSF) volume commonly seen in elderly patients. The incidence has been reported at 1.8 cases per 100,000 and 2.2 cases per 1,000,000 individuals. Patients present with the classic triad of gait, cognitive and urinary complaints. Imaging shows ventriculomegaly as measured by Evan's index. CSF diversion procedures including shunt and endoscopic third ventriculostomy (ETV) are modalities commonly used to treat iNPH. It is important to be able to predict patient response before undergoing any of the above procedures as they are associated with risks. Many institutions utilize the lumbar puncture or CSF tap test where a large volume (40–50 cc) of CSF is drained ot the lumbar drainage trial using external lumbar catheter and draining up to 300 cc of CSF. Pre and post drainage testing includes tests of cognition and gait. The percentage improvement is calculated to determine if shunt or ETV is indicated. In our institution, the parameters tested for cognitive function are Montreal Cognitive assessment (MOCA) and Digit substitution test (DST). Gait is assessed using 10 m walk, Tinetti, Timed up and go (TUG), Dual TUG, Mini Best. Previous studies have determined the CSF tap test to be sensitive in predicting shunt response (5), while the more invasive extended lumbar drainage test is considered to have higher sensitivity and specificity (6). The aim of this study is to determine which specific parameters in the post drainage cognitive and gait assessments are most predictive of selection for shunt or other CSF diversion procedures.
**Methods:** A retrospective review of patients admitted to The Johns Hopkins CSF disorders center for evaluation iNPH between December 2015 through May 2017 was conducted. Patients who underwent a CSF tap test and extended lumbar CSF drainage were selected for analysis. Pre and post drainage tests (Independent variables) used in the analysis were as follows: Cognitive testing—MOCA, DST. Gait testing—10 m walk, Tinetti, TUG, Dual TUG, 6 min walk, MiniBEST. The dependent variable being a CSF diversion procedure—shunt or ETV.

**Results:** Data from 31 patients was analyzed. 39% were male, 61% were female. 93.5% presented with gait dysfunction, 74.2% with cognitive complaints and 48.4% with urinary symptoms. 93.5% had ventriculomegaly (Evans index of > 3.0). In the CSF tap test group none of the patients obtained a CSF diversion procedure and none of parameters tested were statistically significant. In the External lumbar drainage group there was a statistically significant association with CSF diversion procedure (p 0.059). The parameters that were most predictive of CSF diversion procedure were 10 m walk (p 0.0039), TUG (p 0.0073) and Dual TUG (p 0.0046).

**Conclusions:** Patients who underwent external lumbar drainage trial were more likely to obtain a CSF diversion procedure. Of the pre and post drainage tests performed, the 10 m walk, TUG and Dual TUG are most predictive of obtaining a CSF diversion procedure. Idiopathic normal pressure hydrocephalus (iNPH) is a condition characterized by increased cerebrospinal fluid (CSF) volume commonly seen in elderly patients. CSF diversion procedures including shunt and endoscopic third ventriculostomy (ETV) are modalities commonly used to treat iNPH.

## A109

### Efficacy of lumbo-peritoneal shunt in elderly patients with idiopathic normal pressure hydrocephalus

X. Fang[1], X. Gao[1], W. Shu[1], S. Li[3], W. He[3], G. Liu[2], R. Mao[1]
[1]Neurosurgical Department of Huadong Hospital, Fudan University, Shanghai, China; [2]Neurological Department of Huadong Hospital, Fudan University, Shanghai, China; [3]Radiological Department of Huadong Hospital, Fudan University, Shanghai, China
**Correspondence:** R. Mao
*Fluids and Barriers of the CNS* 2018, **15(Suppl 1)**:A109

**Introduction:** To summarize and evaluate the efficacy and analyze complications of lumbo-peritoneal shunt (LPS) surgery of 16 cases from February 2016 to February 2017 diagnosed of idiopathic normal pressure hydrocephalus in our department. Hope elderly patients with iNPH have more choices in choosing the surgical procedure.

**Methods:** Clinical data of 16 iNPH patients treated from February 2016 to February 2017 who underwent LPS were prospectively gathered in the Neurosurgical Department of Huadong Hospital, Fudan University. Demographic characteristics, including age, gender, clinical symptoms, opening pressure, protein containing, neuro-imaging, pre- and post-operative outcome scale scores (modified Rankin Scale (mRS) and Mini-Mental State Examination (MMSE) score) and complications were assessed. Evan's index before and after operation were also assessed. Gait disturbance and urinary incontinence were evaluated by 10-m walking test and questionnaire respectively. Cerebrospinal fluid (CSF) tap test were done in all patients. 30 ml CSF was removed during tap test. Opening pressure recorded and weight of protein contain examined. Positive tap test was a prerequisite for LPS. Operation (LPS) was done according to standard surgical procedures and a SOPHYSA programmable valve was used in all patients. Initial pressure of the valve was set according to the opening pressure of lumbar puncture. With opening pressure of 70–150 $mmH_2O$ set the valve to 110 $mmH_2O$, 150–200 $mmH_2O$ set to 150 $mmH_2O$. The anesthesia with endo-tracheal intubation was done for the operation. Patients were typically placed in a lateral decubitus position. L3–4 puncture with fine needle guiding was done and the proximal tube was inserted. Distal shunt was placed in the reverse McBurney's point of left lower abdomen. Postoperative follow-up was done on a regular basis, a minimum follow-up of 3 months.

**Results:** There were 16 cases of iNPH, of whom 9 (56%) were male and 7 (44%) were female. Aged from 60 to 89 years, and the mean age was 72.9 ± 7.8 years. Disease course: 18–120 months. Clinical manifestations and disease history: All 16 cases with triad symptoms. 12 (75%) of them have a history of head trauma and 6 (37.5%) of them different bone fractures due to impaired gait disturbance. 13 (81%) of them have a history of AD diagnosis and 5 (31%) of them PD diagnosis. Imaging findings: All 16 cases with head CT and/or MRI showed ventricular enlargement in a different degree. Evans index ≥ 0.3. Coronary plane of MRI display DESH in 12 cases. Reported rate of ventricular enlargement by radiological department was only 25%, in contrast, the reported rate of brain atrophy was 100%. The mean opening pressure was 145 ± 26 (Min = 90, Max = 180) mmH2O. Of them, 10 cases (62.5%) were between 70–150mmH2O and 6 cases (37.5%) were 150–200mmH2O. The mean protein containing of CSF was 0.46 ± 0.23 (Min = 0.17, Max = 1.08) g/L, a little higher than the upper normal limitation (normal: 0.15–0.45 g/L) and glucose 4.2 ± 1.2 (Min = 2.8, Max = 6.5) mmol/L. After CSF tap test, positive results of 10-m walking test were observed within 24 h in 16 cases and within 24–72 h in 14, still effective more than 72 h in 11, without effective case more than a week. The mean MMSE scores before and after Tap test and 3 months after shunt were 14.4 ± 8.4 (Min = 3, Max = 25), 18.1 ± 7.5 (Min = 6, Max = 27), and 22.2 ± 5.4 (Min = 8, Max = 28) respectively, and the difference of post-tap test MMSE and 3-month post-operation to the initial was significant ($p < 0.05$). The mean mRS before and after Tap test and 3 months after shunt were 3.69 ± 0.95 (Min = 2, Max = 5), 2.75 ± 0.86 (Min = 1, Max = 4), and 1.63 ± 1.26 (Min = 0, Max = 4) respectively, and the difference of post-tap test mRS and 3-month post-operation to the initial was significant ($p < 0.05$). The mean Evan's index before and 3 months after shunt was 0.33 ± 0.03 and 0.32 ± 0.03 respectively, the difference was significant ($p < 0.05$). During a follow-up period of 3–15 months after shunt, clinical systems improved in different degrees in all the patients. There were no operation related death and severe complications such as postoperative CNS infection, obstruction of shunt tube, over-drainage and subdural hematoma and etc. Other complications: 1 case of suspected unsmooth diversion of CSF, we continue to observe and will adjust the apparatus if the patient's symptoms progressively deteriorate. Another case of persist pain in lumbar-sacral area, partial remitted after pulling out a bit of tube that was inserted into the subarachnoid space.

**Conclusions:** The results of these cases show that elderly patients with idiopathic normal pressure hydrocephalus can benefit from shunt surgery in terms of their triad symptoms and functional status. LPS is an effective procedure for iNPH with the advantages of minimal invasiveness, rapid recovery, and especially fewer and less severe complications.

## A110

### Neurochi®: a virtual reality and in vitro model of the CSF system for teaching and research

L. R. Sass[1], G. C. Natividad[1], B. Cleveley[2], M. Khani[1], T. Xing[3], O. Baledent[4], B. A. Martin[1]
[1]Biological Engineering, University of Idaho, Moscow, ID, USA; [2]Virtual Technology and Design, University of Idaho, Moscow, ID, USA; [3]Mechanical Engineering, University of Idaho, Moscow, ID, USA; [4]BioFlow Image, University of Picardy Jules Verne, Amiens, France
**Correspondence:** B. A. Martin
*Fluids and Barriers of the CNS* 2018, **15(Suppl 1)**:A110

**Introduction:** CSF is a clear, water-like fluid that surrounds the CNS and fills a complex network of ventricles and cisterns. There is a rapidly growing number of medical applications that integrate virtual and/or augmented reality (VR/AR) systems, most often as a rehabilitation tool and less often for immersive visualization. Due to its complexity, the CSF system is an excellent candidate for immersive visualization as both a teaching tool and a simulator for neurosurgical procedures.

**Methods:** Gross anatomy of the complete CSF system was segmented from high-resolution T2-weighted MRI data. To optimize this geometry for VR systems, extensive remeshing was performed to create a low poly mesh. Fine anatomy was added though a manual process informed by additional segmentations and cadaveric measurements in the literature. The complete anatomic mesh was integrated into a 3D environment where navigational controls were added. Finally, the complete system was exported in a real-time VR platform.

**Results:** The primary VR simulator consists of the ventricular and cortical regions of the brain, dura matter, spinal cord, coccygeal nerve and 30 dorsal and ventral nerve rootlet pairs. The secondary component of the simulator is a real-time anatomical index with a 3D orientation gimbal and a MRI viewport.
**Conclusions:** This project represents the first VR simulator of the CSF system, Neurochi® that is equipped with navigational viewports and indices creating an immersive tool for learning and medical applications. Future work is aimed at improving navigational controls and development of a tactile interface for clinical procedure simulation, http://www.neurochi.com.

## A111

### Quantitative assessment of intrathecal cerebrospinal fluid dynamics and geometry across large mammalian species

L. Sass[1], M. Khani[1], C. Gibbs[1], T. Freeman[1], J. Pluid[1], A. Elliott[1], J. N. Oshinski[2], J. Zeller[3], G. R. Stewart[4], D. Powell[5], B. Petersen[6], D. Weeks[7], G. Carter[7], B. A. Martin[1]
[1]Biological Engineering, University of Idaho, Moscow, ID, USA; [2]Department of Radiology, Emory University, Atlanta, GA, USA; [3]Northern Biomedical Research, Spring Lake, MI, USA; [4]Pairnomix, LLC, Maple Grove, MN, USA; [5]Biomedical Engineering, Univ. of Kentucky, Lexington, KY, USA; [6]Inland Imaging, Spokane, WA, USA; [7]St. Luke's Rehabilitation Institute, Spokane, WA, USA
**Correspondence:** B. A. Martin
*Fluids and Barriers of the CNS* 2018, **15(Suppl 1)**:A111

**Introduction:** Despite the attractiveness of intrathecal drug delivery to the central nervous system (CNS), there is little information about cerebrospinal fluid (CSF) dynamics in humans with CNS disease or animal models used for preclinical research. This hinders development and optimization of intrathecal drug delivery systems. The purpose of this study was to better characterize CSF fluid dynamics in large mammalian species, including humans.
**Methods:** Full-spine high-resolution sagittal T2-weighted magnetic resonance imaging (MRI) measurements were collected for cynomolgus monkeys (n = 8), rhesus monkeys (n = 2), Göttingen minipigs (n = 2) and adult humans (n = 10). Manual segmentation was employed to map the intrathecal geometry, which was quantified in terms of axial distribution of hydraulic diameter, wetted perimeter, cross-sectional area and total volume. CSF dynamics were quantified at 6-axial levels along the spine (foramen magnum, C2/3, C5/6, T4/5, T11/12, L3/4) using phase-contrast MRI measurements with retrospective cardiac gating. Axial distribution of CSF dynamics was quantified in terms of Reynolds and Womersley number, peak value of the mean CSF velocity, peak flow rate and stroke volume.
**Results:** CSF dynamics in minipigs bore no relationship to humans. However, axial distribution of Reynolds number in cynomolgus and rhesus monkeys showed a similar trends to humans, although at approximately half the magnitude.
**Conclusions:** A non-invasive method was developed to quantify CSF dynamics and geometry in large mammalian species. Correspondence to human intrathecal CSF dynamics was poor in minipig and more favorable in non-human primates. Further studies are needed to confirm these findings in a larger population and understand how CSF dynamics alterations impact intrathecal solute distribution.

## A112

### Defining treatment options for patients with complex CSF disorders manifesting as mixed features of intracranial hypotension and hypertension

M. L. Martinez[1], A. E. Mekabaty[1], M. Luciano[2], A. Moghekar[3], F. Hui[1]
[1]Department of Interventional Neuroradiology, Johns Hopkins University School of Medicine, Baltimore, MD, USA; [2]Department of Neurology, Johns Hopkins University School of Medicine, Baltimore, MD, USA; [3]Department of Neurosurgery, Johns Hopkins University School of Medicine, Baltimore, MD, USA
**Correspondence:** M. L. Martinez
*Fluids and Barriers of the CNS* 2018, **15(Suppl 1)**:A112

**Introduction:** Cerebrospinal fluid (CSF) disorders remain a poorly understood entity. Patients with positional headaches that worsen upon standing are often categorized as having intracranial hypotension caused by a CSF leak. Subsequent work-up ensues and treatment through epidural blood "patching" results in improvement of the initial symptoms but can trigger intracranial hypertension. We present 5 patients with positional headaches suggestive of CSF hypotension but with high CSF opening pressures which eventually lead to the diagnosis of idiopathic intracranial hypertension treated by CSF diversion or venous sinus stenting resulting in control of their symptoms.
**Methods:** Institutional CSF disorder database was queried and patients with clinically suspected CSF leak and CSF opening pressure greater than 15 were identified. Demographics, clinical course and imaging were reviewed, as well as method of "definitive treatment".
**Results:** Five patients were identified with discordant CSF pressure measurements (3 males and 2 female). Average age was 42 years (range 34–48). Lumbar puncture opening pressure ranged from 17 to 47 cm $H_20$. The patients were treated with venous stenting, shunting or medically managed with Diamox.
**Conclusions:** Patients with clinically suspected spontaneous CSF leaks presenting with high opening pressure are difficult to manage. A subset of this population may harbour underlying CSF hypertension that would cause weakening of the thecal sac, creating dural diverticula, as well spontaneous CSF leaks. Thus these patients would likely benefit from patching in addition to either CSF diversion or venous sinus stenting. Further studies to elucidate the biomechanics and pathophysiology of CSF leaks are necessary.

## A113

### Water turnover in brain, ventricles and subarachoid spaces in normal volunteers and patients with idiopathic NPH: dynamic pet study using $H_2^{15}O$

M. Mase[1], E. Hayashi[2], H. Yamada[1], N. Oshima[1], K. Aoyama[1], S. Hibino[2], Y. Ito[2], A. Iida[2], T. Miyati[3], E. Mori[4]
[1]Department of Neurosurgery, Nagoya City University Graduate School of Medical Sciences, Nagoya, Japan; [2]Department of Radiology, Nagoya City Rehabilitation Center, Nagoya, Japan; [3]Faculty of Health Science, Institute of Medical, Pharmaceutical and Health Sciences, Kanazawa University, Kanazawa, Japan; [4]Department of Behavioral Neurology and Cognitive Neuroscience, Tohoku University School of Medicine, Sendai, Japan
**Correspondence:** M. Mase
*Fluids and Barriers of the CNS* 2018, **15(Suppl 1)**:A113

**Introduction:** Many studies are ongoing to reconsider cerebrospinal fluid (CSF) physiology including its formation and absorption by using tracers. However, the movement of the tracer is not identical to the movement of water. In order to clarify the origin and turnover of water molecules in CSF, dynamic PET (positron emission tomography) study was performed using radio labeled $H_2O$.
**Methods:** Normal volunteers (n = 9, 62 ± 7.6 years) and patients with definite idiopathic normal pressure hydrocephalus (iNPH, n = 2) were included. Dynamic PET data were obtained for 15 min after intravenous bolus injection of 5 ml of saline including $H_2^{15}O$ (500 MBq). Voxels of interest (VOI) were set in the internal carotid artery (ICA), superior sagittal sinus (SSS), choroid plexus (CP), cortical gray matter (GM), white matter (WM), basal ganglia (BG), lateral ventricle (LV), Sylvian fissure (FS), and prepontine cistern (PPC) based on MR T1 (3D) images with manual corrections. The time and relative radio activity (RAA) curves of each VOI were analyzed.
**Results:** The maximum peak radio activities of GM, WM and BG were at 22.5, 50.0 and 22.5 s after the peak in ICA, respectively. At that time, the relative peak activities of GM, WM and BG were 53, 42 and 55% of the ICA peak activity, respectively. The activities in the whole brain structures decreased gradually. On the contrary, the activity of LV increased gradually until the end of the measurement {14.5% of the ICA peak activity, 49% of the whole brain parenchyma (GM + WM + BG) activity at 12 min}. The activity curve of FS and PPC showed similar to LV. The activity curve of CP was nearly parallel and the level was 80–90% of whole brain parenchyma. The RRA of LV in iNPH tended to be lower compared to the normal control.
**Conclusions:** The present study showed very fast movement of water molecules from artery to brain parenchyma and ventricular and subarachnoid CSF. Water movement into the ventricle may delay in iNPH patients.

## A114

### A new imaging technique for visualization of cerebrospinal fluid motion using dynamic improved motion sensitized driven equilibrium steady-state free precession sequence

M. Matsumae, T. Horie
Tokai University Hospital, Tokyo, Japan
**Correspondence:** M. Matsumae
*Fluids and Barriers of the CNS* 2018, **15(Suppl 1)**:A114

**Introduction:** Currently, observation of cerebrospinal fluid (CSF) motion requires a long acquisition time and complex post-processing of images, depends on the cardiac or respiration cycle, and can only observe CSF motion in a limited area. Here, we propose a novel imaging method, dynamic improved motion sensitized driven equilibrium steady-state free precession (dynamic iMSDE SSFP), which solves these problems.
**Methods:** We investigated the usefulness of dynamic iMSDE SSFP in five healthy volunteers and two patients who underwent surgery to improve CSF motion. Images were acquired with magnetic resonance imaging. Images without iMSDE application were subtracted from the images with iMSDE application to emphasize CSF motions, especially swirl and pulsatile motions.
**Results:** Using dynamic iMSDE SSFP in healthy volunteers, strong CSF motion within the third ventricle was observed, and this motion diffused to the adjacent lateral ventricle and fourth ventricle. CSF motion was also observed in the spinal canal. CSF swirl motion was more limited in the lateral ventricle and the convexity of the supratentorial subarachnoid space. In patients, dynamic iMSDE SSFP demonstrated successful surgical correction of CSF communication.
**Conclusions:** Dynamic iMSDE SSFP validated the concept of CSF motion proposed in recent MRI studies, similar to the phase contrast method and time-spatial labeling pulse method. Furthermore, improved CSF communication after surgery was clearly visualized, validating the clinical usefulness of this technique. Thus, dynamic iMSDE SSFP solves the problems of current methods for imaging CSF motion.

## A115

### ADAM10-mediated junctional neurobiology in the pathogenesis of post-hemorrhagic hydrocephalus

L. Castaneyra Ruiz[1], J. P. McAllister[1], D. M. Morales[1], J. Xu[2], S. L. Brody[2], J. Bubeck-Wardenburg[3], D. D. Limbrick[1]
[1]Department of Neurosurgery, Division of Pediatric Neurosurgery, Washington University School of Medicine, St. Louis, MO, USA; [2]Department of Medicine, Division of Pulmonary and Critical Care Medicine, Washington University School of Medicine, St. Louis, MO, USA; [3]Department of Pediatrics, Division of Pediatric Critical Care, Washington University School of Medicine, St. Louis, MO, USA
**Correspondence:** J. P. McAllister
*Fluids and Barriers of the CNS* 2018, **15(Suppl 1)**:A115

**Introduction:** Post-hemorrhagic hydrocephalus (PHH) develops in approximately 20% of infants with severe intraventricular hemorrhage (IVH), indicating that critical selective mechanistic triggers downstream of the hemorrhage are required for development of this disorder. However, the specific effect of blood on the ventricular zone (VZ) is unknown. Most types of fetal-onset hydrocephalus present with loss of VZ adherens junctions formed by N-cadherin, which leads to disconnection of cells lining the ventricles. ADAM10 is a widely expressed zinc metalloprotease that principally regulates cellular adhesion and migration. In brain, ADAM10-mediated proteolysis of the N-cadherin extracellular domain disrupts cadherin-dependent homotypic intercellular interactions. We hypothesized that hyperactivity of ADAM10 is one of the underlying mechanisms causing disruption of the VZ in PHH.
**Methods:** Newborn mouse brains were dissected and ependymal progenitor cells from the ventricular wall dissociated and plated onto coverslips where they start to differentiate as a monolayer of multiciliated ependymal cells. Five days later, three 2-h treatments were applied: (1) 25µl of blood, (2) 20 hemolytic units of α-hemolysin (an activator of ADAM10), (3) GI254023X (an inhibitor of ADAM10).
**Results:** Blood treatments caused significant reductions in the percentage of multiciliated ependymal cells and expression of N-cadherin, significant over-expression of ADAM10, and decreased transepithelial resistance, suggesting a defect in adherens junctions. Exposure to α-hemolysin caused similar significant decreases in N-cadherin expression. Ongoing experiments with the ADAM10 inhibitor will be presented.
**Conclusions:** These preliminary findings suggest that over-expression of ADAM10 may mediate cleavage of N-cadherin in PHH VZ disruption.

## A116

### The pathogenesis of post-hemorrhagic hydrocephalus in infant ferrets

J. P. McAllister[1], L. Castaneyra Ruiz[1], X. Ge[2], J. Engelbach[2], D. M. Morales[1], B. Baksh, J. Garbow[2], D. D. Limbrick[1]
[1]Department of Neurosurgery, Division of Pediatric Neurosurgery, Washington University School of Medicine, St. Louis, MO, USA; [2]Department of Radiology, Mallinckrodt Institute of Radiology, Washington University School of Medicine, St. Louis, MO, USA; [3]Division of Comparative Medicine, Washington University School of Medicine, St. Louis, MO, USA
**Correspondence:** J. P. McAllister
*Fluids and Barriers of the CNS* 2018, **15(Suppl 1)**:A116

**Introduction:** The pathogenesis of post-hemorrhagic hydrocephalus (PHH) is not clear. While traditional views attribute ventriculomegaly to thrombus obstruction of cerebrospinal fluid (CSF) outflow pathways, recent studies in lissencephalic animals and humans have implicated ventricular zone (VZ) disruption as a prominent mechanism. Nevertheless, systematic studies of PHH are lacking in young animals with a gyrencephalic cortex. To test the hypothesis that VZ disruption occurs following intraventricular hemorrhage (IVH), we have analyzed tissue and CSF from our recently developed infant ferret model of PHH.
**Methods:** PHH was induced in 14–20 day old ferrets by intraventricular injections of lysophosphatidic acid (LPA) or autologous blood; sham controls received similar injections of sterile saline. Neuroimaging (T2-weighted MRI and diffusion tensor imaging) was conducted biweekly until approximately 150 days following induction. Cisternal CSF and fixed tissue from frontal and parietal regions were analyzed using protein chemistry and immunohistochemistry for neuroepithelial/ependymal cells, neural progenitors, multiciliated ependymal cells, astrocytes, cell-adhesion molecules and one of their neuromodulators (ADAM10).
**Results:** Within 1–2 weeks after either LPA or blood injections, mild-moderate ventriculomegaly was confined to the lateral ventricles and was most prominent in the occipital and inferior horns. PHH animals all presented with patches of denuded ependyma, VZ cells lacking cilia and radial processes, eruptions of VZ regions into the ventricle, altered cell-adhesion molecules, increased ADAM10 expression, and reactive astrocytosis in regions of VZ disruption.
**Conclusions:** Since these findings are similar to those observed in patients with PHH, VZ disruption may play an important role in the pathogenesis of this disorder.

## A117

### Thirty years of Aquaporin-1: A Systematic Review Of Aquaporin-1's Role In CSF dynamics

S. Mediratta, C. L. Craven, S. Thompson, L. D'Antona, J. Funnell, L. T. Thorne, A. K. Toma, L. D. Watkins
Victor Horsley Department of Neurosurgery, National Hospital for Neurology and Neurosurgery, London, UK
**Correspondence:** S. Mediratta
*Fluids and Barriers of the CNS* 2018, **15(Suppl 1)**:A117

**Introduction:** Aquaporin-1 and 4 (AQP1 and 4) are protein channels believed to play an important role in water transport within the nervous system. Whilst much is known about AQP4, the role of AQP1 is less well understood. We aim to elucidate the role of AQP1 in cerebrospinal fluid (CSF) dynamics and hydrocephalus through a systematic review of current literature.

**Methods:** MEDLINE (PubMed) search using terms AQP1 or AQP-1 or AQP 1 or AQP one and all non-abbreviated equivalents. Results were filtered to include neurological functions, CSF dynamics, hydrocephalus, intracranial pressure (ICP) and acetazolamide.
**Results:** The search criteria identified 3475 papers, of which 212 studies were relating to neurological function, of which 24 implicated AQP1 to have a role in CSF dynamics and/or intracranial pressure.

| | Papers (n) | AQP1 expression | Function / hypothesis | Organism |
|---|---|---|---|---|
| CSF Production and Hydrocephalus | 11 | Upregulated | CSF production associated with APQ1 expression (vice-versa) | Mice, rat, human cases (NPH) |
| ICP | 4 | Upregulated | Increases with CSF production | Mice, rats (inc. obese model IIH rats) |
| Compensated Hydrocephalus | 6 | Downregulated | Adaptive/ Protective mechanism | Mice, rat, human (paediatric) |
| Acetazolamide | 3 | Inhibited | Acetazolamide inhibited AQP1 protein expression | Mice, rats |

**Conclusions:** There is a growing body of evidence that AQP1 plays a significant role in ICP and CSF dynamics. The evidence suggests that increased AQP1 expression may result in increased CSF production, which may in-turn result in raised ICP. AQP1 inhibition or reduced expression appears to reduce CSF production. Therefore, AQP1 may be a putative target for non-surgical management of disorders of CSF dynamics.

## A118

### A new device for non-invasive flow adjustment

Jan Mügel
GmbH&Co.KG, Potsdam, Germany
**Correspondence:** Jan Mügel
*Fluids and Barriers of the CNS* 2018, **15(Suppl 1)**:A118

**Introduction:** Non-invasive adjustment of the valve characteristic has been one of the important achievements during the last 25 years of valve technology. The focus of these achievements has been the adjustability of the operating pressure. The clinical benefit of flow reducing devices has been limited amongst other things because of its missing adjustability. However, depending on the compliance of the individual patient the adjustment of the flow could additionally have a positive impact on the patients' outcome.
**Methods:** Computer aided designing (CAD) is the method of choice for the construction of a device. The repeated considerations of possible improvements lead to a construction that allows the manufacturing of a prototype. The materials used are titanium, sapphire and neodymium magnets. Parts for the prototypes are manufactured on conventional and computer controlled lathes or milling machines. The prototypes are tested in an apparatus simulating the conditions of an implanted shunt and hereby allowing the investigation of the function of the device.
**Results:** The adjustment of the flow is achieved by a needle determines the opening area in the outlet. The cam plate is moved similar to the mechanism that is used in adjustable differential pressure hydrocephalus valves. An adjustment between an unrestricted flow down to a flow between 5 and 10 ml per hour and any flow in between these values is possible. The pathway through the shunt can be completely closed tentatively by an independent mechanism at the inlet. The cylindric titanium housing has a diameter of 16 mm and thickness of 4.5 mm. The device is 3.0 Tesla MRI safe.
**Conclusions:** The in vitro results show the interesting influence of the new tool. This might be helpful in patients with low compliance and newborns.

## A119

### Non-surgical treatment for chronic subdural hematoma on patietnts with idiopathic normal pressure hydrocephalus afetr shunt surgery

H. Murai, T. Shin, S. Ikegami, M. Kobayashi
Department of Neurosurgery, Chiba University Graduate School of Medicine, Chiba, Japan
**Correspondence:** H. Murai
*Fluids and Barriers of the CNS* 2018, **15(Suppl 1)**:A119

**Introduction:** Idiopathic normal pressure hydrocephalus (iNPH) is a disease of the elderly, who are at high risk of chronic subdural hematoma (SDH). Shunt surgery is one of the risk factors of chronic SDH. We should make every effort to minimize this complication of shunt surgery.
**Methods:** From July 2002 to May 2015, 171 cases of iNPH were diagnosed and received shunt surgery. Incidence of chronic SDH with or without hematoma surgery was examined and compared with national database. CT scans were performed at 1 week, 2 months and every 6 months after the surgery in most cases. Asymptomatic chronic SDH was treated with non-surgical treatments including pressure setting change, cessation of the antithrombotic agents, intermittent use prednisolone, tranexamic acid and/or GOREISAN.
**Results:** A median observation period was 3 years and 10 months. Chronic SDH was observed in 27 patients (15.8, 3.7% per year), with non-surgical treatments the surgery was needed in only 8 patients (4.7, 1.1% per year). Six patients were symptomatic and operated on immediately. In asymptomatic 21 cases, two cases became symptomatic and operated on later. Incidence of SDH surgery in our study seemed to be lower than that of previous reports (Birkeland et al. 2015). However, it was still 14 times higher than nationwide prevalence of chronic SDH in Japanese elderly (Toi et al. 2017).
**Conclusions:** Postoperative CT follow—up was useful for finding asymptomatic chronic subdural hematoma. Incidence of post-shunting chronic subdural hematoma surgeries could be decreased to approximately 1% per year with non-surgical treatments.

## A120

### A CSF biomarker for diagnosing spotaneous intracranial hypotension

Y. Murakami[1], K. Takahashi[2], K. Hoshi[3], H. Ito[3], M. Kanno[3], K. Saito[1], K. Nollet[4], Y. Yamaguchi[5], T. Mima[2], Y. Hashimoto[3]
[1]Departments of Neurosurgery, Fukushima Medical University, Fukushima, Japan; [2]Department of Neurosurgery, Sanno Hospital, Minato-ku, Tokyo, Japan; [3]Departments of Biochemistry, Fukushima Medical University, Fukushima, Japan; [4]Departments of Blood Transfusion and Transplantation Immunology, Fukushima Medical University, Fukushima, Japan; [5]Structural Glycobiology Team, Systems Glycobiology Research Group, RIKEN-Max Planck Joint Research Center, RIKEN Global Research Cluster, Wako, Saitama, Japan
**Correspondence:** Y. Murakami
*Fluids and Barriers of the CNS* 2018, **15(Suppl 1)**:A120

**Introduction:** Spontaneous intracranial hypotension (SIH) is caused by cerebrospinal fluid (CSF) leakage. Imaging studies are useful for identifying CSF leakage, but typical findings of intracranial hypotension are not always observed in patients with SIH. We searched for CSF biomarkers to aid the diagnosis of SIH.
**Methods:** SIH was diagnosed based on the following criteria: (i) patients have signs of CSF leakage by MRI and/or low CSF opening pressure; (ii) no recent history of dural puncture. CSF samples were obtained by lumbar puncture at Sanno Hospital from years 2013 through 2015. CSF of 38 SIH patients and 24 non-SIH patients were subjected to biochemical analysis. This study included a control 10

patients that CSF sampling from the cerebral cistern by microvascular decompression or unruptured aneurysm clipping surgery. A marker for CSF production, "brain-type" transferrin (Tf-1), was analyzed by Western blotting.

**Results:** "Brain-type" transferrin (Tf-1) was higher in CSF of SIH patients than those of non-SIH or control patients. In contrast, no significant difference was detected in the concentrations of Tf-1 between the non-SIH and control groups. Tf-1 concentration of of SIH was correlated with both RI residual activity and CSF pressure. The sensitivity, specificity, positive predictable value, and negative predictable value of Tf-1were 81.5, 79.2, 86.1, and 73.1%.

**Conclusions:** Tf-1 in CSF could be a new biomarker for diagnosing SIH.

## A121

### Threshold for the resistance to CSF outflow: should it be age-adjusted?

E. Nabbanja[1], Z. H. Czosnyka[1], A. D. Lalou[1], J. D. Pickard[1], M. Garnett[1], N. C. Keong[2], M. Czosnyka[1]
[1]Academic Neurosurgery, Cambridge University Hospital, Cambridge, UK; [2]Department of Neurosurgery, National Neuroscience Institute and Duke-NUS Medical School, Singapore, Singapore
**Correspondence:** E. Nabbanja
*Fluids and Barriers of the CNS* 2018, **15(Suppl 1)**:A121

**Introduction:** There are controversies about threshold for normal and elevated resistance to CSF outflow (Rout). Initial studies (Borgensen & Gjerris, Brain 1981) showed 100% negative and positive predictive power for improvement after shunting with the threshold of 12 mm Hg/(ml/min). 'Dutch NPH trial' (Boon et al. JNS 1997) indicated critical value of 17 mm Hg/(ml/min) with positive predictive power > 90% but negative only 34%. New European Trial (Wikkelso et al. JNNP 2012) showed no association of Rout after shunting at all.

**Methods:** We retrospectively analysed two cohorts of patients diagnosed for hydrocephalus, using constant rate computerized infusion test. First: 529 patients with all types of hydrocephalus with normal pressure (ICP < 18 mmHg), 1–90 years old, to establish relationship between compensatory parameter and age. Second: 309 patients with NPH (presented in Calgary in 2015), who have undergone infusion test, were shunted and were available for follow up in Hydrocephalus Clinic.

**Results:** Overall, Rout was significantly linearly increasing with age ($R = 0.21$; $p < 0.0001$; $N = 529$). Regression coefficient was 0.08, suggesting threshold for Rout increasing by 1 mmHg/(ml/min) per every 12.5 years increase. The best statistical separation between patients with positive reaction to shunting and patients with no improvement was detected at Rout = 9 + 0.08*age threshold. In comparison to fixed threshold of 13 mmHg/(ml/min) p value with age-corrected threshold improved from $p = 0.0156$ to $p = 0.0033$ ($N = 309$).

**Conclusions:** Our results suggest that Rout should be age-adjusted. Threshold for children should be around 9–10 mmHg/(ml/min). Threshold for 80 years old should be 15–16 mmHg/(ml/min).

## A122

### Unblocking ventricular shunt catheter during infusion test: a case study of two patients

E. Nabbanja, M. Czosnyka, J. Donnelly, J. D. Pickard, A. D. Lalou, M. Garnett, Z. H. Czosnyka, P. J. Hutchinson
Division of Neurosurgery, Department of Clinical Neurosciences, University of Cambridge, University Hospital, Cambridge, UK
**Correspondence:** E. Nabbanja
*Fluids and Barriers of the CNS* 2018, **15(Suppl 1)**:A122

**Introduction:** Infusion tests are well known for studying the dynamic properties of the CSF system, predicting outcome post shunting and testing shunt function in vivo in hydrocephalic patients. However, the proficiency of CSF Infusion Studies to unblock ventricular shunt catheters has never been performed. This paper suggests the role an infusion study can play in unblocking a ventricular shunt catheter using two case studies.

**Methods:** Two patients were identified from the Cambridge Research Database. Each patient presented with symptoms of shunt blockage and underwent assessment, Computed Tomography (CT) and constant-rate infusion test via shunt reservoir. The patients were followed up 3 months later in clinic.

**Results:** Baseline Opening pressure (OP) was within normal range (8 and 5 mmHg) for both patients. Pulse waveform was initially not-detectable in one case and reduced in the second case. After infusion was started, Intracranial Pressure (ICP) stabilised to 10 mmHg. The results suggest partial obstruction of the ventricular catheter blockage which raises the possibility of in- growing choroid plexus being the main cause. At the end of the infusion test, the ventricular catheters were flushed, using compression of siphon-control device, a manoeuvre which blocks distal flow. Following the infusion studies, both patients reported noticeable symptom improvement.

**Conclusions:** This case study echoes the reputation of testing shunt function in vivo in hydrocephalic patients. In addition, infusion study tests, on occasion, can be used as a conservative measure to unblock ventricular shunt catheters if there is a suggestion of in-growing choroid plexus.

## A123

### Shunt intervention for possible idiopathic normal pressure hydrocephalus improves modified the rankin scale by outperforming the risk of aging

M. Nakajima[1], M. Miyajima[1], I. Ogino[1], M. Kurosawa[2], N. Kuriyama[3], W. Fukushima[4], E. Mori[5], T. Kato[6], C. Akiba[1], H. Sugano[1], K. Karagiozov[1], H. Arai[1]
[1]Department of Neurosurgery, Juntendo University, Tokyo, Japan; [2]Department of Hygieiology, Juntendo University, Tokyo, Japan; [3]Department of Epidemiology for Community Health and Medicine, Graduate School of Medical Science, Kyoto Prefectural University of Medicine, Kyoto, Japan; [4]Center for Perinatal Medicine, Osaka City General Hospital, Osaka, Japan; [5]Department of Psychiatry, Osaka University Graduate School of Medicine, Japan; [6]Department of Neurology, Hematology, Metabolism, Endocrinology and Diabetology, Faculty of Medicine, Yamagata University, Yamagata, Japan
**Correspondence:** M. Nakajima
*Fluids and Barriers of the CNS* 2018, **15(Suppl 1)**:A123

**Introduction:** We provide an accurate picture of how much shunt intervention contributes to the prognoses of the patients registered in nationwide epidemiological surveys who are treated for idiopathic normal pressure hydrocephalus (iNPH).

**Methods:** We examined 1423 possible iNPH patients (581 women; mean age: 76.5 ± 6.4 years) aged 60 or older who had been diagnosed based on the clinical guidelines for iNPH following a hospital visit in 2012. Patients in whom improvement of at least one grade in the modified Rankin Scale (mRS) was achieved after intervention were considered "improved" while the remaining patients were considered "non-improved." A shunt intervention group ($n = 974$) and non-intervention group ($n = 449$) were statistically analyzed by binomial logistic regression analysis.

**Results:** Confounding factors in shunt intervention for possible iNPH are age (odds ratio [OR]: 0.96, 95% CI: 0.94–0.98), gait disturbance (1.76; 1.28–2.42), and Alzheimer's disease comorbidity (0.63; 0.44–0.90). After adjusting for these confounding factors, the improvement in the intervention group was 8.81-fold (6.24–12.44). A significant improvement in mRS grade was achieved in the intervention group (54% improvement compared with 7% improvement in the non-intervention group), even among 509 patients aged 80 or older. Risk factors for improvement in mRS grade following shunt intervention were a low preoperative mRS (OR: 0.47; 0.38–0.59), a negative result in the tap test (1.93; 1.11–3.36), Alzheimer's disease comorbidity (1.87; 1.17–2.99), chronic ischemic lesions (1.65; 1.01–2.68), and age (1.03; 1.01–1.06).

**Conclusions:** Shunt intervention's outperformance of the risk of old age could serve as the basis for considering shunt intervention even in elderly patients if indications are taken into account.

### A124

**Neuropsychiatric symptoms in idiopathic normal pressure hydrocephalus: presurgical feature and the 1-year changes after the shunt surgery**

W. Narita, Y. Nishio, T. Baba, O. Iizuka, Y. Shoji, K. Kakinuma, E. Mor
Department of Behavioral Neurology and Cognitive Neuroscience, Tohoku University Graduate School of Medicine, Sendai, Japan
**Correspondence:** W. Narita
*Fluids and Barriers of the CNS* 2018, **15(Suppl 1)**:A124

**Introduction:** The classical triad of normal pressure hydrocephalus were gait, cognitive, and urinary disturbance. Neuropsychiatric symptoms were also common clinical features. However, there were few previous studies which investigated these symptoms and the changes of these symptoms after surgery. The purpose of the current study was to demonstrate neuropsychiatric symptoms and the response to shunt surgery in idiopathic normal pressure hydrocephalus (iNPH).

**Methods:** Seventy-one patients with idiopathic normal pressure hydrocephalus underwent presurgical clinical evaluation including neuropsychiatric symptoms before and 1 year after shunt surgery. We used the idiopathic normal pressure hydrocephalus grading scale (iNPHGS) for individual symptom such as gait, cognitive, and urinary disturbance, and evaluated neuropsychiatric symptoms by the neuropsychiatric inventory.

**Results:** Mean age of the patients was 76.3 (SD 3.9), and 40 patients (56%) were male. Mean duration of symptoms was 2.8 (SD 1.7) years. The mean mRS was $2.7 \pm 1.0$ and the mean iNPHGS total, subdomain scores for gait, cognitive, urinary disturbance were $6.6 \pm 1.9$, $2.3 \pm 0.7$, $2.4 \pm 0.8$, $1.8 \pm 1.1$, respectively. 23 (32%) patients underwent lumboperitoneal shunt surgery. 64 (90.1%) of the patients had at least one of the neuropsychiatric symptoms before the shunt surgery. Apathy (78.9%), cognitive fluctuation (40.8%), and irritability (36.8%) were common symptoms. Any of the symptoms were seen in 56 (73.1%) of the patients after the shunt surgery. Prevalence of apathy, cognitive fluctuation, and irritability were decreased to 59.2, 14.1, and 26.8%, respectively.

**Conclusions:** As well as classical triad, neuropsychiatric symptoms were common and shunt-responsive symptoms in iNPH.

### A125

**Comparison of microfiber alteration of corticospinal tract in normal pressure hydrocephalus patients and normal controlled subjects by diffusion tensor imaging**

C. Ngamsombat[1], K. Lerdthusnee[1], Z. Zhang[2], H. Guo[2], T. Witthiwej[3], W. Muangpaisan[4], J. Wongsripuemtet[1], Y. Vichianin[5], P. Charnchaowanish[1], O. Chawalparit[1]
[1]Department of Radiology, Faculty of Medicine Siriraj Hospital, Mahidol University, Bangkok, Thailand; [2]Center for Biomedical Imaging Research, Department of Biomedical Engineering, School of Medicine, Tsinghua University, Beijing, China; [3]Division of Neurosurgery, Department of Surgery, Faculty of Medicine Siriraj Hospital, Mahidol University, Bangkok, Thailand; [4]Department of Preventive and Social Medicine, Faculty of Medicine Siriraj Hospital, Mahidol University, Bangkok, Thailand; [5]Department of Radiological Technology, Faculty of Medical Technology, Mahidol University, Bangkok, Thailand
**Correspondence:** C. Ngamsombat
*Fluids and Barriers of the CNS* 2018, **15(Suppl 1)**:A125

**Introduction:** Idiopathic normal pressure hydrocephalus (iNPH) is one of the treatable causes of dementia in elderly by shunt placement surgery. Diffusion tensor imaging (DTI) can demonstrate the alteration of white matter microfiber. The corticospinal tract (CST) is white matter fiber responsible in part of motor function and gait. This study was to compare microfiber alteration of the CST between iNPH patients and normal controlled subjects.

**Methods:** Fifteen clinically diagnosed definite iNPH patients and 15 normal controlled subjects underwent MRI study with DTI. All iNPH patients had gait disturbance and clinical improvement after shunt placement surgery. Probabilistic tractography of the CST were performed and comparison of DTI parameters included fractional anisotropy (FA), mean diffusivity (MD), axial diffusivity (AD) and radial diffusivity (RD) in iNPH and normal controlled groups.

**Results:** There were statistically significant increase of MD ($p < 0.001$), AD ($p < 0.001$) and RD ($p < 0.001$) in iNPH patients compared with normal controlled subjects, whereas FA of iNPH patients was slightly decreased but not statistically significant different when compared with the normal controlled group ($p = 0.75$).

**Conclusions:** DTI parameters, especially MD, AD and RD can be used to evaluate alteration of white matter microfiber of the CST, which was responsible in part of alteration of motor function and gait disturbance in iNPH patients. We hope that our study will lead to further investigation about other role of DTI in iNPH patients.

### A126

**Effect of pre-installed shunt on endoscopic cerebrospinal fluid diversion procedure for hydrocephalus**

N. Nishida[1], H. Hashikata[1], N. Ikeda[2], Y. Kang[3], Y. Miki[1], J. Taki[1], M. Gotoh[1], N. Yoshimoto[1], H. Toda[4], M. Ishikawa[5], K. Iwasaki[1]
[1]Department of Neurosurgery, Tazuke Kofukai Kitano Hospital, Osaka, Japan; [2]Department of Neurosurgery, Osaka Medical College Hospital, Osaka, Japan; [3]Department of Neurosurgery, Ohno Memorial Hospital, Osaka, Japan; [4]Department of Neurosurgery, Fukui Red Cross Hospital, Fukui, Japan; [5]Normal Pressure Hydrocephalus Center, Rakuwakai Otowa Hospital, Kyoto, Japan
**Correspondence:** N. Nishida
*Fluids and Barriers of the CNS* 2018, **15(Suppl 1)**:A126

**Introduction:** Neuroendoscopic cerebrospinal fluid (CSF) diversion procedure is considered as a choice of treatment for shunt malfunction. We compared the clinical course and complications of the procedure between patients with newly diagnosed hydrocephalus and those with previous shunts.

**Methods:** We retrospectively reviewed 37 patients ($50.6 \pm 19.6$ years old, M23/F14) undergoing neuroendoscopic procedure from 2006 to 2016 in Kitano hospital. There were 16 cases of mass lesions causing non-communicating hydrocephalus, 15 cases of idiopathic aqueductal stenosis, 3 cases of fourth ventricle outlet obstruction and 3 cases of isolated fourth ventricle. Effect of pre-installed shunts on the number of subsequent procedures and complications was analyzed with Fisher's exact test.

**Results:** Previous shunts were found in 5 patients (3 cases of isolated fourth ventricle, 1 case of fourth ventricle outlet obstruction and 1 case of aqueductal stenosis). Two of them needed another surgical procedures (shunt ligation with third ventriculostomy for one, and cranio-cervical arachnoid dissection for syringomyelia for another case). One of them experienced meningitis. Among patients with newly diagnosed hydrocephalus, 3 cases needed additional shunt procedure and 1 case experienced chronic subdural hematoma cured by another burr hole surgery. Relative risk ratio of additional CSF diversion procedure was 4.27 (95% C.I. 0.93–19.5) and that of complications was 6.4 (95% C.I. 0.47–86.8) for patients with pre-installed shunts, which did not reach statistical significance.

**Conclusions:** CSF dynamics of hydrocephalus already treated by shunt systems are sometimes very complicated. Neuroendoscopic CSF diversion is effective treatment for selected cases, though we have to recognize its potential pitfalls.

### A127

**Role of the brain and cerebrospinal fluid factors in the pathophysiology of secondary normal pressure hydrocephalus**

S. Nomura, S. Shirao, H. Ishihara, E. Suehiro, N. Tanaka, F. Oka, M. Suzuki
Department of Neurosurgery, Yamaguchi University Graduate School of Medicine, Ube, Japan
**Correspondence:** S. Nomura
*Fluids and Barriers of the CNS* 2018, **15(Suppl 1)**:A127

**Introduction:** To evaluate the contribution weights of various risk factors to the prevalence of secondary normal pressure hydrocephalus (sNPH) following subarachnoid hemorrhage (SAH) due to ruptured aneurysm. The risks were classified as either brain factors (i.e. insult to brain tissue) or cerebrospinal fluid (CSF) factors (i.e. factors affecting the CSF), in order to discuss the associated pathophysiology.

**Methods:** From 1998 to 2015, 256 patients with Fisher group 3 SAH were treated at the Yamaguchi University Hospital. Five variables, i.e. age, initial Hunt and Kosnik (H&K) grade, location of the aneurysm, surgical method used to treat the aneurysm, and the drainage method were analyzed.

**Results:** A total of 53 (20.7%) patients developed sNPH. Patients with sNPH were significantly older compared to those without sNPH (65.5 ± 10.8 years vs. 61.0 ± 13.1 years, p = 0.03). Significantly more patients who developed sNPH underwent clipping and cisternal drainage, compared with coiling (23.6% vs. 8.3%, p = 0.03) and lumbar drainage (23.7% vs. 11.3%, p = 0.04), respectively. The H&K of patients with sNPH was significantly poor (p < 0.001), compared to that of those without sNPH. According to the standardized partial regression coefficients, the order of contribution of the factors was as follows: H&K (0.27), being older (0.22), clipping (0.22), and cisternal drainage (0.04).

**Conclusions:** Poor neurological grade, being older, and clipping with craniotomy have a greater risk, since they can alter the compliance of brain tissue. Adequate management to protect vulnerable brains of the elderly individuals and patients with poorer neurological grades are important to prevent the development of sNPH. Finally, although lumbar drainage effectively eliminated blood from CSF, it had little influence on preventing sNPH.

## A128

**Withdrawn**

*Fluids and Barriers of the CNS* 2018, **15(Suppl 1)**:A128

## A129

**Apparent hyperperfusion on convexity is useful for the diagnosis of idiopathic normal pressure hydrocephalus**

T. Ohmichi[1], M. Kondo[1], M. Itsukage[1], H. Koizumi[1], S. Matsushima[2], N. Kuriyama[3], K. Ishii[4], E. Mori[5], K. Yamada[2], T. Mizuno[1], T. Tokuda[1,6]

[1]Department of Neurology, Kyoto Prefectural University of Medicine, Kyoto, Japan; [2]Department of Radiology, Kyoto Prefectural University of Medicine, Kyoto, Japan; [3]Department of Epidemiology for Community Health and Medicine, Kyoto Prefectural University of Medicine, Kyoto, Japan; [4]Department of Radiology, Kindai University Faculty of Medicine, Osaka, Japan; [5]Department of Behavioral Neurology and Cognitive Neuroscience, Tohoku University Graduate School of Medicine, Sendai, Japan; [6]Department of Molecular Pathobiology of Brain Diseases, Kyoto Prefectural University of Medicine, Kyoto, Japan

**Correspondence:** T. Ohmichi

*Fluids and Barriers of the CNS* 2018, **15(Suppl 1)**:A129

**Introduction:** On magnetic resonance imaging (MRI), high-convexity tightness is an important finding for the diagnosis of idiopathic normal pressure hydrocephalus (iNPH). On single photon emission computed tomography (SPECT), iNPH patients often show hyperperfusion of the high-convexity area. To test two hypotheses regarding the SPECT finding: (1) it is a relative hyper-perfusion reflecting the increased gray matter density of the convexity, and (2) it is useful for the diagnosis of iNPH. We termed it the Convexity APPArent Hyperperfusion (CAPPAH) sign.

**Methods:** We conducted two clinical studies. In Study 1, we performed SPECT in 20 patients suspected of having iNPH, examined the regional cerebral blood flow (rCBF) of the high-convexity area, and compared clinical differences between patients with the CAPPAH sign (CAP) and those without it (NCAP). In Study 2, we assessed the CAPPAH sign in 30 iNPH patients and 19 healthy controls using SPECT.

**Results:** In Study 1, the rCBF of the high-convexity area of CAP was calculated as 35.2–43.7 mL/min/100 g, which is not higher than the normal values of rCBF determined by SPECT. The NCAP group showed a lower cognitive function and weaker responses to the removal of cerebrospinal fluid than the CAP group. In Study 2, the CAPPAH sign was positive only in iNPH patients (24/30), and not in controls (sensitivity 80%, specificity 100%).

**Conclusions:** INPH patients showed hyper-perfusion of the high-convexity area on SPECT; however, the presence of the CAPPAH sign did not indicate real hyperperfusion of rCBF in the high-convexity area.

## A130

**Usefulness of videotape-record for detection of patients with idiopathic normal pressure hydrocephalus**

T. Okada

Department of Neurosurgery, Tokyo Metropolitan Tama-Hokubu Medical Center, Tokyo, Japan

**Correspondence:** T. Okada

*Fluids and Barriers of the CNS* 2018, **15(Suppl 1)**:A130

**Introduction:** The triad of idiopathic normal pressure hydrocephalus (iNPH), gait disturbance, incontinence and dementia, is so common among elderly people that detection of patients is complicated. Comprehension of clinical course by caregivers may be useful, because symptoms of iNPH change with time. Moreover, noticing iNPH by patients themselves may contribute to early detection.

**Methods:** Gait disturbance was classified by severity into five grades, grade 0–4, using Japanese iNPH Grading Scale (iNPHGS.) Patients submitted to videotape-record (VTR) before and after surgery. VTR was assessed for not only estimation of operative result but of transition of gait disturbance. Sixty seven patients underwent shunting since 1998, and 45 were improved. Eight were selected from them and sorted by iNPHGS excluding grade 0 (normal gait.) Their preoperative VTRs were arranged for evaluating progression of gait disturbance. Four patients of grade 1, whose affliction was confined to subjective complains such as swaying or dullness in legs, were estimated to find postoperative changes.

**Results:** Having clear differences, iNPHGS was useful not only in estimating operative result but in assessing progression of gait disturbance. It began with apparently normal walk for their age then proceeded to wobbling, shuffling, wide-based walk, walking with groping for or grabbing objects, and finally to inability to stand. Patients of grade 1 walked fast and straight almost to the point of normal gait after operation.

**Conclusions:** Assessment of gait disturbance with VTR may be a useful measure in detecting iNPH for caregivers and, especially, for patients in early stage.

## A131

**Neuropsychological and affective evaluation in idiopathic normal pressure hydrocephalus patients pre and post cerebrospinal fluid tap test**

F. Oppi[1], G. Palandri[2], P. Mantovani[2], A. Valluzzi[2], S. Cevoli[1], G. Giannini[1], L. Albini Riccioli[3], D. Milletti[4], C. Sturiale[2], P. Cortelli[1]

[1]Operative Unit of Neurology, Institute of Neurological Sciences of Bologna, Bologna, Italy; [2]Operative Unit of Neurosurgery, Institute of Neurological Sciences of Bologna, Bologna, Italy; [3]Operative Unit of Neuroradiology, Institute of Neurological Sciences of Bologna, Bologna, Italy; [4]Operative Unit of Rehabilitation Medicine, Institute of Neurological Sciences of Bologna, Bologna, Italy

**Correspondence:** G. Palandri

*Fluids and Barriers of the CNS* 2018, **15(Suppl 1)**:A131

**Introduction:** iNPH affects preferentially older patients and is associated to cognitive deficits. Shunt surgery showed mixed results on cognitive deficits and very little is known on results after a cerebrospinal fluid (CSF) tap test (TT).

**Methods:** we prospectively evaluated the effect of TT on cognitive function and affective status in a sample of iNPH subjects. Since November 2015 to May 2017 we recruited a continuative sample of 55 (age: 73 ± 5; Education: 10 ± 4; M = 33; F = 22) iNPH patients. All patients were examined by a neurologist and then underwent an extensive neuropsychological and affective evaluation pre and post (72 h) TT, using parallel forms in every session. Neuropsychological (NPS) evaluation comprises a selection of tests exploring memory, attention, executive and posterior cortical functions (i.e. verbal, visual and visuospatial memory; sustained and selective visual attention; language and lexical production; visuospatial, constructive and praxical abilities). We utilized: Mini Mental Status Examination, Brief Battery for Mental Deterioration (constituted by: Rey Auditory Verbal Learning Test, Multiple Feature Target Cancellation, Immediate Visual Memory, Simple Verbal Analogies Test, Copying Designs Simple Copy, Phonemic Word Fluency, Semantic Word Fluency, Stroop Color Word Test, Digit Span Forward, Digit Span Backward, Corsi Block-Tapping Test, Rey-Osterrieth Complex Figure Test and Frontal Assessment Battery). Affective status was explored by two self-administered questionnaires: Beck Depression Scale and State-Trait Anxiety Inventory.
**Results:** patients' scores were first corrected for age and education and then compared to normal ranges, according to published Italian norms. Our results show improvement in executive functions and visuospatial constructive abilities. There was also a significant improvement in depression. On visual side TT lead to faster and more accurate performance in sustained and selective attention tests and, on verbal side, to better logic reasoning and increased access to phonemic source. It is interesting to observe that posterior abilities seems to be positively influenced as well as anterior ones showing that improvement could include a widespread spectrum of different cerebral areas. We also noticed that affective status, albeit not varying in a relevant way, seems to be positively influenced on the depressive side and not on the anxiety one.
**Conclusions:** iNPH patients have a selective, objective and documented enhancement of executive functions after TT. In our preliminary multidisciplinary experience ("pro-hydro" group), NPS evaluation has been crucial to refer patients for surgical treatment. In the light of these results, an in-depth and standardized NPS assessment seems to be highly valuable.

## A132

### Multidisciplinary management of idiopathic normal pressure hydrocephalus: a preliminary experience

G. Palandri[1], P. Mantovani[1], A. Valluzzi[1], S. Cevoli[2], G. Giannini[2], L. Albini Riccioli[3], D. Milletti[4], F. Oppi[2], C. Sturiale[1], P. Cortelli[2]
[1]Operative Unit of Neurosurgery, Institute of Neurological Sciences of Bologna, Bologna, Italy; [2]Operative Unit of Neurology, Institute of Neurological Sciences of Bologna, Bologna, Italy; [3]Operative Unit of Neuroradiology, Institute of Neurological Sciences of Bologna, Bologna, Italy; [4]Operative Unit of Rehabilitation Medicine, Institute of Neurological Sciences of Bologna, Bologna, Italy
**Correspondence:** G. Palandri
*Fluids and Barriers of the CNS* 2018, **15(Suppl 1)**:A132

**Introduction:** We present the preliminary results of a prospective longitudinal study based on a standardized protocol for a multidisciplinary evaluation of Idiopathic Normal Pressure Hydrocephalus (iNPH).
**Methods:** since 2015 patients referred for suspected iNPH were evaluated by dedicated specialists. If clinical and radiological findings were suggestive for iNPH, cases were discussed in the "pro-hydro" team, composed by neurosurgeons, neurologists, neuroradiologists, physiatrists and neuropsychologists of the Institute of Neurological Sciences at Bellaria Hospital in Bologna (ISNB). Inclusion criteria were clinical and radiological findings compatible with iNPH in patients older than 50 years (T0). These patients underwent a 3T MRI (T1). Patients were then collegially discussed again (T2) and admitted (T3) at ISNB for standardized clinical, physical and cognitive evaluation, computerized gait assessment before and after (24 and 72 h) lumbar tap test (TT). Results were again discussed by the team to select patients for ventriculoperitoneal shunt (VPS) (T4) or for observation. Follow-up included in every case radiological and clinical evaluation at 1 (T5), 6 (T6) and 12 months (T7).
**Results:** in the period from January 2015 to January 2017, 97 consecutive patients were evaluated by the "pro-hydro" team. One patient refused, one patient had an ischemic stroke and thus dropped out (T0). 8 patients are still waiting to undergo 3T MRI (T1). 19 out of 87 patients were excluded after the MRI for various reasons (i.e. aqueductal stenosis); other 10 patients dropped out before admission. 4 of the remaining are waiting to be admitted, while 54 have already been admitted and studied according to the protocol; 31 are being managed conservatively (3 have been proposed a VPS, 10 dropped out, 10 have been evaluated at T6, 4 at T7). The other 21 patients have been operated (T4-T5), 11 have a follow-up at 6 months (T6), 4 at T7.
**Conclusions**: iNPH is a complex and often misdiagnosed syndrome. A preliminary consult with a qualified specialist, a multidisciplinary evaluation and the enrolment in a dedicated study protocol such as the "pro-hydro", help achieving a correct diagnosis and management of iNPH patients providing a standardized patient selection criteria for VPS.

## A133

### Efficacy and safety of ventricular lavage therapy for post intraventricular hemorrhagic hydrocephalus in low birth weight infants

Y-S. Park, Y. Kotani, H. Yokota, Y. Motoyama, H. Nakase
Department of Neurosurgery, Nara Medical University, Kashihara, Japan
**Correspondence:** Y-S Park
*Fluids and Barriers of the CNS* 2018, **15(Suppl 1)**:A133

**Introduction:** The management of post intraventricular hemorrhagic hydrocephalus (PIVHH) in extreme and very low birth weight infants (LBWIs) is challenging and controversial. We tried to remove out bloody cerebrospinal fluid via external ventricular drainage (EVD) combined with urokinase (UK) injection into lateral ventricle, called "Ventricular Lavage (VL) therapy", from the early stage of disease. The aim of this study was to evaluate safety and efficacy of our original therapy with presenting specific cases.
**Method:** In total thirty four consecutive LBWIs with PIVHH (IVH grade 3: 17 cases, IVH grade 4: 17 cases) were analyzed. Large part of infants was extreme LBWIs (< 1000 g birth weight at birth). We conducted early EVD management in fifteen cases and additionally performed Ventricular Lavage therapy in eleven cases. On the other hand, according to the judgment of neonatologists, treatment was delayed in eighteen cases.
**Results:** Ten of the twelve extreme LBWIs who underwent VL therapy did not require V–P shunt surgery. And there were no serious complications associated with VL therapy including secondary hemorrhage and infection. In the majority of eighteen patients treated in the late stage, permanent shunt placement was necessary, and many serious shunt related complications occurred. We defined their neurological outcomes were good, if self-walking, verbal communication and self-feeding were satisfactory at 36 month-old. Nine of eleven cases in the early treatment group and six of twelve cases in the late treatment group were good clinical results. Surprisingly, despite the majority of severe IVH grade 4, the early treatment group was significantly better.
**Conclusion:** Permanent shunt surgery was dramatically reduced compared with delayed treatment group. Continuous reducing intracranial pressure, acceleration clot dissolution and prevention of fibrin adhesion could reduce not only the shunt dependency rate but the white matter damage.

## A134

### Ventricle-sylvian fissure shunt for obstructive hydrocephalus

S. Patkar[1,2]
[1]Poona Hospital and Research Center, Pune, India; [2]Bhartividyapeeth Medical College and Hospital, Pune, India
**Correspondence:** S. Patkar
*Fluids and Barriers of the CNS* 2018, **15(Suppl 1)**:A134

**Introduction:** The final pathway for intracranial CSF is via the sylvian fissure to the cerebral convexity subarachnoid space from where it reaches the superior saggital sinus to enter the blood stream. Diverting CSF from the ventricle to the sylvian fissure in obstructive hydrocephalus should result in relief of hydrocephalus.
**Methods:** 5 patients with obstructive hydrocephalus, with inability to undergo ventriculoperitoneal or ventriculoatrial shunt due to previous medical or technical reasons underwent the procedure.
**Results:** All patients had immediate relief of raised pressure. Follow up up to 12 months in three patients show that the ventricle size remains diminished.
**Conclusions:** Diverting CSF from the lateral ventricle to the sylvian fissure using an inexpensive shunt catheter seems to be easy as microsurgical dissection of sylvian fissure is standard part of neurosurgical training22. As both ends of the shunt are intracranial, over drainage seems to be an unlikely complication. The surgical field is small and duration of procedure being short it would translate into lesser chances of infection. All the complications of the peritoneal catheter of a ventricle-peritoneal shunt seem preventable.

## A135
### Withdrawn
*Fluids and Barriers of the CNS* 2018, **15(Suppl 1)**:A135

## A136
### Benefit of additional SF-36 quality of life and truncal balance tests for holistic evaluation in shunted INPH patients; a prospective study
K. Petchsook, E. Chankaew, P. Srirabheebhat
Division of Neurosurgery, Department of Surgery, Faculty of Medicine Siriraj Hospital, Mahidol University, Bangkok, Thailand
**Correspondence:** K. Petchsook
*Fluids and Barriers of the CNS* 2018, **15(Suppl 1)**:A136

**Introduction:** The classical triads of idiopathic normal pressure hydrocephalus (iNPH) are gait difficulty, memory decline, and urinary incontinence. From our clinical practice, the patients might have varied clinical spectrums including balance, speech, and swallowing problem. Widely used Japanese grading scale for NPH (JSNPH) represents only classical triads. Some amount of shunted patients who were classified as non-improvement by JSNPH might improve in other aspects. This study is to investigate health related quality of life (HRQOL) and truncal balance in shunted iNPH patients as additional tools for evaluation of the shunt responsiveness.
**Methods:** This prospective study was performed to compare pre- and post-operative clinical outcomes in iNPH patients treated at Siriraj Hospital between 2014 and 2016. The pre-operative, and 1-, 3-, and 6-month post-operative clinical outcomes using JSNPH, mRS, SF-36 health survey instrument, and truncal balance test were observed.
**Results:** Total of 32 iNPH patients was included in our study. All of the patients had lower level of HRQOL than an age-adjusted reference population. Statistically significant clinical outcome improvement was achieved in 81.25% of JSNPH, 68.75% of mRS, and 87.5% of SF-36 health survey scores. All domains of SF-36 improved after shunt implantation. Also patients who suffered from truncal imbalance were demonstrated significant improvement of their sitting and standing balance scores after shunt surgery.
**Conclusions:** Commonly used clinical scales have some limitations in evaluation of shunt responsiveness in iNPH patients. We propose the addition of HRQOL and truncal balance test to increase the effectiveness of the evaluation tools.

## A137
### Benefit of additional SF-36 quality of life and truncal balance tests for holistic evaluation in shunted INPH patients; a prospective study
K. Petchsook, E. Chankaew, P. Srirabheebhat
Division of Neurosurgery, Department of Surgery, Faculty of Medicine Siriraj Hospital, Mahidol University, Bangkok, Thailand
**Correspondence:** K. Petchsook
*Fluids and Barriers of the CNS* 2018, **15(Suppl 1)**:A137

**Introduction:** The classical triads of idiopathic normal pressure hydrocephalus (iNPH) are gait difficulty, memory decline, and urinary incontinence. From our clinical practice, the patients might have varied clinical spectrums including balance, speech, and swallowing problem. Widely used Japanese grading scale for NPH (JSNPH) represents only classical triads. Some amount of shunted patients who were classified as non-improvement by JSNPH might improve in other aspects. This study is to investigate health related quality of life (HRQOL) and truncal balance in shunted iNPH patients as additional tools for evaluation of the shunt responsiveness.
**Methods:** This prospective study was performed to compare pre- and post-operative clinical outcomes in iNPH patients treated at Siriraj Hospital between 2014 and 2016. The pre-operative, and 1-, 3-, and 6-month post-operative clinical outcomes using JSNPH, mRS, SF-36 health survey instrument, and truncal balance test were observed.
**Results:** Total of 32 iNPH patients was included in our study. All of the patients had lower level of HRQOL than an age-adjusted reference population. Statistically significant clinical outcome improvement was achieved in 81.25% of JSNPH, 68.75% of mRS, and 87.5% of SF-36 health survey scores. All domains of SF-36 improved after shunt implantation. Also patients who suffered from truncal imbalance were demonstrated significant improvement of their sitting and standing balance scores after shunt surgery.
**Conclusions:** Commonly used clinical scales have some limitations in evaluation of shunt responsiveness in iNPH patients. We propose the addition of HRQOL and truncal balance test to increase the effectiveness of the evaluation tools.

## A138
### Cryptococcal meningitis causing obstructive hydrocephalus in a patient on fingolimod
C. Pham, I. Bennett, R. Jithoo
Department of Neurosurgery, Alfred Health, Melbourne, Australia
**Correspondence:** C. Pham
*Fluids and Barriers of the CNS* 2018, **15(Suppl 1)**:A138

**Introduction:** The long-term adverse effect profile of fingolimod, an immunomodulating agent approved for use in multiple sclerosis in 2010, is only just emerging. Our aim is to present obstructive hydrocephalus secondary to acute cryptococcosis as a rare complication of fingolimod use and discuss timing of ventricular shunting in cryptococcal infection.
**Methods:** We report the case of a sixty-one-year-old lady presenting with 2-weeks of headache, neck pain, nausea and vertigo. Her medical history was significant for relapsing–remitting multiple sclerosis diagnosed 10 years prior and she was commenced on fingolimod (Gilenya™) 3 years prior.
**Results:** Full blood examination showed profound lymphopaenia (120/µl) with a CD4 count of 5/µl, CD8 count of 9/µl. Serum and cerebrospinal fluid cryptococcal antigen serology were positive. Magnetic resonance imaging showed extensive posterior fossa leptomeningeal nodular enhancement and oedema causing mass effect on the fourth ventricle, obstructive hydrocephalus and early cerebellar tonsillar herniation. An external ventricular drain was inserted on an urgent basis before being converted to a ventriculoperitoneal shunt 7 days later. Antifungal therapy with liposomal amphotericin B and flucytosine was commenced.
**Conclusions:** This is the first documented case of fingolimod immunosuppression resulting in severe central nervous system cryptococcosis and hydrocephalus requiring urgent decompression. The adverse effects of fingolimod as a disease modifying agent in multiple sclerosis is not well understood and further cases of opportunistic infections are likely to emerge as its utility increases. Symptoms of hydrocephalus and meningoencephalitis may overlap and physician vigilance is required for prompt investigation and management.

## A139
### Long term follow-up of antibiotic-impregnated hydrocephalus shunt catheters: evidence from the UK shunt registry
J. D. Pickard, H. K. Richards, H. M. Seeley, A. Joannides
Academic Neurosurgery Unit & NIHR Brain Injury HTC, University of Cambridge, Addenbrookes Hospital, Cambridge, UK
**Correspondence:** J. D. Pickard
*Fluids and Barriers of the CNS* 2018, **15(Suppl 1)**:A139

**Introduction:** In addition to evolution of institutional protocols including surgical technique and antibiotic prophylaxis for hydrocephalus shunt operations, antibiotic-impregnated catheters (AICs) have been shown to reduce the incidence of revision operations for infection. However, there has been concern based on anecdotal reports that AICs may lead to late infections caused by antibiotic-resistant organisms. Is there an increased incidence of late infections with AICs than with conventional catheters?
**Methods:** The United Kingdom Shunt Registry is a paper-based reporting system for CSF shunts inserted in the 34 UK neurosurgery units since May 1995. This Report is based on data downloaded on 23rd January 2015 from the master database that gave a shunt procedure dataset from 1st January 1995 to 31st December 2014 of 53,767 procedures in 29,341 patients. The infection risk was calculated as the proportion of procedures subsequently revised for infection based on "intention to treat" recorded at the time of surgery where the follow-up was greater than 9 months. Subsequent bacteriological confirmation was not available.

**Results:** Two cohorts matched for age, diagnosis, number of previous procedures and gender have been examined—for operations prior to the end of 2006 (994 matched pairs) and 2007–2014 (4011 matched pairs). There was an overall reduction in shunt infection rate using conventional catheters from 4.7 to 1.87% between the two cohorts. Bactiseal catheters significantly reduced the infection risk in both cohorts (to 3% $p = 0.048$ and 1.12% $p = 0.006$ respectively). The first cohort has been followed up long term. Importantly, there were late infections in both the conventional (from 4.7 to 5.1%) and the Bactiseal groups (from 3 to 3.3%). The beneficial effect of Bactiseal was maintained long term.

**Conclusions:** There is a similar low incidence of late infections with both conventional and antibiotic-impregnated hydrocephalus shunt catheters. The bacteriological causes of such late infections will require a prospective multicentre study.

## A140

### The Timed Up and Go (TUG) cut off for normal pressure hydrocephalus

G. Mendes[1,3], M. Oliveira[2,3], M. Teixeira[3], F. Pinto[3]

[1]Department of Physiotherapy, Hospital do Servidor Público Estadual de São Paulo, São Paulo, Brazil; [2] Department of Neurosurgery, Hospital do Servidor Público Estadual de São Paulo, São Paulo, Brazil; [3] Group of Cerebral Hydrodynamics, Division of Functional Neurosurgery, Institute of Psychiatry, Hospital das Clínicas, University of São Paulo, São Paulo, Brazil

**Correspondence:** F. Pinto

*Fluids and Barriers of the CNS* 2018, **15(Suppl 1)**:A140

**Introduction:** The Timed Up and Go (TUG) has been regarded as an important test for Normal Pressure Hydrocephalus (NPH) evaluation. However, its sensitivity and specificity for NPH during diagnosis and prognosis process is unknown. Objective: To define a cut off point for TUG for individuals diagnosed with NPH who have independent gait (grade 1 in Japanese grading scale for NPH–JGS).

**Methods**: A group of 26 patients with NPH was submitted to CT or MRI for Evan´s index, Mini Mental State Test (MM), TUG and JGS, before doing the Tap Test, 3 and 72 h after that. After VP shunt, patients were evaluated 3, 6 and 12 months. A control group composed of 30 healthy individuals of similar age and no NPH diagnosis were submitted to the TUG to determine an average time which was then compared to patients with NPH. The Shapiro–Wilk test was used to evaluate normality in the variables of interest, a paired *t*-test was used to assess the MM, JGS, and Evan's index variables. The Wilcoxon test was used to assess the TUG variable and the ROC curve analysis was used to determine TUG cut off point.

**Results:** TUG showed specificity of 0.967 and sensitivity of 0.933 for cut off value of 16.5 s for the prognosis of NPH.

**Conclusions:** The TUG shows the best specificity and sensitivity for patients who have NPH and independent gait, with a mean value of 16.5 s.

## A141

### Nordic multicenter study on the use of the pulsatility curve to guide shunt opening pressure

S. Qvarlander[1], A. Eklund[1], D. Farahmand[2], T. Huttunen[3], P. Jokinen[4], L-O. Koskinen[5], A. Kotkansalo[4], B. Kristensen[6], V. Leinonen[3], L. Stridh[7], M. Tullberg[2], J. Virhammar[7], J. Malm[5]

[1]Department of Radiation Sciences, Umeå University, Umeå, Sweden; [2]Department of Clinical Neuroscience, Sahlgrenska University Hospital, Gothenburg, Sweden; [3]Department of Neurosurgery, Kuopio University Hospital, Kuopio, Finland; [4]Department of Neurosurgery, Turku University Hospital, Turku, Finland; [5]Department of Pharmacology and Clinical Neuroscience, Umeå University, Umeå, Sweden; [6]Department of Clinical Medicine, Aalborg University Hospital, Aalborg, Denmark; [7]Department of Neuroscience, The Uppsala University Hospital, Uppsala, Sweden

**Correspondence:** S. Qvarlander

*Fluids and Barriers of the CNS* 2018, **15(Suppl 1)**:A141

**Introduction:** There is no well-developed strategy for how to utilize the adjustable opening pressure of modern shunts to optimize individual treatment effect. In idiopathic normal pressure hydrocephalus (INPH) there is also a need for better ways to identify patients that will improve after shunt surgery. The pulsatility curve describes the relationship between intracranial pressure (ICP) and cardiac-related ICP pulse amplitude, and indicates how pulse amplitude can be reduced, and compliance increased, by decreasing ICP, e.g. via shunt surgery. The objective of this study is to investigate if the pulsatility curve can be used: (1) as a predictive test in INPH and (2) to determine the optimal individual opening pressure.

**Methods:** Six centers will collect data from INPH subjects before and after shunt surgery in a double-blind randomized multicenter study. Data collection involves infusion investigations using CELDA®, including analysis of the pulsatility curve, and outcome measures describing gait/balance, cognition, continence and quality of life. At follow-up 3 months after surgery, outcome prediction will be evaluated, whereafter subjects are randomized into two groups: (1) shunt adjustment according to the pulsatility curve and (2) fixed opening pressure. The groups will be compared according to outcome measures at 6 months.

**Results:** The study has ethical approval. Based on a power analysis 150 subjects will be included. Data collection is planned for Spring 2017–2018.

**Conclusions:** We hope to demonstrate that the pulsatility curve can be used to optimize the shunt opening pressure for individual subjects, and that it is an efficient predictive test in INPH.

## A142

### The estimated burden of disease for normal pressure hydrocephalus

C. Garcia-Baena, J. S. Davidson, M. F. Cárdenas, J. Mendoza, F. Hakim, J. F. Ramón

Neurosurgery Section, Department of Surgery, Fundación Santa Fe de Bogotá, Bogotá, Colombia

**Correspondence:** C. Garcia-Baena

*Fluids and Barriers of the CNS* 2018, **15(Suppl 1)**:A142

**Introduction:** Normal Pressure Hydrocephalus is now recognised as a cause of dementia, presenting classically as the Hakim triad of gait disturbance, urinary incontinence and dementia. Most of its medical importance lies on its potential reversibility with surgical management. Prevalence estimates are not negligible, though variable, ranging from 3.3 to 181.7 per 100,000 persons. However, other means of accounting for disease burden should be used in determining the social and economic impact of the disease. Thus, our main objective is to calculate the burden of NPH for Colombia, South America.

**Methods:** We will use the formal WHO Global Burden of Disease Project developed metric called Disability-adjusted life years (DALY), which incorporates disability and mortality to asses healthy life years lost; as well as the information contained under the ICD-10 code for NPH, within a nationwide database from the Country's health regulation institution (SISPRO), from 2009 to 2015. We will be standardising by age, sex and income based on health insurance regimen.

**Results:** DALYs will be reported as a total estimate, from 2009 to 2015, and as a yearly estimate to establish existing trends. Regional variations are also to be sought.

**Conclusions:** We will be able to provide the latest Country-level assessments of DALY for a specific cause of reversible dementia, providing a basis for future work that will enable a better stratification of the global social, and economic impact of NPH. To our knowledge no previous attempts have been made to calculate disease burden for this specific entity.

## A143

### Obstructive sleep apnea and idiopathic intracranial hypertension syndrome

J. AS Rao, L. Manthripragada, R. Sharma, A. Moghekar

The Johns Hopkins Hospital, Baltimore, MD, USA

**Correspondence:** J. AS Rao

*Fluids and Barriers of the CNS* 2018, **15(Suppl 1)**:A143

**Introduction:** Obesity is a risk factor for both Idiopathic intracranial hypertension syndrome (IIHS) and sleep apnea (SA). Studies linking PTC to Obstructive sleep apnea (OSA) exist in current literature, however these are few, have small sample sizes and only studied male subjects. Lee, A. G., K. Golnik, et al. (2002). Ophthalmology studied 6 men with PTC and OSA who presented with visual abnormalities 5 of whom showed sustained improvement with CPAP treatment. Purvin, V. A., A. Kawasaki, et al. (2000) Arch Ophthalmol performed continuous cerebrospinal fluid pressure monitoring on 4 patients with OSA and Papilledema. They found repeated episodes of marked ICP elevation associated with apnea and oxygen desaturation. They concluded that patients with PTC and OSA may be at increased risk for developing visual loss with nocturnal hypoxemia being an independent risk factor. Through our current study we would like to confirm that there is an association between patients with IIHS and SA in a larger sample. We also plan to explore further the anthropometric characteristics that make subjects with IIHS more susceptible to sleep disordered breathing The clinical implications would be the possibility of including non-invasive treatment methods such as continuous positive airway pressure (CPAP) for the treatment of IIHS. Our hypothesis is that patients with IIHS are more susceptible to SA. Objectives 1. The primary objective is to evaluate for an association between obstructive sleep apnea and IIHS. 2. To identify the underlying clinical characteristics most predictive of SA in patients diagnosed with IIHS.

**Methods:** This is a cross sectional study designed to assess sleep disordered breathing patterns and body anthropometriy in 104 patients diagnosed with IIHS who were evaluated at The Johns Hopkins center for CSF Disorders. The patients undergo routine evaluation for IIHS including lumbar puncture to measure opening pressure and a detailed sleep history and overnight polysomnogram which was scored using standard AASM scoring criteria. The apnea–hypopnea index (AHI) was recorded for both non rapid eye movement (REM) and REM sleep. Independent variables: Opening pressure, Apnea–Hypopnea index, oxygen desaturations. Covariates: We recorded demographic and anthropometric measurements, medical comorbidities and medications.

**Results:** Data from a total of 104 patients was collected and divided into those with and without a diagnoses of SA. The data included CSF pressure and polysomnogram characteristics among patients without and with IIHS (opening pressure = 25) subdivided into those with and without sleep apnea (AHI = 5). Higher opening pressures were found in subjects higher BMI (p 0.001) and SA (p 0.001). We used opening pressure as a measure of disease severity for IIHS and found no significant association between SA severity as measured by AHI. In addition, there was no correlation between opening pressure in those with more severe apnea in REM (0.9) related SA.

**Conclusions:** There have only been a few studies looking at OSA and IIHS. Our sample includes many more variables and a much larger sample size. Our statistical analysis revealed similar results to prior studies after adjusting for age and BMI namely that there is no association between IIHS and OSA. Both conditions however are correlated with age and BMI. Future direction could include looking at multiple time points of direct intracerebral pressure monitoring in conjunction with continuous positive airway pressure use.

## A144

### Sleep disordered breathing and leptin resistance in idiopathic intracranial hypertension syndrome

A. S. Rao, M. Jumaly, D. Solomon, A. Moghekar
The Johns Hopkins Hospital, Baltimore, MD, USA
**Correspondence:** A. S. Rao
*Fluids and Barriers of the CNS* 2018, **15(Suppl 1)**:A144

**Introduction:** Obesity is a risk factor for both Idiopathic intracranial hypertension syndrome (IIHS) and sleep apnea (SA). Thus far, there are no human studies on neurochemical factor that may play a role in the susceptibility of this population to sleep disordered breathing. Leptin is well known for its adipogenic properties (3) and high levels in serum of obese individuals. Animal studies have shown that Leptin is protective against OSA and that it is deficient in the cerebrospinal fluid (CSF) of obese animals, a phenomenon known as "leptin resistance" due to poor penetration through the blood–brain barrier. (4). However, this has not been tested in humans. This project addresses the basic mechanism of OSA susceptibility in obese patients with PTC by distinguishing between those who have leptin resistance (low plasma versus CSF Leptin levels). Through this study we would like to propose alternate interventions such as intranasal leptin as a viable option for the treatment of OSA in this patient population. Our hypothesis is that patients with IIHS are more susceptible to OSA due to obesity and/or leptin resistance. Through this study we plan to explore the relationship between leptin and sleep disordered breathing in subjects with IIHS. We also plan to analyze the CSF from these subjects for evidence of leptin resistance as well as other possible neurohumoral factors which may play a role in the pathophysiology. Objectives: (1) To identify the underlying clinical characteristics of Obstructive Sleep Apnea (OSA) in patients diagnosed with Idiopathic intracranial hypertension syndrome (IIHS). (2) To characterize the relationship between obesity and leptin levels in patients with both IIHS and OSA.

**Methods:** Adults $\geq$ 18 years of age in The Johns Hopkins center for CSF Disorders with a confirmed diagnosis of IIHS who provided informed consent were selected for the study. These patients undergo an evaluation that includes a history (including a sleep and headache questionnaire) and physical exam, lumbar puncture to measure opening pressure and a detailed examination for visual abnormalities. Neuroimaging is obtained as part of the diagnostic workup. For our project we will included a detailed sleep history administered by the Sleep Fellow (including The Epworth sleepiness score and the Hopkins sleep survey) and examination relevant to sleep disordered breathing (including BMI, neck circumference and examination of the oropharynx) to identify those with SA risk factors. Patients who were identified as high probability of having sleep apnea underwent an overnight polysomnogram. The sleep studies were scored using standard AASM scoring criteria. CSF and blood which is obtained as part of routine care was tested for leptin levels in all subjects. Independent variables: Opening pressure, CSF leptin, Plasma leptin, Apnea–Hypopnea index, oxygen desaturations, arousals, and carbon dioxide recordings where available. Covariates: We recorded demographic and anthropometric measurements, medical comorbidities and medications.

**Results:** Data from a total of 18 patients was collected. Higher opening pressures were found in subjects with with higher BMI ($p = 0.03$) and SA ($p = 0.05$). Leptin concentrations in CSF were not correlated to the plasma level ($r = 0.5$; $p = 0.216$). CSF leptin levels were not correlated to body mass index ($r = 0.14$; $p = 0.567$). Opening pressure did not correlate with CSF leptin levels ($r = 0.96$; $p = 0.732$).

**Conclusions:** Obesity is a risk factor for both PTC and OSA and is characterised by leptin resistance due to poor penetration through the blood- brain barrier. Leptin has been shown to be protective against OSA (5). Our pilot data demonstrates that in subjects with IIHS those with higher BMI and SA had higher opening pressures. Our analysis of leptin levels in subjects with IIHS and SA did not demonstrate a correlation between CSF and serum leptin as is seen in obesity and SA, i.e. "leptin resistance". There was also no correlation with BMI which was a surprising finding. This suggests that the neurochemical pathways associated with leptin in IIHS are unique and distinct from its role in adiposity. Sleep disordered breathing and leptin resistance in Idiopathic intracranial hypertension syndrome.

**Summary:** The role of leptin in obesity and in the physiology of respiration has been well established. Patients with sleep apnea have been shown to be "leptin resistant" with low cerebrospinal fluid levels of leptin suggesting abnormal blood brain penetration. Elevated intracerebral pressure has also been associated with sleep disordered breathing. To date no study has looked at the relationship between neurochemical factors and sleep disordered breathing in patients with Idiopathic intracranial hypertension syndrome. We performed analysis of leptin and other adipocytokines in cerebrospinal fluid, reviewed polysomnographic and anthropometric data in patients with IIHS and SA. Our study did not demonstrate "leptin resistance" which suggests a unique role for leptin and the blood brain barrier in this population which is distinct from that seen in adiposity.

## A145

### Adults with hydrocephalus treated in infancy: pathophysiology and pitfalls

H. L. Rekate
Hofstra Northwell School of Medicine, Hempstead, NY, USA
**Correspondence:** H. L. Rekate
*Fluids and Barriers of the CNS* 2018, **15(Suppl 1)**:A145

**Introduction:** A very large percentage of patients who have been treated for hydrocephalus beginning in fetal or early postnatal life must transition to the care of general neurosurgeons when they reach adulthood. This creates problems for both the patient and the new system caring for him or her.
**Methods:** This is a review of the pathophysiology of hydrocephalus in these patients gleaned over 35 years of practice a practice that has treated patients continuously from prenatal diagnosis to adulthood.
**Results:** Adults with hydrocephalus treated in infancy are more difficult to manage that adults who develop hydrocephalus later in life and require and treatment unique to their specific pathology. At least 50% are candidates for ETV at the time of shunt failure. At least 10% will not present with enlarged ventricles despite severely increased intracranial pressure that is sometimes life threatening. Non-responding ventricles are a result of the initial cause of the hydrocephalus being due to venus sinus hypertension and not caused by the shunting of the patient changing the viscoelastic properties of the brain.
**Conclusions**: The care for pediatric patients must include teaching the patient and family about the specific pathophysiology of hydrocephalus in each individual. The care environment for the patient newly caring for such a patient must seek out an understanding of the pathophysiology of that particular patient.

## A146

### LP shunt for INPH patients: surgical techniques and outcomes retrospective single-center study with 348 patients at Tokyo Kyosai Hospital

N. Samejima, N. Kuwana, A. Watanabe, A. Sato, Y. Seki
NPH Center, Department of Neurosurgery, Tokyo Kyosai Hospital, Tokyo, Japan
**Correspondence:** N. Samejima
*Fluids and Barriers of the CNS* 2018, **15(Suppl 1)**:A146

**Introduction:** Two Japanese prospective multicenter cohort studies of treatment of idiopathic normal pressure hydrocephalus (iNPH) were carried out previously. In SINPHONI, treatment was by ventriculoperitoneal (VP) shunt, whereas in SINPHONI-2 it was by lumboperitoneal (LP) shunt. Safety and efficacy were shown in both studies. The present research was a single-center study at our NPH Center, in which iNPH was treated with an LP shunt, and the surgical techniques and treatment outcomes were evaluated.
**Methods:** A total of 348 probable iNPH patients underwent LP shunt surgery between April 2009 and October 2015 (mean age of 78.5 $\pm$ 6.3 years). During the 1-year postoperative follow-up, improvement on the modified Rankin scale (mRS) was evaluated as the primary endpoint; and alleviations of gait disorder, cognitive impairment, and dysuria were evaluated as secondary endpoints.
**Results:** Of the 318 patients followed up at the NPH Center for 1 year after LP shunt surgery, 209 (66; 95% CI 60–71) showed favorable outcomes, defined as improvements of at least one point on the mRS. This proportion was similar to those of 69% (69 of 100; 95% CI 59–78; $p = 0.627$) of all patients treated by VP shunt surgery in SINPHONI, and 63% (52 of 83; 95% CI 51–73; $p = 0.607$) of all patients treated by LP shunt surgery in SINPHONI-2. At 3 months and 1 year after surgery, respectively, the proportions of patients in the present study showing alleviation of gait disorder, defined as improvement by at least 10% in the timed-up-and-go test, were 88.2 and 72.0%; the proportions showing alleviation of cognitive impairment, defined as improvement by at least three points in the mini-mental-state examination, were 56.6 and 43.5%; and those showing alleviation of dysuria, defined as improvement by at least one grade on the iNPH grading scale, were 71.9 and 63.6%.
**Conclusions:** It was important to practice various surgical techniques required for LP shunts, and our LP shunt procedures seem to be generally acceptable from the viewpoint of complications and clinical outcome. Although our relatively high level of success with surgery may be considered to be a minor point, it is worth reporting, as minor differences in technique and know-how can markedly affect the efficacy of shunt surgery.

## A147

### Endoscopic management of hydrocephalus and intracranial cyst. A prospective study of 79 patients

S. Sanoussi, Aminath Kelani, Rabiou Maman Sani, Aminou Sanda, Issa Abdoulwahab, Guemou Addo, Hima Abdoulaye Maiga
Niamey University Niger, Niamey, Niger
**Correspondence:** S. Sanoussi
*Fluids and Barriers of the CNS* 2018, **15(Suppl 1)**:A147

**Objective:** The aim of this study was to evaluate the endoscopic management of hydrocephalus associated with congenital intracranial cyst and to describe the results according to the cyst pressure and open or obstructed acqueduc.
**Methods:** It was a prospective study conducted from January 2015 to May 2017 at Niamey national hospital of Niger. The study included children from 0 to 5 years admitted with hydrocephalus associated with supra or intratentorial cyst without meningitis entecedents. Evaluation of associated supra and infratentorial malformation is based on CT scann analysies. Fenestration of cyst is realised before ETV and endoscopic acqueduc exploration concluded the surgery. Patients were classified into 2 groups. The first group concerned patients with open acqueduc associated with obstructed V4. The second group concerned children with obstructed acqueduc. This second group is divided into two subgroups according to low and high cyst pressure. The follow up evaluation included reduction of cranium perimeter, reduction of Evans index and progression of development of Gezel index during 6 months post operation.
**Results:** The study concerned 79 patients. The mean age was 1.54 months ranged from 1 to 60 months. There were 35 male and 44 female. All patients have intracranial cyst and active hydrocephalus. 48 patients were included with the diagnosis of Dandy Walker malformation, 21 patients with the diagnosis of megacisterna magna, 7 patients with the diagnosis of Dandy Walker variant. Infratentorial cyst were classified as Dandy Walker complex in 52 cases (68.42%). The study included 3 patients with supratentorial third ventricle cyst. 6 months post surgery mean reduction of CP were 2.45 cm, mean reduction of Evans index were 0.0735, mean progression of DQ were 8.79 points. Treatment was successful for 68% of patients. Open acqueduct with obstructed V4 foramen are associated with success. In case of obstructed acqueduc, high pressure cysts are associated with good results.
**Conclusion:** Endoscopic management of intracranial cysts associated with hydrocephalus is efficient. Intracystic high pressure is a predictive factor of success.

## A148

### Neuroanatomical bases of lower psychomotor speed and gait disturbance in idiopathic normal pressure hydrocephalus

S. Sato[1], H. Kazui[1], H. Kanemoto[1], S. Azuma[1], T. Suehiro[1], Y. Suzuki[1], T. Matsumoto[1], K. Yoshiyama[1], H. Kishima[2], M. Ikeda[1]
[1]Department of Psychiatry, Osaka University Graduate School of Medicine, Suita, Japan; [2] Department of Neurosurgery, Osaka University Graduate School of Medicine, Suita, Japan
**Correspondence:** S. Sato
*Fluids and Barriers of the CNS* 2018, **15(Suppl 1)**:A148

**Introduction:** To investigate the neuroanatomical bases of cognitive impairment and gait disturbances in idiopathic normal pressure hydrocephalus (iNPH).
**Methods:** We evaluated clinical features in 33 patients with iNPH (mean [SD] age of 75.0 [5.3] years) before and 3 months after lumboperitoneal shunt surgery (LPS). Cognitive impairment was evaluated with Mini-Mental State Examination (MMSE), Frontal Assessment

Battery (FAB), and Trail Making Test (TMT)-A. Gait disturbance was evaluated with 10-m reciprocating walking test. We counted a time and the number of steps, and calculated average gait speed, average step length, and cadence. Regional cerebral blood flow (rCBF) was quantified in 34 Regions-of-interest (ROIs) by 123I-IMP single photon emission computed tomography using the autoradiography method. We assessed the relationships between improvements of clinical measures and improvement of rCBF of each ROI after LPS.

**Results:** There were significant improvements of MMSE, FAB, gait speed, step length, and cadence after LPS. The rCBF in the left anterior thalamus, bilateral amygdala and left hippocampus significantly improved after LPS. We found significant associations between an improvement of TMT-A and increased rCBF in the left anterior thalamus, between an improvement of gait speed and increased rCBF in the left anterior thalamus and left amygdala, and between an improvement of step length and increased rCBF in the left ventrolateral prefrontal cortex, left putamen and left amygdala.

**Conclusions:** These results might suggest that hypoperfusion in the anterior thalamus is responsible for decreases of psychomotor speed and gait speed, and that hypoperfusion in the amygdala relates to gait disturbance.

## A149

### Hydrocephalus and frailty

E. A. Schmidt[1], Z. Czosnyka[3], L. Balardy[2]
[1]Department of Neurosurgery, Toulouse University Hospital, Toulouse, France; [2]Department of Geriatry, Toulouse University Hospital, Toulouse, France; [3]Brain Physics Lab, Academic Neurosurgery, University of Cambridge, Cambridge, UK
**Correspondence:** E. A. Schmidt
*Fluids and Barriers of the CNS* 2018, **15(Suppl 1)**:A149

**Introduction:** Frailty is a common clinical syndrome best conceived as an acceleration of the aging process. Frailty in elderly persons increases their vulnerability to stress, thus leading to a greater risk of disease and disability, increased use of health services and a higher risk of death. Normal pressure hydrocephalus (NPH) is well known as a treatable syndrome of the classical triad of gait disturbance, dementia, and urinary disturbance. This work is the first attempt in order to discuss hypotheses on the link between frailty and NPH, a new window of opportunity for reversible frailty. Our framework should successively address whether (i) patients suspected of NPH meet frailty criteria, (ii) hydrocephalus aggravates frailty and (iii) in what way treating NPH with a shunt insertion should be considered as reversing frailty.

**Methods:** The objective of this work was to estimate prospectively prevalence of frailty in a cohort of 100 patients suspected of NPH. In all patients, we measured CSF hydrodynamics, in particular resistance to CSF outflow (Rout). We also measured frailty, with the Short Emergency Geriatric Assessment (SEGA) index, a simple tool to detect frailty in elderly subjects. SEGA index evaluates frailty on a 13-item scale, with each item graded either 0 (most favourable state), 1 or 2 (least favourable state), thus making it possible to classify subjects into three groups: not very frail (score $\leq 8$), frail ($8 < \text{score} \leq 11$), and very frail (score $> 11$).

**Results:** In our cohort, mean $\pm$ SD SEGA was $8.9 \pm 3.6$. 63% of were not very frail (SEGA $\leq 8$) and 19% very frail (SEGA $> 11$). There no correlation between SEGA index and Rout (cf figure). But note that patients with the lowest SEGA (less frail) were those with the lowest Rout (less CSF disorder).

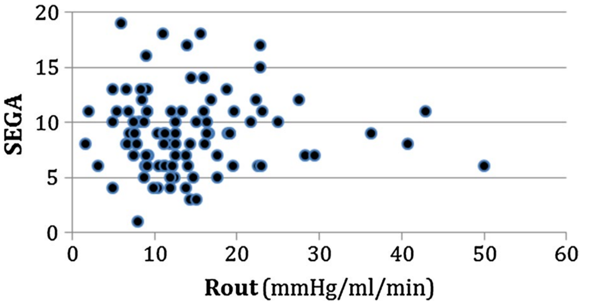

**Conclusions:** More than half of our population suspected of NPH meets frailty criteria. The concept of frailty, a precursor state to functional dependency, appears as a useful tool in gaining better knowledge of the mechanisms leading to loss of autonomy. Identification of frailty has become a major objective, notably with the aim of implementing effective preventive health policies, targeting populations at risk of dependency sufficiently upstream in the disablement process. Identification of frail patient with NPH in order to be treated with a shunt should valuable to reverse loss of autonomy and improve quality of life, but also be beneficial in terms of health economics, health system efficiency but also in terms of social protection.

## A150

### Influence of the lumbar needle intrinsic resistance on csf hydrodynamic parameters during infusion tests

I. Djidjeli[1], M. Niare[1], Z. Czosnyka[2], S. Lorthois[3], P. Swider[3], E. A. Schmidt[1,3,4]
[1]Department of Neurosurgery, University Hospital, Toulouse, France; [2]Brain physics lab, Academic Neurosurgery, University of Cambridge, Cambridge, UK; [3]Institute of Fluid Mechanics of Toulouse, Toulouse, France; [4]INSERM TONIC 1014, Toulouse, France
**Correspondence:** E. A. Schmidt
*Fluids and Barriers of the CNS* 2018, **15(Suppl 1)**:A150

**Introduction:** The fluid pressure in the subarachnoid space is distributed evenly in the cranium and the spine thanks to Pascal Swider's law. In a supine patient (i.e. low gradient effect of gravitation on body fluids) with communicating CSF circulation, a needle inserted in the lumbar space gauges cranio-spinal fluid. During infusion tests, saline is injected though a lumbar needle to challenge intracranial fluid mechanics. The lumbar needle has per se an intrinsic resistance that has to be taken in account to measure accurately CSF hydrodynamic parameters. To avoid this, one proposes to insert two lumbar needles: one for fluid pressure measurement and one for fluid challenge. Hence the measurement is not influenced by the needle's resistance. However, it imposes the insertion of a second needles that increases the morbidity and duration of the procedure. One needle infusion study is routinely performed in our Department. We hypothesize that the lumbar needle's resistance influences the resistance to CSF outflow (Rout) but doesn't correlate with other intracranial parameters.

**Methods:** 100 one needle lumbar infusions studies have been performed. ICM+ software was used to measure and estimate various parameters such as baseline and plateau ICP mean, ICP pulse amplitude (Amp), resistance of the needle, Rout (taking in account the needle's resistance), elastance and PVI. We performed basic statistics and Pearson's $r^2$ correlation between the needle's resistance and other intracranial parameters.

**Results:** In this example of infusion study, the needle's resistance is identified by the first bump in the upper left trace. Note that the change in ICP mean doesn't influence ICP pulsatility (Amp).In our group, the mean ($\pm$ SD) resistance of the needle was $1.32 \pm 0.59$ mmHg $ml^{-1}$ $min^{-1}$. There was no correlation (i.e. $r^2 \approx 0$) between the resistance of the needle vs. baseline ICP, plateau ICP, baseline ICP amp, plateau ICP amp, Rout, elastance and PVI.

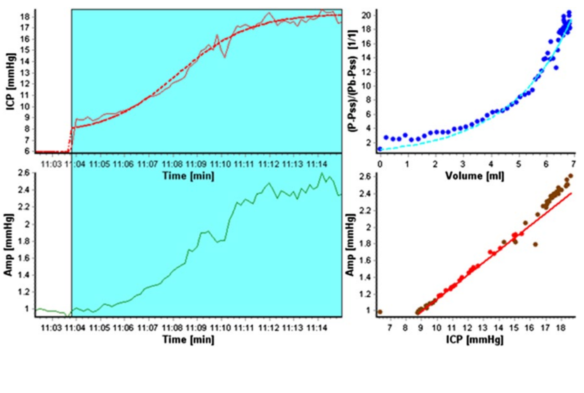

**Conclusions:** We didn't identify any correlation between the needle's resistance and other hydrodynamics parameters. During infusion studies, the resistance of the needle is roughly 1.5 mmHg $ml^{-1}$ $min^{-1}$ and has to be taken in account to measure accurately Rout. One lumbar needle infusion study seems appropriate to gauge intracranial hydrodynamic parameters.

## A151

### Computational analysis of the effect of posture on cerebral hemodynamics and intracranial pressure

Eun Bo Shim[1,2], Thomas Heldt[2]
[1]Department of Mechanical and Biomedical Engineering, Kangwon National University of Technology, Chuncheon, Republic of Korea; [2]Institute of Medical Engineering and Science, Massachusetts Institute of Technology, Cambridge, MA, USA
**Correspondence:** Eun Bo Shim
*Fluids and Barriers of the CNS* 2018, **15(Suppl 1)**:A151

**Introduction:** Intracranial pressure (ICP) and cerebral venous hemodynamics are significantly affected by posture changes. As one transitions from the supine to the upright posture, ICP (as measured in the ventricular system) falls, the jugular veins collapse, and cerebral venous outflow is thought to be primarily mediated via the vertebral system.
**Methods:** We built developed a computer simulation model to understand how arterial blood pressure, jugular venous pressure, and ICP interact to drive cerebral blood flow during posture change. A lumped parameter model of the cerebrospinal fluid (CSF) compartment and the arterial and venous vascular systems was implemented. For the validation of the present method, computed ICP and venous hemodynamic variables were compared to head-up experiments in the medical literature. We also investigated physiological variations such as the effect of collapse and reopening of jugular veins induced by the application of Valsalva maneuver during posture change.
**Results:** Using the proposed model, we simulated the physiological responses of the CSF compartment and jugular and vertebral venous flows during transitions in posture. Maintenance of cerebral blood flow during the changes in posture depends on the interplay of changes in ICP and venous pressures.
**Conclusions:** Computer simulations greatly aid in understanding the biophysics of the cerebrospinal fluid and cerebral and extra-cerebral vascular compartments.

## A152

### Withdrawn

*Fluids and Barriers of the CNS* 2018, **15(Suppl 1)**:A152

## A153

### Assessment of cerebral blood flow via spectral analysis of cerebrospinal fluid pressure acquired during infusion test

E. S. Song[1], H. Kim[1], I. N. Wang[1], Y. T. Kim[1], H. J. Lee[1], H. H. Yoo[2], Z. Czosnyka[3], M. Czosnyka[3], D. J. Kim[1]
[1]Department of Brain and Cognitive Engineering, Korea University, Seoul, South Korea; [2]Department of Biomedical Engineering, Korea University, Seoul, South Korea; [3]Department of Neurosurgery, Addenbrooke's Hospital, University of Cambridge, Cambridge, UK
**Correspondence:** E. S. Song
*Fluids and Barriers of the CNS* 2018, **15(Suppl 1)**:A153

**Introduction:** A decrease in cerebral blood flow (CBF) is repeatedly observed in patients with severe symptoms of normal pressure hydrocephalus (NPH), however it is currently unclear how exactly a decrease in CBF affects the symptoms of NPH. A previous study reports that CBF is highly associated with the low-frequency centroid (LFC) of intracranial (or cerebrospinal fluid; CSF) pressure signal. This study attempted to investigate the association between CBF decrease, assessed by LFC, and the symptom presentation in NPH.
**Methods:** The clinical information and data from forty-two NPH patients who had undergone CSF infusion test were retrospectively analyzed. The LFC was obtained from spectral analysis of low frequency bandwidth (0.5–2.5 Hz). Major symptoms of NPH is defined as per the Hakim triad (i.e. gait disturbance, cognitive deterioration and urinary incontinence).
**Results:** Urinary incontinence ($n = 6$) was highly associated with lowered LFC during the entire phase of the infusion test ($p < 0.05$). The lowered LFC in patients with urinary incontinence was most prominent during the plateau phase of infusion test ($p = 0.001$, AUC = 0.912), followed by transient phase ($p = 0.024$, AUC = 0.792) and the baseline phase ($p = 0.048$, AUC = 0.755). The lowered LFC was not indicative of cognitive deterioration ($n = 14$) nor gait disturbance ($n = 19$).
**Conclusions:** The urinary incontinence, which is known to be associated with lowered CBF, is also associated with lowered LFC. Correcting CBF could be effective for urinary incontinence, but not for the cognitive and gait dysfunction in NPH. Efforts should be made for combining different therapeutic options for treating varying symptoms of NPH.

## A154

### ICP pulse amplitude modulation by respiration is increased in aqueduct stenosis

A. Spiegelberg[1], M. Krause[2], J. Meixensberger[2], V. Kurtcuoglu[1,3]
[1]University of Zurich, The Interface Group, Institute of Physiology, Zurich, Switzerland; [2]Universitaetsklinikum Leipzig AoeR, Klinik und Poliklinik für Neurochirurgie, Leipzig, Germany; [3]University of Zurich, Zurich Center for Integrative Human Physiology and Neuroscience Center, Zurich, Switzerland
**Correspondence:** A. Spiegelberg
*Fluids and Barriers of the CNS* 2018, **15(Suppl 1)**:A154

**Introduction:** Respiration modulates ICP pulse amplitude originating in cardiac beat-induced vascular pulsation ($A_{vp}$). This modulation reflects the change in craniospinal compliance during a respiratory cycle. We aimed to quantify such modulation during periods of impaired compliance as characterized by the occurrence of B-waves, and to test whether the amplitude modulation is increased in aqueduct stenosis.
**Methods:** We developed a method for quantifying $A_{vp}$ while demodulating respiratory waves. With $A_{rp}$ denoting the amplitude of respiratory waves in the ICP time course, and $AA_{vp}$ the amplitude of respiratory waves in the $A_{vp}$ time course, we defined 'Respiratory Amplitude Quotient' as $RAQ = A_{rp}/AA_{vp}$. We determined RAQ in phases of B-wave occurrence in recordings of two patient groups presenting with Hakim's triad: 17 patients without positive response to external lumbar drainage or irresponsive to shunt therapy (non-responder, NR), and seven patients with aqueduct stenosis (AQ) confirmed by morphological changes assessed in MRI scans. We used a Mann–Whitney test to determine whether there was an association between RAQ and the diagnosis.
**Results:** We found significant association between RAQ and independent AQ diagnosis ($p = 0.0308$) with RAQ lower in the AQ group. Our method proved suitable for quantifying respiratory modulation of ICP pulse amplitude originating in vascular pulsation.
**Conclusions:** In AQ patients, respiration modulates the vascular ICP pulse amplitude significantly stronger than in NR patients. This modulation may reflect short term craniospinal compliance, which appears to be substantially reduced in aqueduct stenosis.

## A155

### Treatment and long-term survival: subdural hematomas in 1846 shunted INPH patients

N. Sundström[1], M. Lagebrant[2], A. Eklund[1], L.-O. D. Koskinen[2], J. Malm[2]
[1]Department of Radiation Sciences, Biomedical Engineering, Umeå University, Umeå, Sweden; [2]Department of Pharmacology and Clinical Neuroscience, Umeå University, Umeå, Sweden
**Correspondence:** N. Sundström
*Fluids and Barriers of the CNS* 2018, **15(Suppl 1)**:A155

**Introduction:** A serious adverse event following shunt surgery is subdural hematoma (SDH), but the use of adjustable shunts makes it possible to non-invasively treat postoperative SDH. In this study, based on a very large patient cohort, the prevalence of fixed or adjustable shunt valves, treatment preferences and effect of SDH and treatment on long-term survival were investigated.
**Methods:** Patients with idiopathic normal pressure hydrocephalus (INPH) and operated on with a cerebrospinal fluid shunt in Sweden 2004–2015 were included in a prospective quality registry (n = 1846) and followed regarding SDH, its treatment and long-term survival. The treatment of SDH was categorized into surgery, opening pressure adjustments or no treatment.
**Results:** The proportion of adjustable shunts increased from 77% to 93% between 2004 and 2015. 184 patients (10%) developed an SDH. Opening pressure adjustments, surgical treatment and no treatment was applied in 103 (56%), 66 (36%) and 15 (8%) cases respectively. Surgical treatment of SDHs was used in 90% (n = 17) of patients with fixed shunt valves, whereas in 30% (n = 49) of patients with adjustable ($p < 0.001$). There was no difference in long-term survival between SDH and non-SDH groups, or between different treatments.
**Conclusions:** After shunt surgery, SDH is still a common complication. Adjustable shunts reduced the need for surgical interventions, and survival was not affected by the occurrence of an SDH or by method of treatment. Thus, today adjustable shunts offer a non-invasive treatment option which considerably reduces the level of severity for this common adverse event.

## A156

### Assessment of cardiac- and respiratory-induced pressure gradients of CSF based on real time magnetic resonance imaging

S. Sunohara[1], S. Yatsushiro[2], T. Tokushima[1], M. Matsumae[3], H. Atsumi[3], K. Kuroda[1,2]
[1]Graduate School of Engineering, Tokai University, Hiratsuka, Kanagawa, Japan; [2]Graduate School of Science and Technology, Tokai University, Hiratsuka, Kanagawa, Japan; [3]Department of Neurosurgery, Tokai University School of Medicine, Isehara, Kanagawa, Japan
**Correspondence:** S. Sunohara
*Fluids and Barriers of the CNS* 2018, **15(Suppl 1)**:A156

**Introduction:** To assess and classify the cerebrospinal fluid (CSF) dynamics, cardiac- and respiratory-induced pressure gradients were visualized and quantified in healthy subjects using real-time magnetic resonance (MR) imaging.
**Methods:** Asynchronous 2D phase-contrast imaging was applied to 12 healthy subjects (10 males and 2 females, 31 ± 13 yo) at 3-tesra MRI to observe spatio-temporal distributions of total CSF velocities. Subjects were instructed to repeat 6-s respiration by audio guidance. Cardiac pulsation of each individual was recorded by electrocardiogram. Whereas respiration was monitored by a bellows-type pressure sensor. The frame rate was 4.6 image/s, resulting in acquiring approximately 256 frames during 56 s. The total velocity waveform in each voxel was separated into the cardiac- and respiratory-driven components in the frequency domain. The two components were then used to calculate the corresponding pressure gradients based on Navier–Stokes equation. After obtaining the pressure gradient components in all the voxels in the intracranial CSF space, region of interests (ROIs) were placed at the anterior cistern of the brainstem, Sylvian aqueduct, lateral ventricle, and fourth ventricle for quantitative analysis.
**Results:** The cardiac-driven pressure gradient in caudal-to-cranial was 14.2 ± 3.05 Pa/m, while the respiratory-driven was 1.23 ± 0.46 Pa/m at the anterior cistern of the brainstem. The cardiac component was significantly and consistently higher than the respiratory in all the ROIs.
**Conclusions:** The cardiac- and respiratory-driven CSF pressure gradients in the intracranial space were differentiated. As the scan time is only a minute, the present technique is readily applicable to patients with hydrocephalus to be compared with the healthy subjects.

## A157

### Computerized tomographic study of normative values of Evans' index, lateral ventricular volume and skull shape in thai adults

T. Jewrasamnuay[1], T. Supsamanwong[1], T. Nanthanasub[1], T. Wittiwej[2], O. Chawalparit[3], C. Ngamsombat[3]
[1]Faculty of Medicine Siriraj Hospital, Mahidol University, Bangkok, Thailand; [2]Division of Neurosurgery, Department of Surgery, Faculty of Medicine, Siriraj Hospital, Mahidol University, Bangkok, Thailand; [3]Division of Diagnostic Radiology, Department of Radiology, Faculty of Medicine Siriraj Hospital, Mahidol University, Bangkok, Thailand
**Correspondence:** T. Jewrasamnuay and T. Supsamanwong
*Fluids and Barriers of the CNS* 2018, **15(Suppl 1)**:A157

**Introduction:** Idiopathic normal pressure hydrocephalus (iNPH) is non-obstructive ventricular enlargement with normal intracerebral pressure resulting in a triad, gait difficulty, cognitive impairment and urinary incontinence. The Evans' index (EI) has been extensively used as diagnostic imaging. The EI value of 0.3 or greater is one of criteria in iNPH guideline. However, the normal EI in the Thai aging population has not been reported. Moreover, skull shapes between the Asian and Caucasian may be different. The purpose of the study is to establish normal Evans' index, lateral ventricular volume (LVV) and the correlation respect to age, modified cephalic index (mCI) in non-hydrocephalus Thai adults.
**Methods:** Axial brain non-contrast computerized tomographic scans (NCCT) of 100 Thai mild traumatic head injury subjects (45 men, 55 women) without intracranial lesion were reviewed. The mean age was 67.25 ± 15.38(above 40 years old). EI, LVV, mCI were measured.
**Results:** The mean value of EI in this study was 0.258 ± 0.03. The correlation coefficient of EI, LVV with respect to Age is high in 70–79 and ≥ 80 age group with the value of 0.611, 0.632 respectively. The mean value of mCI was 86.8 ± 4.5 and compatible with hyperbrachycephalic head shape.
**Conclusions:** EI is a good indirect marker of LVV especially in patients above 70 years old. Different head shape between Caucasians and Thais may reduce optimal EI cutoff point.

## A158

### Chronic posttraumatic headache completely cured by ventriculo-atrial shunt: report of three cases

K. Takagi[1], K. Onouchi[2], K. Kato[3]
[1]NPH Center, Kashiwa-Tanaka Hospital, Japan; [2]Department of Neurology, Kashiwa-Tanaka Hospital, Kashiwa, Japan; [3]Department of Surgery, Abiko-Seijinkai Hospital, Abiko, Japan
**Correspondence:** K. Takagi
*Fluids and Barriers of the CNS* 2018, **15(Suppl 1)**:A158

**Introduction:** Chronic post-traumatic headache (CPTH) is still a common complication of minor head injuries. The purpose of this study is to report three cases with long lasting CPTH completely cured by VA shunt.
**Methods:** We have treated 728 patients with CPTH. Most of them complained headache with orthostatic nature similar to spontaneous intracranial hypotension. However, their CSF pressures were not low and their signs and symptoms improved by CSF removal in high incidence. Therefore, we applied VA shunt in 16 cases. Three cases with complete cure were reported here.
**Results:** Case 1 was 66 yo male who suffered from whiplash injury 5 years ago. He complained orthostatic headache with many other symptoms such as memory disturbance. He received VA shunt because CSF removal improved his signs and symptoms. All of his symptoms disappeared next morning of the surgery. Case 2 was 68 yo female with orthostatic headache and other many symptoms shortly after whiplash injury 17 years ago. Because her complaints almost disappeared by CSF removal, VA shunt was applied. Her symptoms almost disappeared next morning of the surgery. Case 3 was 64 yo complaining orthostatic headache with body pain similar to fibromyalgia since whiplash injury 24 yo ago. She received VA shunt because CSF removal eliminated her signs and symptoms. Her body pain disappeared just next morning of the surgery

**Conclusions:** These positive effects of VA shunts for CPTH without organic brain damage suggest that the involvement of CSF circulation abnormalities for the development of CPTH. Informed consent to publish had been obtained from the patients.

## A159

### Regional cerebral blood flow at the top of high convexity is not increased in idiopathic normal pressure hydrocephalus

R. Takahashi, K. Ishii, T. Tokuda, M. Nakajima, T. Okada
Department of Neurology, Hyogo Prefectural Rehabilitation Hospital at Nishi-Harima, Tatsuno, Hyogo, Japan
**Correspondence:** R. Takahashi
*Fluids and Barriers of the CNS* 2018, **15(Suppl 1)**:A159

**Introduction:** The purpose of this study was to elucidate the specific regional cerebral blood flow (rCBF) alterations for idiopathic normal pressure hydrocephalus (iNPH) by comparing proportional rCBF and grey matter change from those of a normal database at the same point of SPECT and MRI examinations, and to demonstrate the relationship between rCBF and the callosal angle.

**Methods:** Thirty subjects with iNPH underwent both CBF SPECT and MRI. After normalization, voxel-wise two-samples t test between patients and 11 normal controls were conducted to compare the regional alteration in grey matter density and rCBF in addition to the correlation analysis between rCBF and the callosal angle.

**Results:** The rCBF reduction and the grey matter decrease were seen in almost similar regions surrounding Sylvian fissure, the left parieto-temporal lobe and frontal lobes whereas we did not find rCBF increase at the top of the high convexity where the increase of grey matter density was the highest. Further, the significant positive correlation between rCBF and the callosal angle was found at the top of the high convexity.

**Conclusions:** The current study suggests the relative rCBF decrease due to compression of the top of the high convexity in iNPH. The absence of the rCBF increase with evident increase of grey matter density at the top of the high convexity might be a hallmark of iNPH.

## A160

### Unorthodox findings in magnetic resonance imaging could suggest better treatment outcome in idiopathic intracranial hypertension patients

J. Tervonen, J. Harju, R. Vanninen, V. Leinonen, A-P. Elomaa, T. Puustinen, J. E. Jääskeläinen, T. Huttunen
Kuopio University Hospital, Kuopio, Finland
**Correspondence:** J. Tervonen
*Fluids and Barriers of the CNS* 2018, **15(Suppl 1)**:A160

**Introduction:** The idiopathic intracranial hypertension (IIH) a rare and etiologically unknown disorder characterised by serious disturbances in cerebrospinal dynamics, obesity and hormonal factors. In adult populations, the IIH mostly affects obese woman of childbearing age. The IIHs conventional radiographic manifestations are non-specific, as guided by modified Dandy criteria, although several suggestive MRI findigs have been presented. Thus, we studied unorthodox magnetic resonance imaging (MRI) findings and their correlations to BMI, cerebrospinal fluid opening pressure (OP) and treatment outcome.

**Methods:** A total of 35 patients with IIH diagnosis admitted defined catchment area between 2000 and 2016. All available demographic, clinical, medical charts and imaging findings were studied.

**Results:** The mean age at the time of diagnosis was 30 years and the mean follow-up time was 4.4 years and 80% were females. At diagnosis, mean BMI was 36.2 kg/m$^2$ and mean OP was 31.2 mmHg. Treatment included acetazolamide (94%) and shunting (34%). The MRI at diagnosis showed partial or complete empty sella in 48.6%, edema of optic nerve (CNII) sheaths in 45.7%, increased tortuosity of CNII in 17.1%, flattened sclera in 28.6%, and intraocular protrusion of CNII head in 8.6% of cases. The presence of empty sella, increased tortuosity and edema of CNII, and flattened sclera correlated with higher OP at presentation, although the differences were not significant. The BMI at presentation was higher in patients with empty sella. Complete resolution of symptoms was found more often in patients with IIH-related MRI-findings (78% vs. 56%)

**Conclusions:** Unorthodox findings in MRI are common in IIH patients. The IIH patients with empty sella on MRI had higher BMI and OP at diagnosis, whereas more severe MRI findings suggested better treatment outcomes. Unorthodox phenotypes such as presentation of empty sella are frequent in IIH and could contribute on neuroendocrinological disturbances, thus further multidisciplinary research is warranted.

## A161

### Cost versus benefit analysis of telemetric ICP measuring device within a shunt system: a single centre experience

S. D. Thompson, L. D'Antona, L. W. Thorne, A. K. Toma, L. D. Watkins
The National Hospital for Neurology and Neurosurgery, London, UK
**Correspondence:** S. D. Thompson
*Fluids and Barriers of the CNS* 2018, **15(Suppl 1)**:A161

**Introduction:** The recent introduction of telemetric intracranial pressure (ICP) monitoring devices presents an opportunity to improve the treatment of patients post shunt insertion. However, these devices are not without an increase in cost, which presents a challenge to many public health services to assess the financial value of inserting such devices.

**Methods:** A retrospective review of our in-house database of all Miethke Sensor Reservoir (SR) units inserted to date. We look at the number of occasions that patients with these devices have presented, when without this device, they would have been admitted for surgical insertion of an ICP bolt in line with our hospital policy. The SR currently costs around an additional £1700 versus our standard shunt reservoir, with an average cost of admission/surgery of £3000 for ICP monitoring at our institution.

**Results:** 60 SR units inserted to date over a 6.5 year period, telemetrically measured ICP 105 times. A minimum of 18 ICP bolts have been prevented in this patient population due to the SR unit, resulting in a return on investment (ROI) of 53%. ICP bolts prevented in patients with an SR in place for > 6 months (n33) = 16, with an ROI of 86%. ICP bolts prevented in patients with an SR in place for > 12 months (n9) = 7, with a ROI of 137%.

**Conclusions:** Our results suggest a cost–benefit after 12 months for the SR. We believe this highlights that there is a financial benefit to using the SR as well as a clinical one.

## A162

### The effect of MRI scans on implanted shunt valves with magnetically adjustable settings

S. D. Thompson, L. W. Thorne, L. D. Watkins, A. K. Toma
The National Hospital for Neurology and Neurosurgery, London, UK
**Correspondence:** S. D. Thompson
*Fluids and Barriers of the CNS* 2018, **15(Suppl 1)**:A162

**Introduction:** The use of magnetically adjustable valves for the treatment of complex hydrocephalus has been increasing gradually since the original Hakim adjustable valve was introduced in the 1980s. However the issue of magnetic forces unintentionally adjusting valves has caused issues over the years with unintentional adjustments through contact with magnetic fields causing patients to deteriorate and require intervention. Some valves available to market, whilst being unable to be classed as 'MRI safe' due to incorporating magnets, are understood to be unlikely to adjust in an MR environment.

**Methods:** Over a 5 month period, an attempt was made to check the shunt setting pre/post MRI for all patients undergoing an MRI with an adjustable shunt. Checks were made pre and post MRI by the same highly experienced practitioner.

**Results:** Over the course of 5 months, 28 patients were assessed with a magnetically adjustable valve. 10 patients had a Miethke Pro-GAV

2.0, 5 Miethke Pro-GAV, 2 a Miethke Pro-SA, 3 Codman Certas, 3 Codman Certas Plus and 3 Medtronic Strata and 2 Medtronic Strata NSC. No Miethke or Certas valves were found to adjust in either a 3 T or 1.5 T scan. All strata valves were found to adjust.
**Conclusions:** Our small sample size suggests that the Miethke Pro-GAV, Pro-GAV 2.0, Pro-Sa and the Codman Certas Plus are reliable and have not demonstrated interpedently adjusting in an MR environment.

## A163
### A single centre experience of shunted women who experience pregnancy/childbirth: patient feedback, neurosurgical interventions and obstetric advice
S. D. Thompson, L. D'Antona, J. Funnell, A. Chakravorty, V. Wykes, L. W. Thorne, A. K. Toma, L. D. Watkins
The National Hospital for Neurology and Neurosurgery, London, UK
**Correspondence:** S. D. Thompson
*Fluids and Barriers of the CNS* 2018, **15(Suppl 1)**:A163

**Introduction:** Due to advances in medicine, shunt technology and surgical outcomes, more patients are living longer and improved quality of life with CSF shunts. As a result, more women are now becoming pregnant with a CSF shunt in place. Advice for patients/obstetricians varies in different departments regarding method of delivery and likely management options
**Methods:** A retrospective review of all women who underwent a CSF shunt between 2002 and 2017 aged between 18 and 55 was undertaken. All case notes were reviewed and patients contacted to discuss their experience of pregnancy/childbirth with a shunt in place as part of a service evaluation.
**Results:** 28 patients were identified with 33 recorded pregnancies post shunt insertion. 69% of patients underwent caesarean section delivery, 50% of which are documented as under GA. Patients required a range of treatments during pregnancy to manage signs of increased ICP ranging from no intervention/shunt valve adjustment to externalisation of peritoneal shunt tubing. On qualitative questioning, patients described faith in both neurosurgical and obstetric teams during pregnancy but highlighted the importance of communication between the two teams.
**Conclusions:** Both natural, assisted and caesarean section can be undertaken in women of childbearing age with the appropriate method and birth plan decided on between the patient, neurosurgeon and obstetrician. Communication between teams is important to improve the experience of patients.

## A164
### A new implantable telemetric intracranial pressure monitoring device within a shunt system: a single centre experience of 60 implanted units over a 6.5 year period
S. D. Thompson, L. D'Antona, L. W. Thorne, A. K. Toma, L. D. Watkins
The National Hospital for Neurology and Neurosurgery, London, UK
**Correspondence:** S. D. Thompson
*Fluids and Barriers of the CNS* 2018, **15(Suppl 1)**:A164

**Introduction:** The role of intracranial pressure (ICP) monitoring in assessing complex hydrocephalus patients has long been understood. Elective ICP monitoring via an ICP bolt is undertaken in multiple centres internationally, to assess for possible shunt dysfunction. The Miethke Sensor Reservoir (SR) allows for telemetric, non-invasive, measuring of ICP once inserted within a shunt system.
**Methods:** A retrospective review of a prospectively built database of all patients with an implanted SR. All available clinical notes and patient outcomes were assessed.
**Results:** 60 SR units inserted over a 6.5 year period, Median length of implantation 6.5 months (mean 9.4, range 63–1). No direct complications experienced from SR. Pressure measured via SR on 105 documented cases which has resulted in 50 shunt adjustments, 53 occasions resulting in no further neurosurgical intervention and 2 occasions where shunt revision surgery was required. In-vivo real time comparison with conventional intraparenchymal bolt undertaken on 2 separate patients. Shunts inserted for 18 months and 1 day respectfully, results demonstrated less than 1 mmHg difference in 90° position.
**Conclusions:** The Miethke SR is a reliable tool to guide management of complex hydrocephalic patients. It has allowed for quick, low risk assessment of patients which has helped to shape their further management. In-vivo studies demonstrate accurate readings up to 18 months post implantation.

## A165
### Withdrawn
*Fluids and Barriers of the CNS* 2018, **15(Suppl 1)**:A165

## A166
### Skull shape as a risk factor for hydrocephalus among children with premature suture fusion
H. M. Tully[1,2,3], M. Pet[4], T. Wenger[5], A. Lee[6], C. Birgeld[5], W. B. Dobyns[1,3,8], R Hopper[5]
[1]Division of Pediatric Neurology, University of Washington, Seattle, WA, USA; [2]Institute for Translational Health Sciences, University of Washington, Seattle, WA, USA; [3]Center for Integrative Brain Research, Seattle Children's Research Institute, Seattle, WA, USA; [4]Division of Plastic and Reconstructive Surgery, University of Washington, Seattle, WA, USA; [5]Division of Craniofacial Medicine, University of Washington, Seattle, WA, USA; [6]Department of Neurosurgery, Seattle, WA, USA; [7]Department of Pediatrics, University of Washington, Seattle, WA, USA; [8]Division of Medical Genetics, University of Washington, Seattle, WA, USA
**Correspondence:** H. M. Tully
*Fluids and Barriers of the CNS* 2018, **15(Suppl 1)**:A166

**Introduction:** We recently demonstrated that brain/skull configuration is associated with hydrocephalus in various clinical situations. To delineate what aspects of skull shape contribute to hydrocephalus, we assessed children with quantifiable cranial abnormalities associated with premature suture fusion.
**Methods:** We performed a retrospective cohort study of children treated for multisuture synostosis at a regional craniofacial center between 2005 and 2015, reviewing clinical records, quantifying skull measurements from 3D CTs, and calculating Cranial Index (CI) and Point of Maximum Width (PMW). We used Chi squared and t-tests to compare outcome groups on the basis of clinical and anatomical characteristics, and Poisson regression to generate adjusted risk estimates.
**Results:** 15 (12%) of 122 children with multisuture synostosis developed hydrocephalus. Among those who underwent posterior cranial vault expansion (PCVE), those who did so before anterior expansion had a markedly reduced risk of hydrocephalus (aRR: 0.08; 95% CI: 0.02, 0.28). No significant differences were seen in pre-surgical CI or PMW, but post-surgical PMW was 8% lower in children who developed hydrocephalus than those who did not ($p = 0.004$). Notably, no child who underwent PCVE with distraction osteogenesis (PCVE-DO) subsequently developed hydrocephalus. Concordantly, children who underwent PCVE-DO had a 5% higher post-surgical PMW than those who underwent PCVE alone ($p = 0.03$).
**Conclusions:** Among children with multisuture synostosis, the posterior cranial vault influences the development of hydrocephalus. Early posterior vault expansion and distraction osteogenesis (which may result in greater expansion) are both associated with reduced risk. Whether posterior vault configuration plays a role in other forms of hydrocephalus deserves further study.

## A167
### Risk factors for post-hemorrhagic hydrocephalus among infants with intraventricular hemorrhage
H. M. Tully[1,2,3], T. Rue[4], C. Traudt[5], B. Saltzman[6], A. Oron[6], T. D. Simon[6,7], W. Kukull[8], D. Doherty[2,7]
[1]Division of Pediatric Neurology, University of Washington, Seattle, USA; [2]Institute for Translational Health Sciences, University of Washington, Seattle, WA, USA; [3]Center for Integrative Brain Research, Seattle Children's Research Institute, Seattle, WA, USA; [4]Department of Biostatistics, University of Washington, Seattle, WA, USA; [5]Division of Neonatology, University of Washington, Seattle, WA, USA; [6]Center for Clinical and Translational Research, Seattle Children's Research Institute, Seattle, WA, USA; [7]Department of Pediatrics, University of Washington, Seattle, WA, USA; [8]Department of Epidemiology, University of Washington, Seattle, WA, USA
**Correspondence:** H. M. Tully
*Fluids and Barriers of the CNS* 2018, **15(Suppl 1)**:A167

**Introduction:** Post-hemorrhagic hydrocephalus (PHH) is a common complication of intraventricular hemorrhage (IVH). Although risk factors for IVH have been investigated and clinical care has changed as a result, less is known about risk factors for PHH.
**Methods:** Retrospective cohort study using the Pediatric Hospital Information System database. Cox models were used to calculate adjusted Hazard Ratios (HRs) and 95% confidence intervals (CIs) for risk factors present prior to treatment for PHH.
**Results:** Among 20,877 infants with IVH, 2421 (11.6%) developed PHH. Most (58%) of the study population was male, but male sex was not associated with an increased risk of PHH. In univariate analyses, Hispanic and Asian ethnicity were associated with a reduced risk of PHH compared to whites (HR 0.8, 95% CI 0.7, 0.9; HR 0.5, 95% CI 0.3, 0.9, respectively). Meningitis was associated with fourfold higher risk of PHH (HR 4.0, 95% CI 3.0, 5.2). In a multivariable analysis, dopamine was associated with reduced risk of PHH (HR 0.6; 95% CI 0.5, 0.8) and hydrocortisone with increased risk (HR 1.2; 95% CI 1.0, 1.4), which was more apparent with longer duration of treatment. Indomethacin and ibuprofen were associated with decreased risk (HR 0.7, 95% CI 0.6, 0.9; HR 0.7, 95% CI 0.4, 0.9, respectively), which was even more pronounced when given later or in multiple courses.
**Conclusions:** Several potentially modifiable exposures are associated with the development of PHH among infants with IVH. The reduced risk associated with medications such as dopamine and NSAIDs warrants further investigation.

## A168

### Long-standing overt ventriculomegaly in adults (LOVA): diagnosis and treatment options

F. Tuniz[1], A. Cramaro[1], G. Capo[1], M. Vindigni[1], D. Bagatto[2], M. Skrap[1]
[1]Neurosurgery Department, ASUI S.M. della Misericordia, Udine, Italy; [2]Neuroradiology Department, ASUI S.M. della Misericordia, Udine, Italy
**Correspondence:** F. Tuniz
*Fluids and Barriers of the CNS* 2018, **15(Suppl 1)**:A168

**Introduction:** Diagnosis and management of LOVA is still a topic of debate in the literature. This chronic hydrocephalus is presumed to begin during infancy but features of this clinical condition are evident in adulthood. This cohort study report a larger series of LOVA treated with endoscopic third ventriculostomy (ETV) and a smaller number of patients treated with a ventriculo-peritoneal (VP) shunt. Both treatments were compared and pitfalls discussed.
**Methods:** A consecutive series of 21 consecutive patients were diagnosed with LOVA using established radiological and clinical criteria. Moreover a lumbar infusion test was performed and a the intracranial pressure (ICP) was registered preoperatively. 18 patients underwent a primary ETV and only in 3 patients a VP shunt was inserted. The mean follow up period was 3.5 years.
**Results:** Preoperatively the mean ICP was higher than 12 mmHg in 80% of the patients. All the 18 patients that underwent ETV reported and improvement in their symptoms after 6 months. Only one patient worsened after 1 year which necessitated a second ETV with complete regression of the symptoms. All the three patients with shunt insertion experienced CSF overdrainage and needed multiple regulations at high level of shunt opening pressure.
**Conclusions:** Monitoring baseline ICP seems to be a promising criteria together with clinical and radiological data. The success rate for ETV was 94% and almost all the VP shunt patients experienced overdrainage. Based on our experience we believe that ETV is a very promising option for the surgical treatment of LOVA.

## A169

### Arterial spin labeling MRI (ASL) in patients affected by probable idiopathic normal pressure hydrocephalus (INPH): a quantitative qualitative cohort—prospective study

F. Tuniz[1], D. Bagatto[2], M. C. Vescovi[1], M. Maieron[3], M. C. De Colle[2], M. Skrap[1]
[1]Neurosurgery Department, ASUI S.M. della Misericordia, Udine, Italy; [2]Neuroradiology Department, ASUI S.M. della Misericordia, Udine, Italy; [3]Physics Department, ASUI S.M. della Misericordia, Udine, Italy
**Correspondence:** F. Tuniz
*Fluids and Barriers of the CNS* 2018, **15(Suppl 1)**:A169

**Introduction:** Cerebral blood flow has been demonstrated to be reduced in iNPH patients, mainly in the basal ganglia (BG) and periventricular white matter (PVWM) regions. The aim of this prospective study is to understand if perfusion (using ASL MRI) and diffusion MRI could improve the positive predictive value of the invasive tests for selecting patients for shunt surgery.
**Methods:** A total of 33 consecutive patients with diagnosis of probable iNPH were submitted to a diagnostic MR examination before and immediately after a lumbar infusion test. 27 subjects were positive to lumbar infusion test (group1) while 8 patients were negative (group2). All the MR examinations included a 3D-Pulsed ASL sequence.
**Results:** 25 patients were positive at least at one invasive test and were subjected to surgery. We observed an increase in rCBF in both periventricular and basal ganglia regions after invasive test evaluation and in the post-operative test; a decrease in ADC values in the periventricular region and an increase in ADC values in the basal ganglia region. 8 negative patients showed a reduction in rCBF in both PVWM and BG regions, a decrease in ADC values in the periventricular region.
**Conclusions**: Since the trend of rCBF acquired by perfusion ASL MRI and ADC values by diffusion MRI agreed with invasive test results and in an a small cohort even more specific than the clinical examination post test, this could be an effective method to be considered for the management of iNPH patient candidates for shunt surgery.

## A170

### Subject-specific multiporoelastic model for exploring the risk factors associated with mild cognitive impairment

J. C. Vardakis[1], L. Guo[1], T. Lassila[2], M. Mitolo[3], N. Ravikumar[4], D. Chou[5], M. Lange[2], A. S. Foroushani[2], B. J. Tully[6], Z. A. Taylor[4], S. Varma[2], A. Venneri[3,7], A. F. Frangi[2], Y. Ventikos[1]
[1]Department of Mechanical Engineering, University College London, London, UK; [2]Centre for Computational Imaging & Simulation Technologies in Biomedicine (CISTIB), Department of Electronic and Electrical Engineering, University of Sheffield, Sheffield, UK; [3]IRCCS San Camillo Foundation Hospital, Venice, Italy; [4]Centre for Computational Imaging & Simulation Technologies in Biomedicine (CISTIB), Department of Mechanical Engineering, University of Sheffield, Sheffield, UK; [5]Institute of Biomedical Engineering & Department of Engineering Science, University of Oxford, Oxford, UK; [6]Children's Medical Research Institute and School of Medical Sciences, Sydney Medical School, The University of Sydney, Westmead, Australia; [7]Department of Neuroscience, University of Sheffield, Sheffield, UK
**Correspondence:** J. C. Vardakis
*Fluids and Barriers of the CNS* 2018, **15(Suppl 1)**:A170

**Introduction:** Mild cognitive impairment (MCI) is a state between normal ageing and dementia. There is evidence to suggest that common forms of dementia (such as Alzheimer's) are vascular disorders, caused by impaired cerebral perfusion, which may be detectable in its early prodromal MCI stage. This in turn may be promoted by cardiovascular risk factors that are strongly influenced by lifestyle. In order to develop an understanding of the exact nature of such a hypothesis, a biomechanical understanding of the influence of lifestyle factors is pursued.
**Methods:** A 3D multiporoelastic model of perfused parenchymal tissue coupled with an automated image-based model personalization workflow (includes permeability tensor maps); and a subject-specific blood flow variability model were used in this study. The subject-specific datasets used in the modelling were collected as part of the VPH-DARE@IT project, and prospective data collection was conducted. Forty cases (out of 104) were used for the simulations, and involved male and female subjects, non-smokers (cognitive healthy controls and MCI cases) during two states of activity (high and low).

**Results:** Preliminary results show an increased mean arterial blood pressure, and a reduced (mean): ICP, clearance of CSF/ISF, accumulation/drainage of CSF/ISF, perfusion and tissue strain in the MCI cases ($n = 17$) compared to the control cases ($n = 23$) during states of high and low activity.

**Conclusions:** The consolidated pipeline can be used to probe the amyloid cascade and vascular hypotheses whilst simultaneously integrating the influence of lifestyle factors in the form of boundary conditions to the multiporoelastic model.

## A171

### Disproportionately enlarged subarachnoid space (DESH): an early sign of INPH

J. Varghese, L. D'Antona, C. Craven, S. Thompson, J. Funnell, L. Thorne, L. Watkins, A. Toma
Victor Horsley Department, The National Hospital for Neurology and Neurosurgery, London, UK
**Correspondence:** J. Varghese
*Fluids and Barriers of the CNS* 2018, **15(Suppl 1)**:A171

**Introduction:** The importance of the DESH sign for the prognosis of iNPH patients has been highlighted by the results of the SINPHONI trial which showed a high positive predictive value in identifying shunt responsive patients. To our knowledge, this is the first study assessing the timing of appearance of this sign on early scans of iNPH patients.

**Methods:** This is a single centre case series. Patients admitted at our institution for shunt insertion with a diagnosis of iNPH were identified from a prospectively built database. All patients with a positive DESH sign at the time of surgery with earlier scans (at least 2 years prior day of surgery) were selected. Presence of DESH on early scans was assessed.

**Results:** Thirty-one iNPH patients with positive DESH sign were identified. Six of these patients had one MRI or CT scan performed at least 2 years prior surgery. The mean age at the time of surgery was 73 years (± 6 SD), 4 were female and 2 male. The six patients had a total of 8 earlier scans (6 brain MRIs and 2 brain CTs), performed on average 4.5 years before surgery (range 2–12, median 2.5 years, 3.6 SD). All scans except one (oldest scan performed 12 years prior surgery) showed DESH sign. For one of the patients the DESH sign was evident prior to clinical presentation (5 years before).

**Conclusions:** DESH is an early sign of iNPH, which could be present before the clinical signs and symptoms of the disease.

## A172

### Does early treatment reduce mortality in normal pressure hydrocephalus?

K. Andrén, C. Wikkelsø, P. Hellstrom, M. Tullberg, D. Jaraj
NPH research team, Institute of Neuroscience and Physiology, Sahlgrens Academy, University of Gothenburg, Gothenburg, Sweden
**Correspondence:** C. Wikkelsø
*Fluids and Barriers of the CNS* 2018, **15(Suppl 1)**:A172

**Objective:** We examined the effect of early vs. delayed treatment with shunt surgery on mortality in idiopathic normal pressure hydrocephalus (iNPH).

**Introduction:** According to several uncontrolled trials, most patients improve after shunt surgery. However, randomized trials have been scarce. Furthermore, the effect of shunt surgery on mortality has previously not been studied. This observational study is based on a natural experiment in which a random group of iNPH patients were inadvertently exposed to a severe delay of treatment.

**Methods:** In 2010–2011 a group of iNPH patients (n = 33) were unintentionally exposed to a delay of treatment due to several administrative and economic shortcomings at our university hospital (Waiting time for shunt surgery 6–24 months). These were followed up and compared to patients treated in normal fashion, within 3 months (n = 69) at the same clinic. Median follow-up time was 6.2 years. The primary outcome was mortality. Kaplan–Meier survival curves and Cox proportional hazard models adjusted for several potential confounders were analyzed.

**Results:** Crude five-year mortality was 14.5% in those with early treatment compared to 44.1% in those with delayed treatment (p = 0.001). The age-adjusted hazard ratio (HR) for death, in those with delayed treatment, was 2.57 (95% CI 1.13–5.83), p = 0.024. These findings remained significant after adjusting for symptom severity at baseline, surgical complications, duration of follow-up, hypertension and cardiovascular disease.

**Conclusions:** The present data indicate that early treatment with shunt surgery might increase survival in patients with iNPH.

## A173

### Cerebrospinal fluid AB42, T-TAU, and P-TAU levels in the differential diagnosis of idiopathic normal-pressure hydrocephalus: a systematic review and meta-analysis

Y. Xing
Department of Neurology, AviationGeneral Hospital of China Medical University & Beijing Institute of Translational Medicine, Chinese Academy of Sciences, Beijing, China
**Correspondence:** Y. Xing
*Fluids and Barriers of the CNS* 2018, **15(Suppl 1)**:A173

**Introduction:** The purpose of this systematic review and meta-analysis was to evaluate the performance of CSF Aβ42, t-tau, p-tau as potential diagnostic biomarkers for iNPH and to assess their utility in distinguishing patients with iNPH from those with AD and healthy normal controls.

**Methods:** A systematic search of all relevant studies investigating reproducible CSF Aβ42, t-tau, and p-tau quantification methods was conducted in electronic databases.

**Results:** A total of 10 studies including 413 patients with iNPH, 186 patients with AD and 147 healthy controls were included in this systematic review and meta-analysis. The concentrations of CSF t-tau, and p-tau were significantly lower in iNPH patients compared to AD (p = 0.0004; $p = 0.0002$, respectively) and lower than healthy controls (p = 0.03; $p < 0.00001$, respectively). Patients with iNPH had significantly lower Aβ42 levels compared with controls (p = 0.0002), and slightly higher Aβ42 levels compared with AD patients (p = 0.05). Subgroup analyses showed that the outcomes may have been influenced by ethnicity and CSF source. Compared to AD, overall sensitivity in differentiating iNPH was 0.813 for Aβ42, 0.828 for t-tau, 0.943 for p-tau. Relative to AD, overall specificity in differentiating iNPH was 0.506 for Aβ42, 0.842 for t-tau, 0.851 for p-tau.

**Conclusions:** The results of our meta-analysis suggest that iNPH may be associated with significantly reduced levels of CSF Aβ42, t-tau and p-tau compared to the healthy normal state. Compared to AD, both t-tau and p-tau were significantly decreased in iNPH, but CSF Aβ42 was slightly increased.

## A174

### iTUG is useful for quantitative evaluation of gait disturbance in iNPH

S. Yamada, M. Ishikawa
Normal Pressure Hydrocephalus Center, Department of Neurosurgery, Rakuwakai Otowa Hospital, Kyoto, Japan
**Correspondence:** S. Yamada
*Fluids and Barriers of the CNS* 2018, **15(Suppl 1)**:A174

**Introduction:** Timed up-and-go (TUG) test is widely used to evaluate gait and balance in elderlies. For the iNPH patients with ≥ 20 s on TUG, a ≥ 5-s improvement on TUG time after tap test can be sufficiently expected to improve ≥ 10-s on TUG after shunt surgery, whereas the patients with < 20 s need the other tests or assessments. The instrumented TUG (iTUG) has been developed as technological evolution of the TUG. Therefore, we investigated the change of gait on iTUG after the tap test and shunt surgery in the iNPH patients with mild gait disturbance.

**Methods:** iPhone is embedded high-quality 3D accelerometers which can be monitored direction and acceleration. Senior-Quality (iPhone application, produced by Digital Standard, Japan) can measure iTUG and 15 steps' gait and automatically collect data in the cloud-based storage server. Gait on iTUG and 15 steps' gait were assessed in 20 patients with possible iNPH.
**Results**: We assessed 3D acceleration in each phase of standing-up, walking-forward, turning, walking-back and sitting-down on iTUG. In the typical iNPH patient who improved their gait after the tap test or shunt surgery, the forward and vertical accelerations on iTUG and 15 steps' gait were increased, whereas the horizontal acceleration was decreased.
**Conclusions:** iTUG with Senior-Quality is the useful tool for quantitative assessment of improvement of gait disturbance, especially in the iNPH patients with mild gait disturbance.

## A175

### Characterization of cardiac- and respiratory-driven CSF motions using real-time phase contrast MR imaging with correlation mapping technique

S. Yatsushiro[1], T. Tokushima[2], S. Sunohara[2], M. Matsumae[3], N. Kajihara[4], K. Kuroda[1,2]
[1]Course of Science and Technology, Graduate School of Science and Technology, Tokai University, Hiratsuka, Kanagawa, Japan; [2]Course of Electrical and Electronic Engineering, Graduate School of Engineering, Tokai University, Hiratsuka, Kanagawa, Japan; [3]Department of Neurosurgery, School of Medicine, Tokai University, Isehara, Kanagawa, Japan; [4]Department of Radiology, Tokai University Hospital, Isehara, Kanagawa, Japan
**Correspondence:** S. Yatsushiro
*Fluids and Barriers of the CNS* 2018, **15(Suppl 1)**:A175

**Introduction:** The ECG-gated magnetic resonance (MR) phase contrast (PC) is a powerful technique to visualize the spatiotemporal velocity distribution of the cerebrospinal fluid (CSF). However, the resultant cine images are too complicated for clinical diagnosis, and the respiratory-driven motion is not evaluated. To separately characterize the CSF motions driven by cardiac pulsation and respiration, asynchronous, real-time PC in conjunction with the correlation mapping technique, which quantifies the delay and correlation of the velocity waveforms, was applied to healthy subjects.
**Methods:** The real-time 2D-PC images were acquired in 12 volunteers (31 ± 13 yo). The temporal resolution of the acquisition was 217 ms (4.6 frames/s). The volunteers were instructed to continue 6-s respiration for about 1 min. The total velocity waveform was separated into the cardiac and respiratory components according to the frequency bands of the physiological signals of ECG and respiration. For each waveform component, delay and correlation between an arbitrary voxel and a reference voxel were calculated and visualized. In addition, displacements of cardiac- and respiratory-driven CSF motions were evaluated.
**Results:** Delay and correlation maps of the cardiac component appeared to be dominant in the entire intracranial space than the respiratory-driven CSF motion. On the other hand, the propagation displacement of the velocity waveform was larger in the respiration component than in the cardiac.
**Conclusions:** The present technique demonstrated the propagation properties of cardiac- and respiratory-driven CSF motions in physiological status. This technique will be applied to patients with hydrocephalus in order to reveal the change in the CSF dynamics.

## A176

### Happy shunt: a new device for treatment of hydrocephalus

S. Yavuz[1], M. O. Taskapilioglu[2], F. İlik[1]
[1]Uludag University School of Medicine, Bursa, Turkey; [2] Uludag University School of Medicine Department of Neurosurgery, Bursa, Turkey
**Correspondence:** S. Yavuz
*Fluids and Barriers of the CNS* 2018, **15(Suppl 1)**:A176

**Introduction:** Hydrocephalus is a devastating problem of both pediatric and adult population. Congenital hydrocephalus occured at a rate of 0.7 per 1000 lives and stillborn infants. Today, half of patients need revision shunt surgeries because of infection or malfunction especially under 1 years of age.
**Methods:** In this study we presented a new shunt system for reducing the rate of revision surgeries.
**Results:** In this preliminary study the HAPPY shunt reduced ventricular obstruction, infection, slit ventricular syndrome compared to novel shunt systems.
**Conclusions:** HAPPY shunt is a promising device for the treatment of hydrocephalus.

## A177

### A prospective study of the prognostic value of cerebrospinal fluid tap test in shunting for normal-pressure hydrocephalus

Y. Zheng[1], Q. Xu[2]
[1]Department of Neurosurgery, Renji Hospital, Shanghai Jiaotong University School of Medicine, Shanghai, China; [2] Department of Neurology, Renji Hospital, Shanghai Jiaotong University School of Medicine, Shanghai, China
**Correspondence:** Q. Xu
*Fluids and Barriers of the CNS* 2018, **15(Suppl 1)**:A177

**Introduction:** To investigate the prognostic value of cerebrospinal fluid tap test in shunting for normal-pressure hydrocephalus (NPH).
**Methods:** The inclusion criteria for NPH was established prospectively. All patients underwent the tap test and then shunt surgery 5 days later. Clinical changes of the classical triad of symptoms were assessed before, 24 h after the tap test and 1 week after shunting. The prognostic value of tap test in shunting for HPH was analyzed by the screening test method.
**Results:** 78 NPH patients were included in the prospective study between June of 2012 and October of 2016. The tap test was positive in 49 patients among which 47 improved after shunt, and 2 was unchanged; the tap test was negative in 27 patients among which 9 improved after shunt, and 18 were unchanged. Thus the tap test has a 83.9% sensitivity and a 90.0% specificity for predicting the shunt effectiveness. The positive predictive value of a positive tap test was 95.9%, and the predictive value of a negative tap test was 66.7%。
**Conclusions:** Cerebrospinal fluid tap test is promising predictor of shunt effectiveness for normal-pressure hydrocephalus.

Publisher’s Note

Springer Nature remains neutral with regard to jurisdictional claims in published maps and institutional affiliations.

